# Cell type-specific NRBF2 orchestrates autophagic flux and adult hippocampal neurogenesis in chronic stress-induced depression

**DOI:** 10.1038/s41421-023-00583-7

**Published:** 2023-08-29

**Authors:** Shao-Qi Zhang, Qiao Deng, Qi Zhu, Zhuang-Li Hu, Li-Hong Long, Peng-Fei Wu, Jin-Gang He, Hong-Sheng Chen, Zhenyu Yue, Jia-Hong Lu, Fang Wang, Jian-Guo Chen

**Affiliations:** 1 Department of Pharmacology, School of Basic Medicine, Tongji Medical College, Huazhong University of Science and Technology, Wuhan, Hubei China; 2 State Key Laboratory of Quality Research in Chinese Medicine, Institute of Chinese Medical Sciences, University of Macau, Zhuhai, Macau SAR China; 3 The Key Laboratory of Neurological Diseases (HUST), Ministry of Education of China, Wuhan, Hubei China; 4 Laboratory of Neuropsychiatric Diseases, The Institute of Brain Research, Huazhong University of Science and Technology, Wuhan, Hubei China; 5 The Key Laboratory for Drug Target Researches and Pharmacodynamic Evaluation of Hubei Province, Wuhan, Hubei China; 6 Department of Neurology, Friedman Brain Institute, Icahn School of Medicine at Mount Sinai, New York, NY USA

**Keywords:** Neural stem cells, Macroautophagy

## Abstract

Dysfunctional autophagy and impairment of adult hippocampal neurogenesis (AHN) each contribute to the pathogenesis of major depressive disorder (MDD). However, whether dysfunctional autophagy is linked to aberrant AHN underlying MDD remains unclear. Here we demonstrate that the expression of nuclear receptor binding factor 2 (NRBF2), a component of autophagy-associated PIK3C3/VPS34-containing phosphatidylinositol 3-kinase complex, is attenuated in the dentate gyrus (DG) under chronic stress. NRBF2 deficiency inhibits the activity of the VPS34 complex and impairs autophagic flux in adult neural stem cells (aNSCs). Moreover, loss of NRBF2 disrupts the neurogenesis-related protein network and causes exhaustion of aNSC pool, leading to the depression-like phenotype. Strikingly, overexpressing NRBF2 in aNSCs of the DG is sufficient to rescue impaired AHN and depression-like phenotype of mice. Our findings reveal a significant role of NRBF2-dependent autophagy in preventing chronic stress-induced AHN impairment and suggest the therapeutic potential of targeting NRBF2 in MDD treatment.

## Introduction

Major depressive disorder (MDD) is a highly prevalent and heterogeneous neuropsychiatric disease that inflicts ～350 million people worldwide^1^. Current treatments for MDD have been found to be insufficient, such as slow onset and low rates of complete remission^2^, emphasizing the urgent need for alternative targeting treatment strategies. Postmortem studies of untreated MDD patients demonstrate that both adult neural progenitors and granule cells in dentate gyrus (DG), a hippocampal neurogenic niche, are overwhelmingly deficient^3–5^. Previous works showed that adult neurogenesis is essential for the action of antidepressants^6,7^. Adult hippocampal neurogenesis (AHN) is a process that produces newborn neurons in the DG throughout life, providing a substantial basis for structural and functional plasticity in the hippocampus^8,9^. In the mammalian brain, adult neural stem cells (aNSCs) in the subgranular zone (SGZ) of the DG undergo self-renewal and differentiation into proliferating intermediate progenitor cells (IPCs), neuroblasts, immature neurons and eventually give rise to newborn neurons^10^. Adult neurogenesis in SGZ, but not the subventricular zone of the lateral ventricle, seems to be highly responsive to both stress and antidepressant treatment^6,11^. Therefore, understanding how the fate of AHN is affected by chronic stress is imperative for improving neurogenesis and the therapeutic strategy of MDD.

Macroautophagy (autophagy hereafter) is a conserved cellular pathway that degrades misfolded or aggregated proteins and impaired organelles through lysosomes^12,13^. Autophagy is divided into several phases: initiation, autophagosome formation, autophagosome maturation, and degradation of cargo by the lysosome. Autophagy initiation is regulated by several protein complexes, including the PIK3C3/VPS34-containing class III phosphatidylinositol 3-kinase (PtdIns3K) complex. PtdIns3K complex contains BECN1/Beclin 1, PIK3R4/VPS15, and ATG14L/Barkor^14–17^. Nuclear receptor binding factor 2 (NRBF2) is a novel component of class III PtdIns3K complex, and directly interacts with ATG14L and then enhances the activity of ATG14L-linked VPS34 kinase to facilitate autophagy initiation^18–20^. NRBF2, as a RAB7 effector, is also required for autophagosome maturation^21^.

Autophagy dysfunction has been implicated in the pathogenesis of neuropsychiatric disorders, such as MDD and cocaine addiction^22,23^. Gulbins et al. has reported that inhibition of autophagy abolishes the effects of antidepressants on depression-like behavior and AHN impairment^24^. One study has shown that autophagy plays an important role in the maintenance and differentiation of postnatal neural stem cells^25^. Meanwhile, conditional deletion of autophagy-related gene ATG5 in aNSCs impairs autophagic flux and adult neurogenesis^26^. Pharmacological inhibition of autophagy with PI3K-III inhibitors 3-methyladenine suppresses the proliferation and differentiation of aNSCs^27^. Recent single-cell RNA sequencing data indicate that NRBF2 is expressed in the aNSCs of the DG among macaques and mice^28–30^. However, whether and how NRBF2 regulates AHN in chronic stress-induced depression remains unclear.

Here, we observed the reduction of NRBF2 expression in the DG of stressed mice, which is correlated with autophagy deficiency. Furthermore, NRBF2 deficiency inhibited autophagosome formation in aNSCs via suppressing the activity of PtdIns3K complex, disrupted neurogenesis-related protein network, and drove the exhaustion of aNSCs pool, a significant factor contributing to the AHN impairment and depression-like behavior induced by chronic stress. Importantly, overexpression of NRBF2, specifically in aNSCs of the DG, rescued the defective AHN and depression-like behavior of NRBF2 knockout (NRBF2^*−/−*^) mice or chronic social defeat stress (CSDS)-exposed mice. Our findings unveil a previously uncharacterized role of NRBF2 in regulating depression-like behavior in an AHN-dependent manner.

## Results

### Chronic stress induces a decrease in NRBF2 levels in the DG of adult mice

**Fig. 1:**
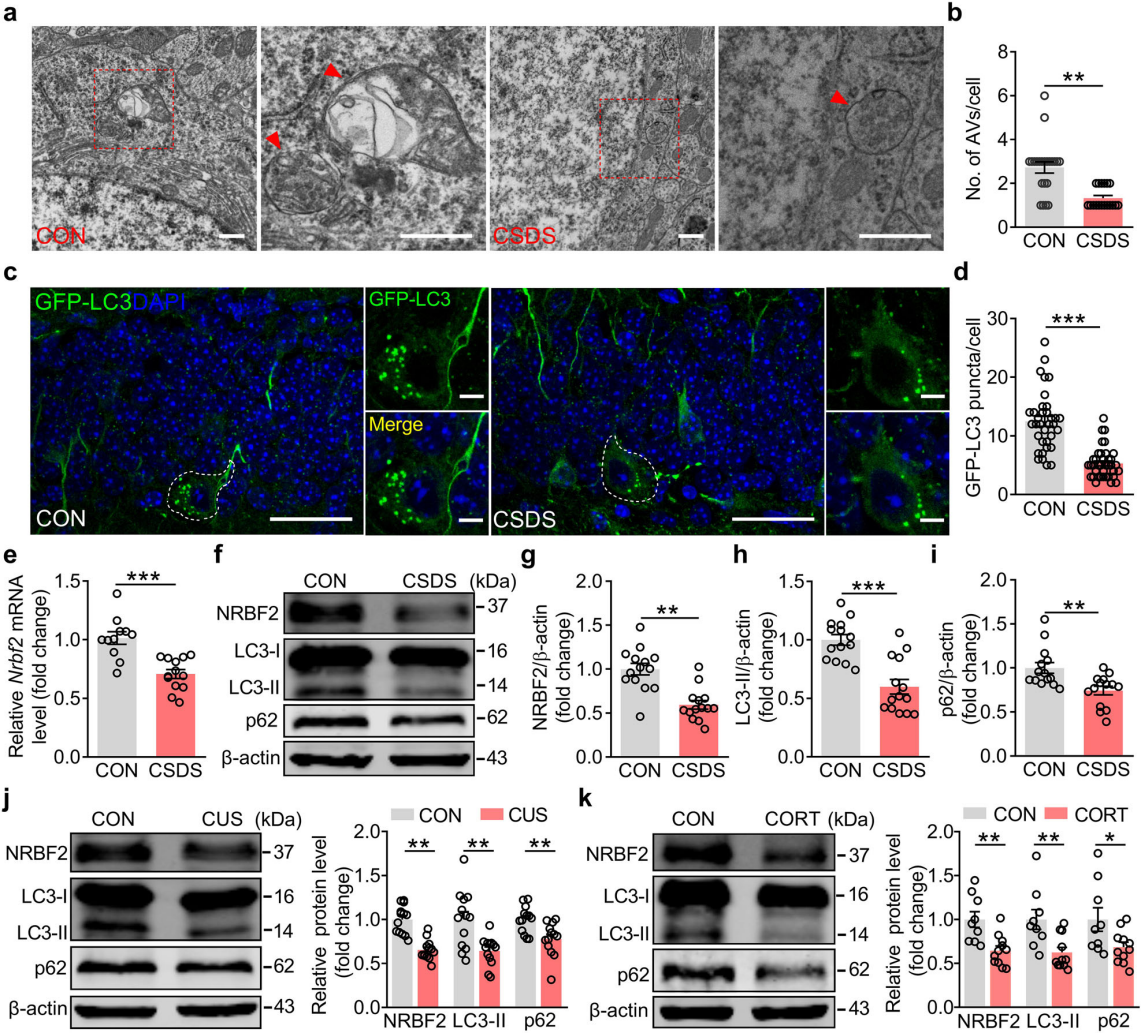
Chronic stress induces a decrease in NRBF2 level in the dentate gyrus of adult mice. **a**, **b** Representative electron micrographs (**a**) and quantification (**b**) of autophagic vacuoles (AVs) in the DG of control (CON) and CSDS-exposed mice (*n* = 18–22 cells per group). Higher magnification views of selected regions (red square). Red arrows indicate autophagic vacuoles. Scale bars, 0.5 μm. **c**, **d** Representative images (**c**) and quantification (**d**) of GFP-LC3 puncta in the DG (*n* = 35–38 cells per group). White outlines represent GFP-LC3^+^ cells. Scale bars, 30 μm (overview) and 5 μm (zoom). **e** Quantitative PCR results showing the *Nrbf2* mRNA level in the DG (*n* = 11–13 mice per group). **f**–**i** Representative images of western blotting (**f**) and quantification of NRBF2 (**g**), LC3-II (**h**), and p62 (**i**) protein expression in the DG (*n* = 14 mice per group). **j**, **k** Western blotting analysis of NRBF2, LC3-II, and p62 protein expression in the DG of control, CUS- or CORT-treated mice (*n* = 9–13 mice per group). Data are presented as means ± SEM and analyzed by two-sided unpaired *t*-test (**b**, **d**, **e**, **g**–**k**). *P < 0.05, **P < 0.01, and ***P < 0.001.

Emerging evidence supports that chronic stress-induced AHN impairment is implicated in the pathogenesis of depression^31,32^. To assess whether autophagy acted as an intermediate hinge to intertwine chronic stress with defective AHN, adult C57BL/6J mice were exposed to CSDS for 10 days to induce depression-like behavior (Supplementary Fig. S1a). The CSDS-exposed mice exhibited depression-like behavior, including reduced social interaction ratio in the social interaction test (SIT), anhedonia in the sucrose preference test (SPT), and increased immobility time in tail suspension test (TST) and forced swim test (FST) (Supplementary Fig. S1b–f). The autophagic structures in the DG were detected by transmission electron microscope. It was shown that the CSDS-exposed mice exhibited a significant reduction in the number of autophagic vacuoles (Fig. 1a, b). Microtubule-associated protein 1 light chain 3 (LC3), the primary Atg8-family homolog and the reliable autophagy marker in mammalian cells, is involved in autophagosome formation^33^. Next, the lentivirus vector expressing green fluorescent protein-tagged LC3 (LV-GFP-LC3) was microinjected into DG to trace autophagosomes 2 weeks before the CSDS. It was found that the GFP-LC3 puncta in the DG were significantly decreased in the CSDS-exposed mice compared with that in control mice (Fig. 1c, d).

Considering that chronic stress impaired autophagic structures of the DG, we wondered whether chronic stress could change the level of autophagy-related protein NRBF2. Quantitative PCR (qPCR) analysis showed that the level of *Nrbf2* mRNA was much higher in DG than in other brain regions (Supplementary Fig. S1g). Therefore, the change in the *Nrbf2* level in the DG was measured. It was found that the levels of *Nrbf2* mRNA and protein were attenuated in CSDS-exposed mice compared with that of control mice (Fig. 1e–g), accompanied by the decreased expression of LC3-II protein (Fig. 1h). The mRNA and protein levels, as well as the fluorescence intensity of sequestosome 1/p62 (SQSTM1/p62), were reduced in the DG of CSDS-exposed mice (Fig. 1i; Supplementary Fig. S1h–j), suggesting that the reduced expression of p62 resulted from its downregulated transcriptional level. Meanwhile, the expressions of other components in the VPS34 complex, such as VPS34, Beclin1, and ATG14L, were unaltered in the DG of CSDS-exposed mice (Supplementary Fig. S1k–n), indicating that chronic stress only affects the level of NRBF2 protein of the VPS34 complex.

To confirm the response of NRBF2 expression under chronic stress conditions, we employed other animal models of depression, including chronic unpredictable stress (CUS)- and chronic corticosterone (CORT)-induced depression^24^. Similarly, we observed a decrease in the expressions of NRBF2, LC3-II, and p62 proteins in the DG of CUS- and CORT-treated mice, accompanied by depression-like behavior (Fig. 1j, k; Supplementary Fig. S1o–t). These results suggest that NRBF2 level in the DG negatively correlated with depression-like behavior induced by chronic stress.

### NRBF2 overexpression in the DG improves CSDS-induced depression-like behavior in an AHN-dependent manner

**Fig. 2:**
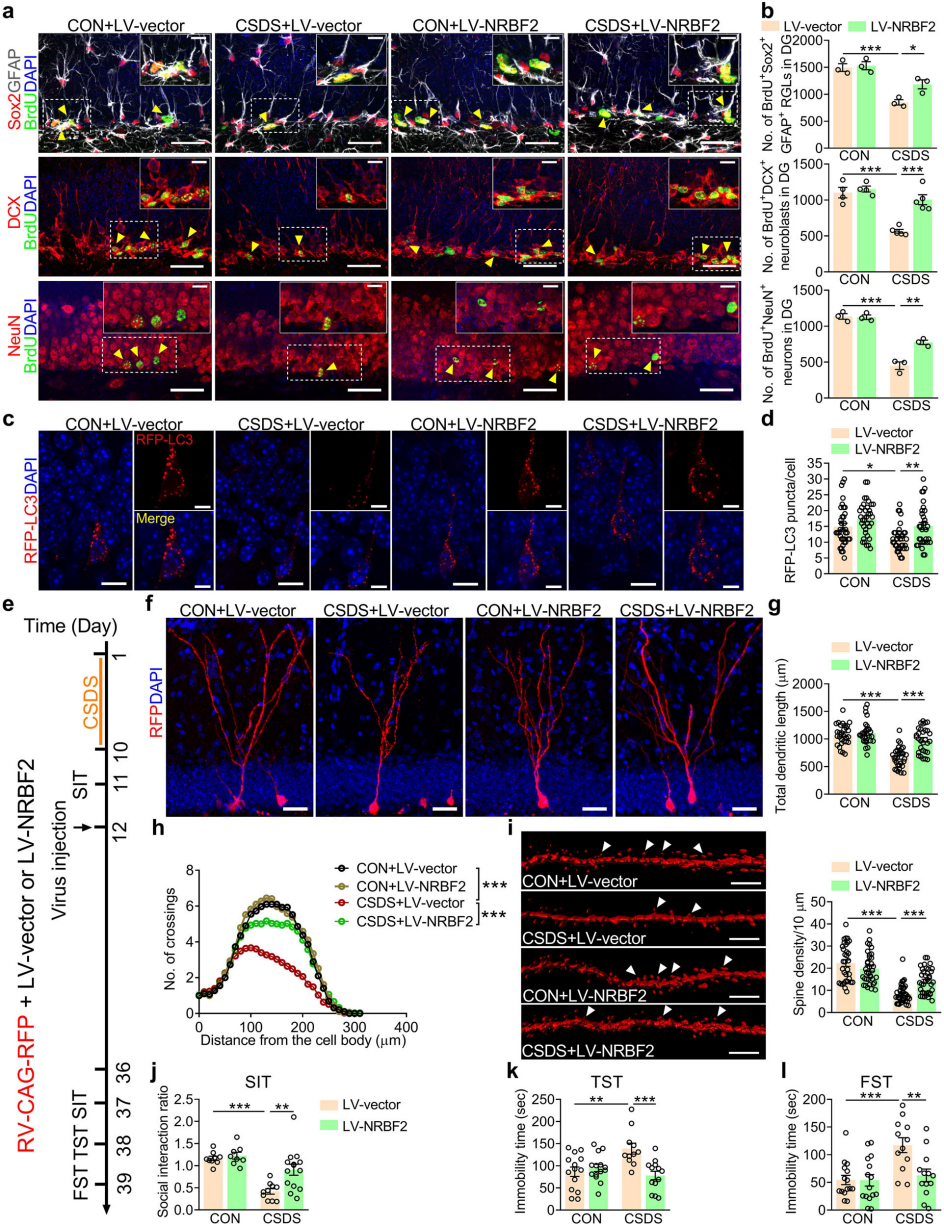
Overexpression of NRBF2 in the DG improves CSDS-induced AHN impairment and depression-like behavior of mice. **a** Representative images of BrdU^+^Sox2^+^GFAP^+^ RGLs, BrdU^+^DCX^+^ neuroblasts, and BrdU^+^NeuN^+^ neurons in the DG of control and CSDS-exposed mice treated with LV-vector or LV-NRBF2. Higher magnification views of selected regions (white rectangle). Yellow arrows indicate BrdU^+^ and marker^+^ cells. Scale bars, 30 μm (overview) and 10 μm (inset). **b** Quantification of BrdU^+^Sox2^+^GFAP^+^ RGLs, BrdU^+^DCX^+^ neuroblasts, and BrdU^+^NeuN^+^ neurons in the DG (*n* = 3–5 mice per group). **c**, **d** Representative image (**c**) and quantification (**d**) of RFP-LC3 puncta in the DG (*n* = 32–36 cells per group). Scale bars, 10 μm (overview) and 5 μm (zoom). **e** Timeline of experiments. **f**–**h** Representative images (**f**) and quantification of dendritic length (**g**) and dendritic complexity (**h**) in RFP^+^ newborn neurons (*n* = 29–33 cells per group). Scale bars, 30 μm. **i** Representative images and quantification of dendritic spine density of newborn neurons (*n* = 33–37 segments per group). White arrows indicating spines. Scale bars, 5 μm. **j**–**l** Behavioral tests of SIT (**j**), TST (**k**), and FST (**l**) in control and CSDS-exposed mice treated with LV-vector or LV-NRBF2 (*n* = 8–15 mice per group). Data are presented as means ± SEM and analyzed by two-way ANOVA followed by Bonferroni’s post hoc test (**b**, **d**, **g**, **i**–**l**) or repeated measures ANOVA (**h**). *P < 0.05, **P < 0.01, and ***P < 0.001.

We next assessed the localization of NRBF2 in adult DG via cell type-specific marker. The results showed that the NRBF2 expression was detected in both Sox2^+^GFAP^+^ type 1 and Sox2^+^GFAP^−^ type 2 NSCs, as well as Tbr2^+^ IPCs, DCX^+^ neuroblasts and NeuN^+^ neurons in the adult hippocampus (Supplementary Fig. S2a–d), indicating that NRBF2 is enriched in adult hippocampal neurogenic lineage. Then, we asked whether chronic stress-induced deficits of NRBF2 in the DG were associated with the impairment of AHN in the rodent model of depression. Bromodeoxyuridine (BrdU, 200 mg/kg) was intraperitoneally (i.p.) injected, and brain tissue was collected 2 h later for BrdU labeling assay^34^. Quantitative analysis showed that the numbers of BrdU^+^Sox2^+^GFAP^+^ radial glia-like cells (RGLs) and BrdU^+^DCX^+^ neuroblasts were reduced in CSDS-exposed mice compared with that in control mice (Supplementary Fig. S3a–d). Retrovirus expressing red fluorescent protein (RV-RFP) was microinjected into DG to label newborn neurons^35^, and it was found that RFP^+^ newborn neurons in the DG of CSDS-exposed mice exhibited shorter dendrites and less dendritic complexity four weeks after retrovirus injection than that of control mice (Supplementary Fig. S3e, f). Additionally, the spine density of RFP^+^ newborn neurons in the DG of CSDS-exposed mice was significantly lower than that of control mice (Supplementary Fig. S3g, h). However, lentivirus-mediated overexpression of NRBF2 (LV-NRBF2) in the DG increased the number of BrdU^+^Sox2^+^GFAP^+^ RGLs, BrdU^+^DCX^+^ neuroblasts, and BrdU^+^NeuN^+^ neurons of CSDS-exposed mice (Fig. 2a, b), accompanied with the increase in the expression of LC3-II protein and the decrease in the expression of p62 protein (Supplementary Fig. S4a–d), and the increase in the number of RFP-LC3 puncta in the DG of CSDS-exposed mice (Fig. 2c, d). Meanwhile, LV-NRBF2 increased the total dendritic length, the number of dendritic intersections, and the density of the dendritic spine in newborn neurons of CSDS-exposed mice (Fig. 2e–i). As shown in Fig. 2j–l, overexpression of NRBF2 alleviated the depression-like behavior induced by CSDS, including the increased social interaction ratio in SIT and decreased immobility time in TST and FST. These results indicate that NRBF2 overexpression in the DG ameliorates CSDS-induced autophagy deficiency and AHN impairment.

To further address the possible role of AHN in NRBF2 overexpression-mediated antidepressant-like effect, the DNA-alkylating agent temozolomide (25 mg/kg), a suppressor of adult neurogenesis^36^ was i.p. injected (Supplementary Fig. S5a). Our results showed that temozolomide abrogated NRBF2 overexpression-induced antidepressant-like and proneurogenic effects in CSDS-exposed mice (Supplementary Fig. S5b–d), providing evidence that AHN is a crucial target of NRBF2-mediated regulation of depression-like behavior. Collectively, these results suggest that overexpression of NRBF2 in the DG ameliorates CSDS-induced depression-like behavior in an AHN-dependent manner.

### Overexpression of NRBF2, specifically in aNSCs of DG, rescues the impaired AHN and depression-like phenotype of NRBF2 knockout mice

**Fig. 3:**
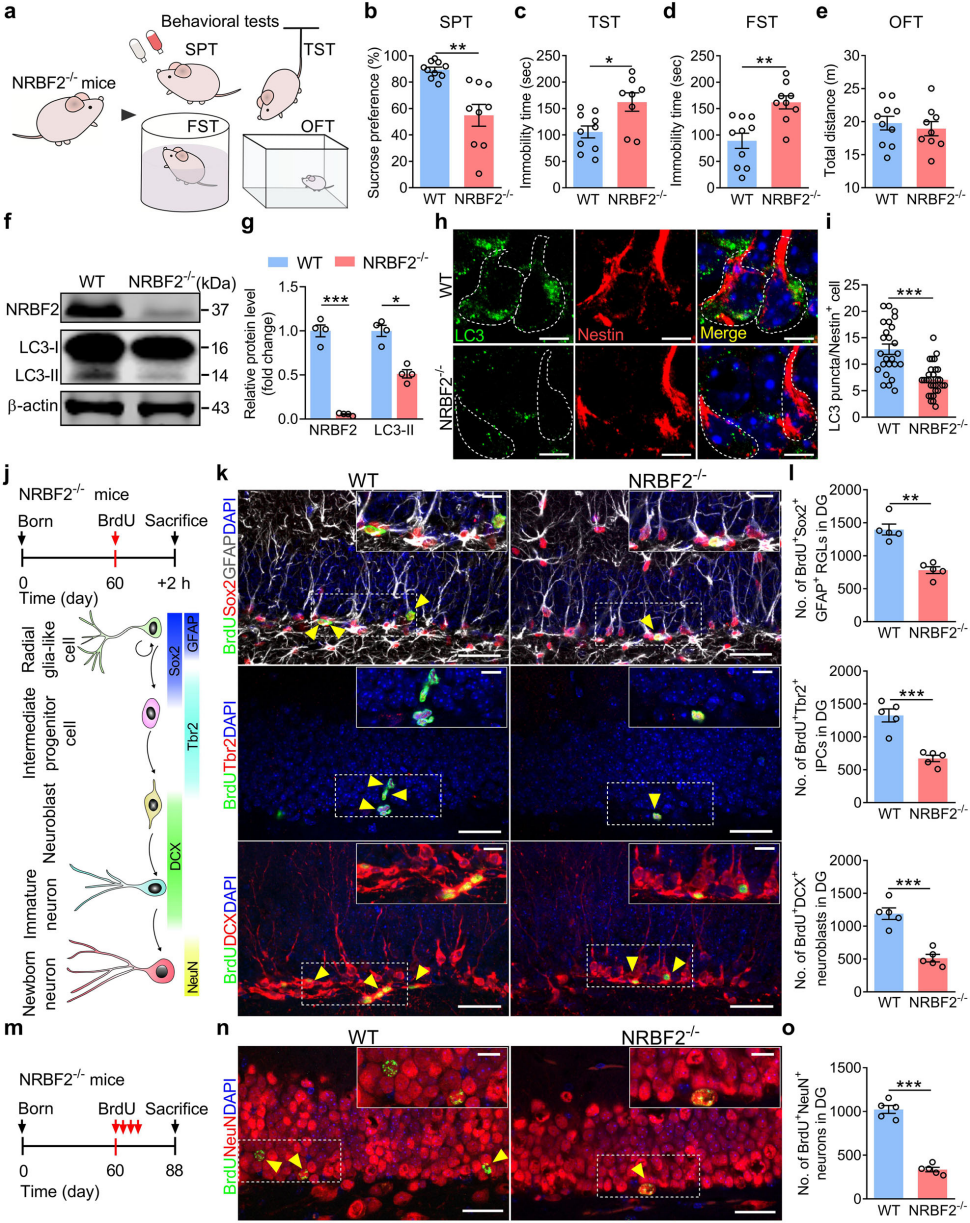
NRBF2 deletion inhibits AHN and leads to depression-like behavior in mice. **a** Experimental paradigms for behavioral tests. **b**–**e** Behavioral tests of SPT (**b**), TST (**c**), FST (**d**), and OFT (**e**) in WT and NRBF2^*−/−*^ mice (*n* = 8–10 mice per group). **f**, **g** Representative images (**f**) and quantification (**g**) of NRBF2 and LC3-II protein expression in the DG (*n* = 4 mice per group). **h**, **i** Representative images (**h**) and quantification (**i**) of LC3 puncta within the soma of Nestin^+^ cells in the DG (*n* = 25–29 cells per group). White outlines represent the soma of Nestin^+^ cells. LC3 (green), Nestin (red), and DAPI (blue). Scale bars, 5 μm. **j** A schematic diagram of the experimental design of a 2-h BrdU (200 mg/kg, i.p.) pulse-chase (upper). A schematic diagram of cell lineage-specific markers during AHN (bottom). **k**, **l** Representative images (**k**) and quantification (**l**) of BrdU^+^Sox2^+^GFAP^+^ RGLs, BrdU^+^Tbr2^+^ IPCs, and BrdU^+^DCX^+^ neuroblasts in the DG (*n* = 5 mice per group). Higher magnification views of selected regions (white rectangle). Yellow arrows indicate BrdU^+^ and marker^+^ cells. Scale bars, 30 μm (overview) and 10 μm (inset). **m** A schematic diagram of the experimental design of a 30-day BrdU pulse-chase. **n**, **o** Representative images (**n**) and quantification (**o**) of BrdU^+^NeuN^+^ neurons in the DG (*n* = 5 mice per group). Higher magnification views of selected regions (white rectangle). Yellow arrows indicate BrdU^+^NeuN^+^ neurons. Scale bars, 30 μm (overview) and 10 μm (inset). Data are presented as means ± SEM and analyzed by two-sided unpaired *t*-test (**b**–**e**, **g**, **i**, **l**, **o**). *P < 0.05, **P < 0.01, and ***P < 0.001.

Considering that overexpression of NRBF2 produced an antidepressant-like effect, NRBF2^*−/−*^ mice were employed to investigate whether NRBF2 deficiency could induce depression-like behavior (Fig. 3a). The results showed that deletion of NRBF2 decreased sucrose preference in SPT and increased the immobility time in both TST and FST (Fig. 3b–d), with no difference of locomotor activity and center exploration in open field test (OFT) (Fig. 3e; Supplemental Fig. S6a–c), suggesting that deletion of NRBF2 gene leads to the depression-like phenotype, but not anxiety-like behavior, of mice. Meanwhile, NRBF2^*−/−*^ mice displayed a reduced expression of LC3-II in the DG (Fig. 3f, g). Furthermore, we found that the LC3 puncta within the soma of Nestin^+^ cells were significantly decreased in the DG of NRBF2^*−/−*^ mice compared with wild-type (WT) littermates (Fig. 3h, i).

Therefore, the fate mapping of aNSCs was performed by using lineage markers to determine the changes in adult neurogenesis in the DG of NRBF2^*−/−*^ mice (Fig. 3j). Quantification assay revealed that the number of BrdU^+^Sox2^+^GFAP^+^ RGLs, BrdU^+^Tbr2^+^ IPCs, and BrdU^+^DCX^+^ neuroblasts were significantly reduced in NRBF2^*−/−*^ mice compared with that of WT littermates (Fig. 3k, l). To evaluate the generation of newborn neurons, NRBF2^*−/−*^ mice received BrdU injection (50 mg/kg, i.p.) twice per day for 2 days, and BrdU incorporation into newborn neurons was measured 4 weeks after the final injection (Fig. 3m). The result revealed a decrease in the number of BrdU^+^NeuN^+^ neurons in NRBF2^*−/−*^ mice (Fig. 3n, o).

**Fig. 4:**
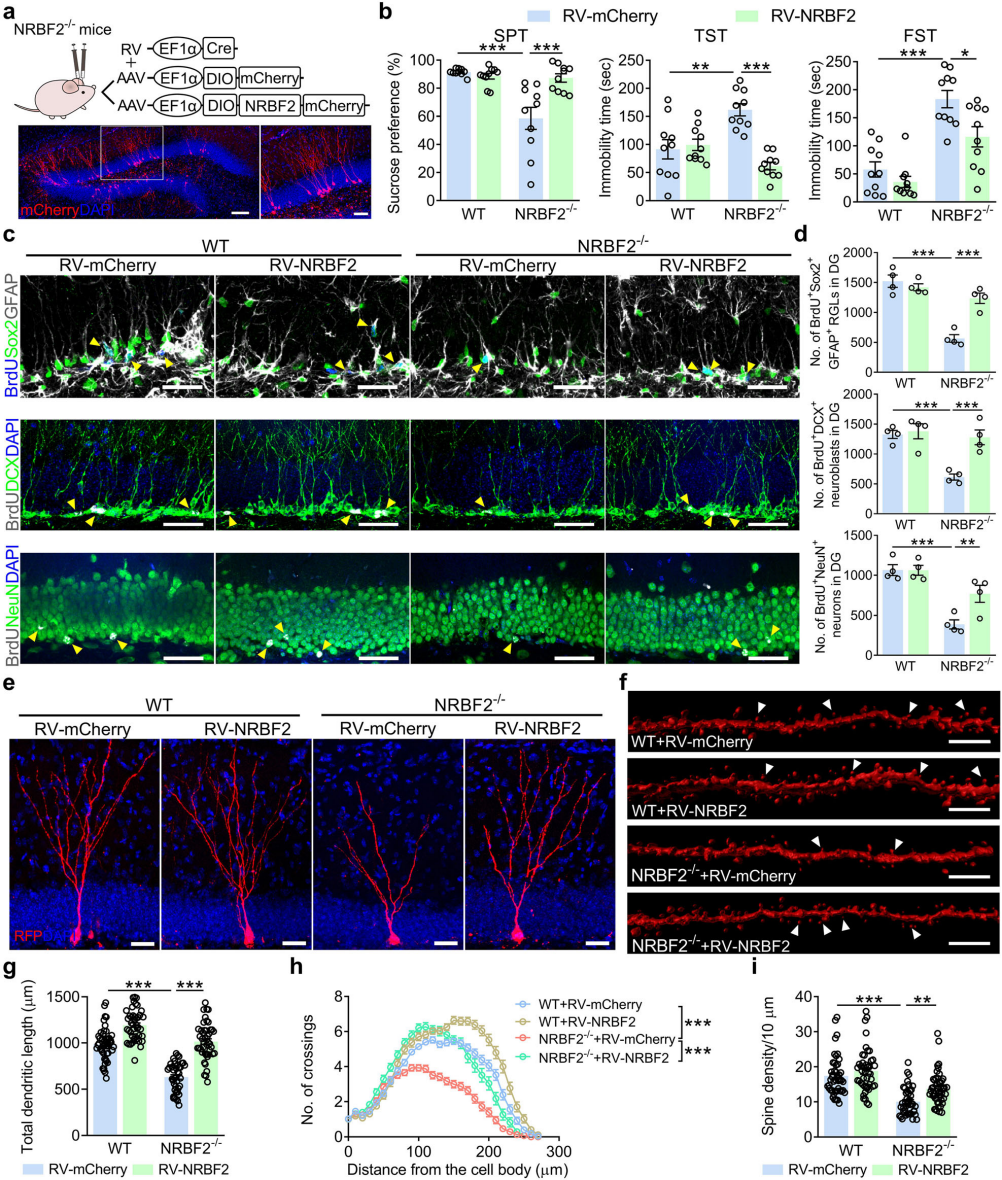
Overexpression of NRBF2 in aNSCs rescues the impaired AHN and depression-like phenotype of NRBF2 knockout mice. **a** A combinative use of RV-EF1α-Cre and AAV-EF1α-DIO-NRBF2-mCherry was applied for selective overexpression of NRBF2 in aNSCs of DG (upper). Representative image of mCherry expression in the DG (bottom). Higher magnification views of selected regions (white square). Scale bars, 100 μm (overview) and 50 μm (zoom). **b** Behavioral tests of SPT, TST, and FST on WT and NRBF2^*−/−*^ mice treated with RV-mCherry or RV-NRBF2 (*n* = 10 mice per group). **c** Representative images of BrdU^+^Sox2^+^GFAP^+^ RGLs, BrdU^+^DCX^+^ neuroblasts, and BrdU^+^NeuN^+^ neurons in the DG. Yellow arrows indicate BrdU^+^ and marker^+^ cells. Scale bars, 30 μm. **d** Quantification of BrdU^+^Sox2^+^GFAP^+^ RGLs, BrdU^+^DCX^+^ neuroblasts, and BrdU^+^NeuN^+^ neurons in the DG (*n* = 4 mice per group). **e** Representative images of mCherry^+^ newborn neurons. Scale bars, 30 μm. **f** Representative images of the dendritic spine. White arrows indicate spines. Scale bars, 5 μm. **g**, **h** Quantification of the dendritic length (**g**) and dendritic complexity (**h**) of mCherry^+^ newborn neurons (*n* = 41–49 cells per group). **i** Quantification of dendritic spine density of mCherry^+^ newborn neurons (*n* = 44–48 segments per group). Data are presented as means ± SEM and analyzed by two-way ANOVA followed by Bonferroni’s post hoc test (**b**, **d**, **g**, **i**) or repeated measures ANOVA (**h**). *P < 0.05, **P < 0.01, and ***P < 0.001.

Considering that NRBF2^*−/−*^ mice exhibited depression-like behavior and the deficit in AHN, we wondered whether restoring the expression of NRBF2 in aNSCs could rescue the depression-like phenotype in NRBF2^*−/−*^ mice. The retroviral approach (a viral cocktail of RV-EF1α-Cre with adeno-associated virus (AAV)-EF1α-DIO-NRBF2, RV-NRBF2) was applied to ensure specific overexpression of NRBF2 in the aNSCs of DG in NRBF2^*−/−*^ mice (Fig. 4a). It was found that specific overexpression of NRBF2 in aNSCs of DG rescued the depression-like phenotype of NRBF2^*−/−*^ mice, including the increase in sucrose preference in SPT, and the decrease in immobility time in TST and FST (Fig. 4b). Furthermore, specific overexpression of NRBF2 in DG aNSCs increased the number of BrdU^+^Sox2^+^GFAP^+^ RGLs, BrdU^+^DCX^+^ neuroblasts, and BrdU^+^NeuN^+^ neurons in the DG of NRBF2^*−/−*^ mice (Fig. 4c, d), accompanied with the increase in total dendrite length, the dendritic complexity, and the dendritic spine density of retrovirus-infected newborn neurons (Fig. 4e–i). These results suggest that overexpression of NRBF2 in aNSCs of DG rescues the AHN deficits and depression-like phenotype in NRBF2^*−/−*^ mice.

### NRBF2 deficiency impairs the autophagosome formation and neurogenesis of aNSCs

We next explored the potential role of NRBF2-dependent autophagy in the proliferation, differentiation, and survival of aNSCs. aNSCs, identified by neural stem cell markers Nestin and Sox2 (Supplementary Fig. S7a, b), were isolated from the DG of 8-week-old C57BL/6J mice and transfected with lentivirus containing shRNA targeting NRBF2 (LV-sh*Nrbf2*). We observed the reduction of NRBF2, LC3-II, and p62 protein levels in the LV-sh*Nrbf2* group, suggesting that NRBF2 deficiency inhibits the autophagic process in aNSCs (Supplementary Fig. S7c, d). Meanwhile, NRBF2-deficient aNSCs generated smaller and fewer neurospheres with a lower percentage of BrdU^+^ cells than that of control aNSCs, indicating the defects in aNSCs proliferation induced by knockdown of NRBF2 in aNSCs (Supplementary Fig. S7e–h). We then examined the role of NRBF2 in aNSCs differentiation in vitro and found that NRBF2-deficient aNSCs produced fewer Tuj1^+^ neurons than control aNSCs, without changes in the percentage of GFAP^+^ astrocytes (Supplementary Fig. S7i, j). In addition, there were no differences in activated caspase-3 (AC-3)-positive apoptotic cells and differentiated progeny between control and NRBF2-deficient aNSCs (Supplementary Fig. S7k–m). These results indicate that NRBF2 deficiency inhibits the proliferation and neuronal differentiation of aNSCs, with little effect on astrocytic differentiation and cell apoptosis.

**Fig. 5:**
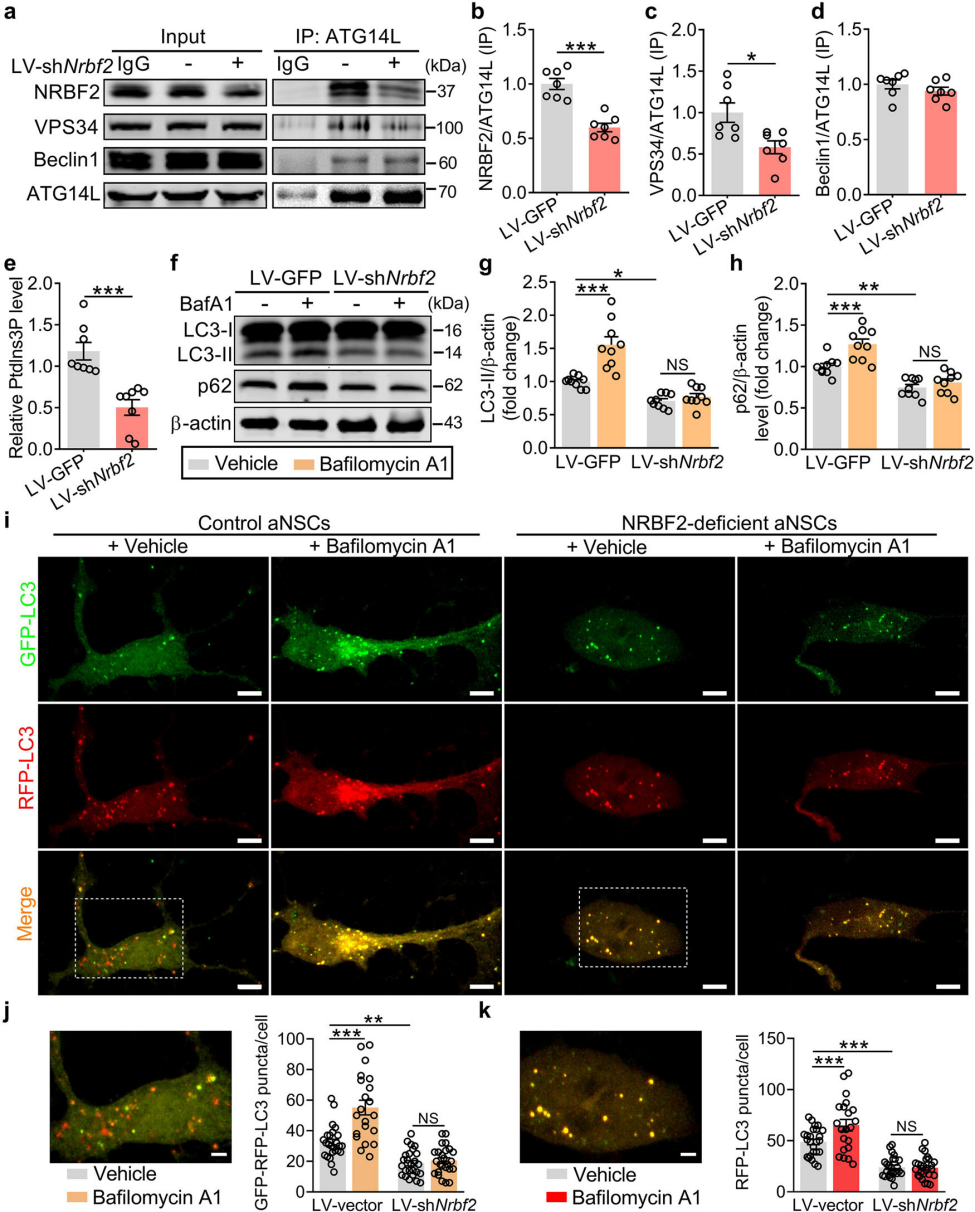
NRBF2 deficiency impairs the autophagosome formation and neurogenesis of aNSCs. **a**–**d** Immunoprecipitation (IP) and western blotting analysis (**a**) of the interaction between NRBF2 (**b**), VPS34 (**c**), and Beclin1 (**d**) with ATG14L in control and NRBF2-deficient aNSCs (*n* = 7 independent experiments per group). **e** Quantification of ATG14L-immunoprecipitated VPS34 kinase activity (*n* = 8 independent experiments per group). **f**–**h** Representative images of western blotting analysis (**f**) and quantification of LC3-II (**g**) and p62 (**h**) in control and NRBF2-deficient aNSCs treated with vehicle (0.1% DMSO) or bafilomycin A1 (BafA1) (*n* = 9 independent experiments per group). **i**–**k** Representative images (**i**) and quantitation of GFP-RFP-LC3 puncta (**j**) or RFP-LC3 puncta (**k**) (*n* = 21–25 cells per group). Higher magnification views of selected regions (white rectangle). Scale bars, 5 μm (overview) and 2 μm (zoom). Data are presented as means ± SEM and analyzed by two-sided unpaired *t*-test (**b**–**e**) or two-way ANOVA followed by Bonferroni’s post hoc test (**g**, **h**, **j**, **k**). *P < 0.05, **P < 0.01, and ***P < 0.001. NS indicates no significant difference.

Considering that NRBF2 is a component of the specific PI3K-III complex^14^, we asked whether VPS34/PI3K3C complex-dependent autophagy was involved in the role of NRBF2 in aNSCs. The immunoprecipitation results showed that the level of precipitated NRBF2 and VPS34, rather than precipitated Beclin1, was significantly reduced in NRBF2-deficient aNSCs when normalized against immunoprecipitated ATG14L (Fig. 5a–d). We also analyzed the activity of precipitated lipid kinase VPS34 by phosphatidylinositol 3-phosphate (PtdIns3P) ELISA kit, and the result showed that the ATG14L-associated VPS34 activity was markedly lower in NRBF2-deficient aNSCs than that of control aNSCs (Fig. 5e). Next, the effect of aNSCs-specific deficiency of NRBF2 on the autophagy–lysosome pathway was evaluated by using a 12 h pulse of bafilomycin A1 (20 nM), an inhibitor of V-ATPase-dependent acidification and autophagosome-lysosome fusion that resulted in accumulation of autophagy biomarker^37^. It was found that bafilomycin A1 led to the accumulation of LC3-II and p62 in control aNSCs, but failed to affect the reduction of LC3-II and p62 in NRBF2-deficient aNSCs (Fig. 5f–h). Meanwhile, bafilomycin A1 treatment for 12 h did not affect the cell viability of aNSCs (Supplementary Fig. S7n). Moreover, RFP-GFP tandem fluorescent-tagged LC3 (RFP-GFP-LC3) was employed to investigate the autophagic flux based on the pH sensitivity differences exhibited by GFP and RFP. It was found that bafilomycin A1 led to the accumulation of GFP-RFP-LC3 and RFP-LC3 puncta in control aNSCs, but failed to affect the reduction of GFP-RFP-LC3 and RFP-LC3 puncta in NRBF2-deficient aNSCs (Fig. 5i–k), indicating that the deficiency of NRBF2 induces the impairment of autophagic flux through inhibition of autophagosome formation. Collectively, these results support the hypothesis that aNSCs-specific deficiency in NRBF2 results in impaired autophagic flux via weakening the ATG14L-associated VPS34 activity.

### Selective VPS34 inhibitor SAR405 facilitates AHN impairment and stress susceptibility of mice

It has been demonstrated that SAR405 is a potent and highly selective VPS34 inhibitor that directly affects the catalytic activity of VPS34^38,39^. Thus, we wondered whether SAR405 mediated the behavioral abnormality and AHN impairment induced by chronic stress. The cultured aNSCs were treated with SAR405 (1 μM) for 24 h in vitro, and it was found that compared with vehicle treatment, SAR405 reduced the expressions of LC3-II protein and increased the expression of p62 protein in the aNSCs, which were abolished by pretreatment with LV-NRBF2 (Supplementary Fig. S8a–d). Furthermore, SAR405 decreased the percentage of BrdU^+^ cells in the control aNSCs, which was prevented by pretreatment with NRBF2 overexpression (Supplementary Fig. S8e, f). These results provide in vitro evidence that NRBF2 alleviates SAR405-induced impairment of the proliferation of aNSCs.

**Fig. 6:**
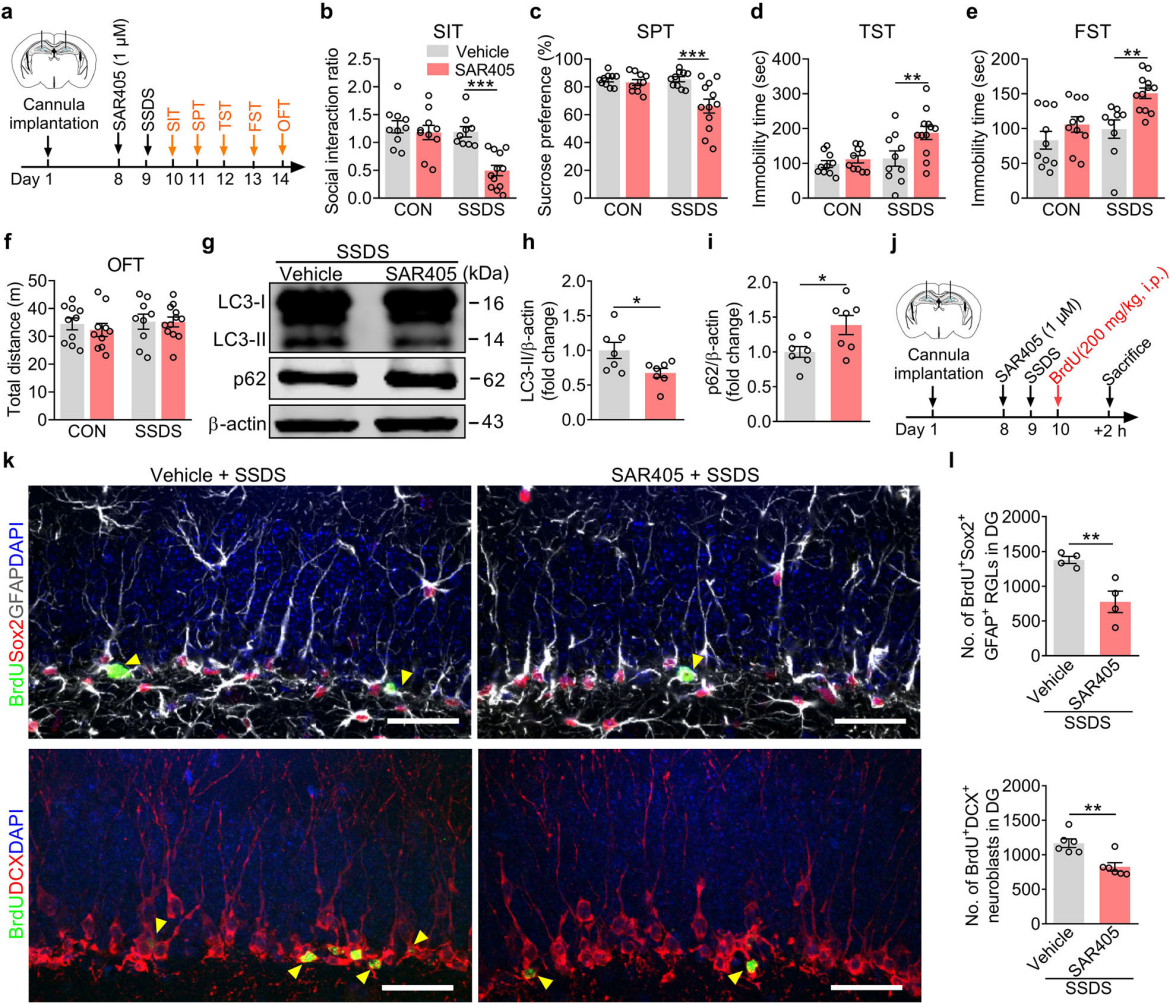
Selective VPS34 inhibitor SAR405 facilitates AHN impairment and the susceptibility of mice to stress. **a** Timeline of experiments. **b**–**f** Behavioral tests of SIT (**b**), SPT (**c**), TST (**d**), FST (**e**), and OFT (**f**) in control and SSDS-treated mice that microinjected with vehicle or SAR405 into DG (*n* = 10–12 mice per group). **g**–**i** Western blotting analysis (**g**) and quantifications of LC3-II (**h**) and p62 (**i**) protein expression in the DG (*n* = 7 mice per group). **j** Timeline of experiments. **k** Representative images of BrdU^+^Sox2^+^GFAP^+^ RGLs and BrdU^+^DCX^+^ neuroblasts in the DG. Yellow arrows indicate BrdU^+^ and marker^+^ cells. Scale bars, 30 μm. **l** Quantification showing the number of BrdU^+^Sox2^+^GFAP^+^ RGLs and BrdU^+^DCX^+^ neuroblasts in the DG (*n* = 4–6 mice per group). Data are presented as means ± SEM and analyzed by two-sided unpaired *t*-test (**h**, **i**, **l**) or two-way ANOVA followed by Bonferroni’s post hoc test (**b**–**f**). *P < 0.05, **P < 0.01, and ***P < 0.001.

Next, SAR405 (1 μM) was bilaterally microinjected into the DG to investigate the role of inhibition of the VPS34 complex in response to the stressful stimulus. It is well accepted that subthreshold social defeat stress (SSDS) allows the investigation of the pro-susceptibility effects of molecular manipulation^40^. SSDS was performed 24 h after SAR405 treatment (Fig. 6a), and behavioral tests showed that SAR405 increased the susceptibility of mice to SSDS as observed by decreased social interaction ratio in SIT and sucrose preference in SPT, while increased immobility time in TST and FST (Fig. 6b–e), without changes in locomotor activity (Fig. 6f). Meanwhile, SAR405 reduced the expression of LC3-II protein and increased the expression of p62 protein in the DG of SSDS-exposed mice (Fig. 6g–i). SSDS alone did not affect the expressions of NRBF2, LC3-II, and p62 proteins in the DG (Supplementary Fig. S9a–d). We also found that SAR405 significantly reduced the number of both BrdU^+^Sox2^+^GFAP^+^ RGLs and BrdU^+^DCX^+^ neuroblasts in SSDS-treated mice (Fig. 6j–l). Taken together, the results suggest that specific VPS34 inhibitor SAR405 contributes to stress susceptibility together with the decrease in adult neurogenesis in the DG.

### Cell-specific regulation of NRBF2 in aNSCs of the DG modulates depression-like behavior and adult neurogenesis of mice

**Fig. 7:**
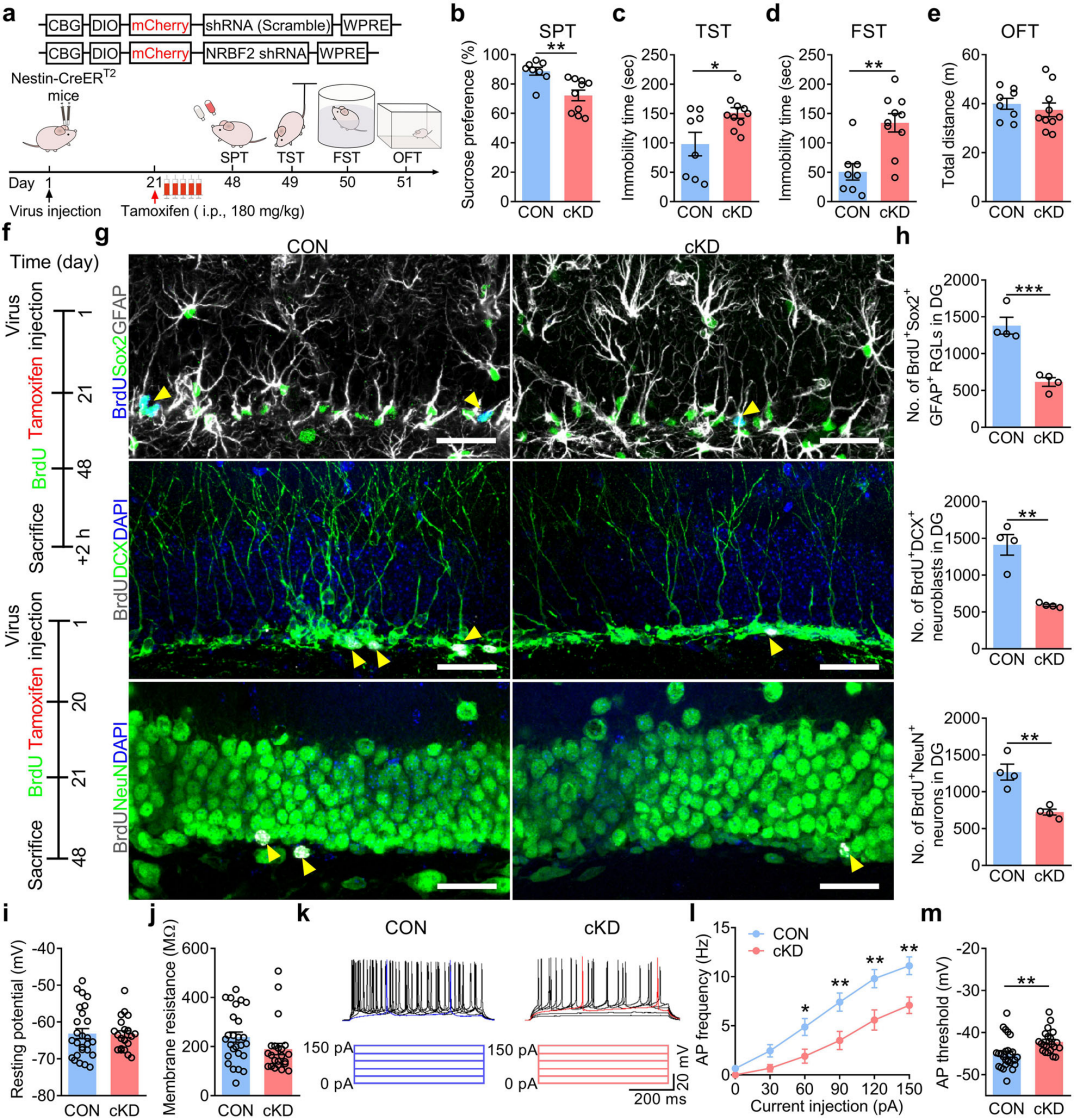
Conditional knockdown of NRBF2 in aNSCs induces AHN impairment and depression-like phenotype of mice. **a** Schematic diagram showing the experimental strategy of NRBF2 conditional knockdown (cKD) (upper). Timeline of experiments (bottom). **b**–**e** Behavioral tests of SPT (**b**), TST (**c**), FST (**d**), and OFT (**e**) in Nestin-CreER^T2^ mice treated with AAV-mCherry or AAV-sh*Nrbf2* (*n* = 8–10 mice per group). **f** Timeline of experiments. **g**, **h** Representative images (**g**) and quantification (**h**) of BrdU^+^Sox2^+^GFAP^+^ RGLs, BrdU^+^DCX^+^ neuroblasts, and BrdU^+^NeuN^+^ neurons in the DG (*n* = 4 mice per group). Yellow arrows indicate BrdU^+^ and marker^+^ cells. Scale bars, 30 μm. **i**, **j** Summarized data of the resting potential (**i**), and membrane resistance (**j**) of newborn neurons in the DG (*n* = 22–26 cells per group). **k** Representative spiking pattern of newborn neurons in response to current injection of 0–150 pA. **l**, **m** Quantification of the frequency of action potential elicited by current injection (**l**) and the threshold required for the first elicited action potential (**m**) (*n* = 22–26 cells per group). Data are presented as means ± SEM and analyzed by two-sided unpaired *t*-test (**b**–**e,** **h**–**j,** **m**) or two-way ANOVA followed by Bonferroni’s post hoc test (**l**). *P < 0.05, **P < 0.01, and ***P < 0.001.

To further confirm the role of aNSCs-specific NRBF2 in depression-like behavior, the Nestin-CreER^T2^ transgenic mice that expressed a tamoxifen-inducible Cre recombinase were used to specify the integration of exogenous gene into aNSCs^41^. Cre-dependent AAV vector that expressed mCherry-tagged NRBF2 short hairpin RNAs (AAV-sh*Nrbf2*) was microinjected into DG of Nestin-CreER^T2^ mice to obtain conditional knockdown (cKD) of NRBF2 in aNSCs (Fig. 7a). After i.p. injection of tamoxifen (180 mg/kg), the expression of mCherry was specifically targeted to aNSCs and their progeny within the DG (Supplementary Fig. S10a). It was observed that the expressions of NRBF2 and LC3-II protein were significantly reduced in the DG of NRBF2 cKD mice, implicating that the deficiency of NRBF2 inhibits autophagy in aNSCs (Supplementary Fig. S10b–d). Meanwhile, NRBF2 cKD mice displayed a depression-like phenotype, as indicated by the reduced sucrose preference in SPT and the increased immobility time in TST and FST (Fig. 7b–d), without changes in locomotor activity (Fig. 7e).

Then, quantitative assay exhibited a significant decrease in the number of BrdU^+^Sox2^+^GFAP^+^ RGLs, BrdU^+^DCX^+^ neuroblasts, and BrdU^+^NeuN^+^ neurons in the DG of NRBF2 cKD mice compared with that of control mice (Fig. 7f–h), indicating the defective proliferation of aNSCs and decreased production of newborn neurons in the DG of NRBF2 cKD mice. It has been demonstrated that suppressing the excitability of newborn neurons in the DG abolishes the antidepressant-like effects of fluoxetine^42^. We then investigated the effect of conditional knockdown of NRBF2 in aNSCs on the excitability of newborn neurons and found that there were no differences in resting membrane potential and membrane resistance of four-week-old newborn neurons between control and NRBF2 cKD mice (Fig. 7i, j). However, the frequency of action potential was reduced, and the threshold of action potential was significantly increased in the newborn neurons of hippocampal DG in NRBF2 cKD mice compared with that of control mice (Fig. 7k–m). The above results indicate that conditional knockdown of NRBF2 in aNSCs leads to depression-like behavior, the impairment of proliferation of aNSCs, and the functional and morphological integration of newborn neurons in the DG.

**Fig. 8:**
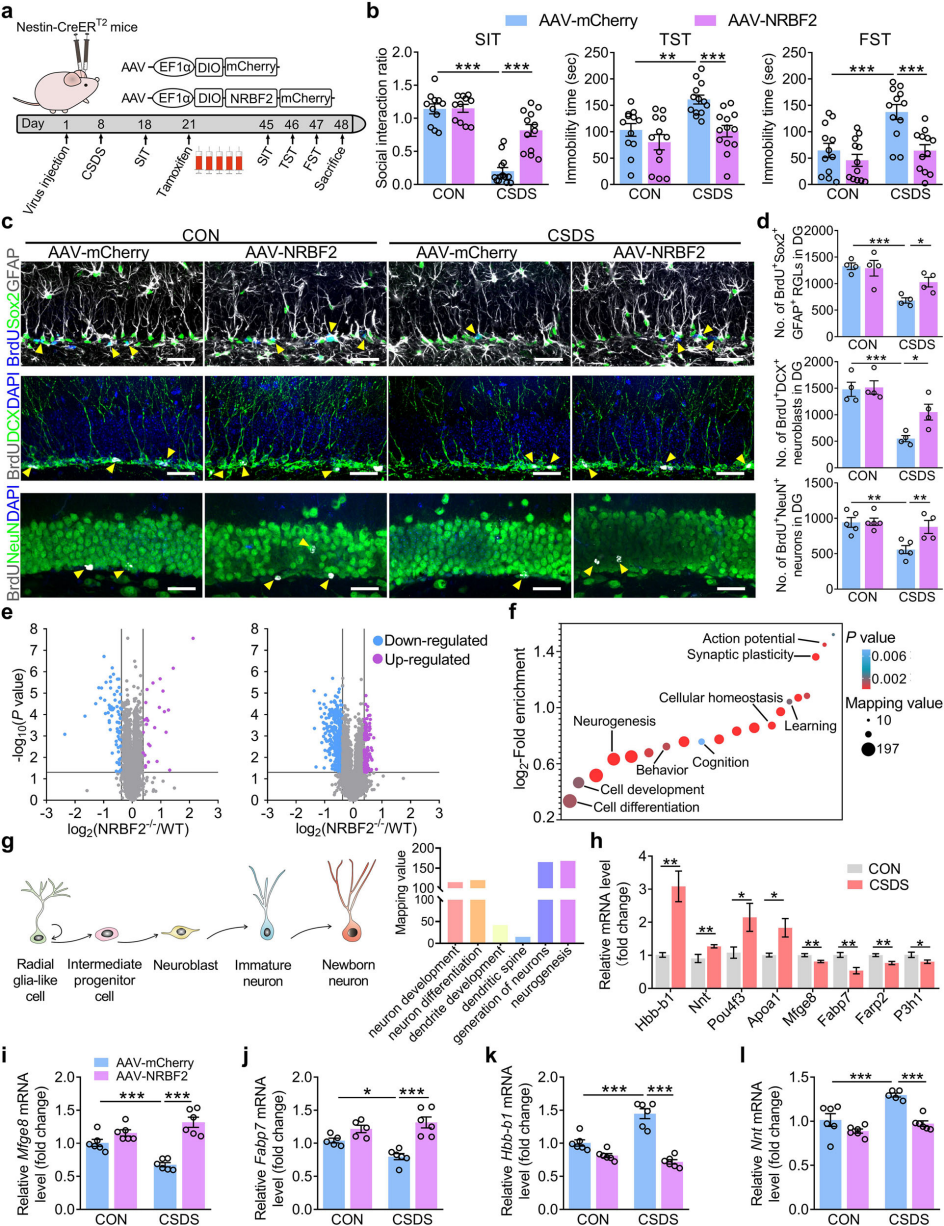
Overexpression of NRBF2 in aNSCs rescues disrupted neurogenesis-related protein network of DG induced by chronic stress. **a** Timeline of experiments. **b** Behavioral tests of SIT, TST, and FST on control and CSDS-exposed mice treated with AAV-mCherry or AAV-NRBF2 in DG aNSCs (*n* = 11–13 mice per group). **c**, **d** Representative images (**c**) and quantification (**d**) of BrdU^+^Sox2^+^GFAP^+^ RGLs, BrdU^+^DCX^+^ neuroblasts, and BrdU^+^NeuN^+^ neurons in the DG (*n* = 4–5 mice per group). Yellow arrows indicate BrdU^+^ and marker^+^ cells. Scale bars, 30 μm. **e** A total of 98/6791 proteins and 563/3374 phosphopeptides were statistically up- or downregulated in NRBF2^*−/−*^ mice (*n* = 3 mice per group). **f** The significant changes in expression or phosphorylation of proteins induced by NRBF2 deletion were most dominantly enriched in biological processes that involved neurogenesis, action potential, and synaptic plasticity. **g** A schematic diagram of each phase during adult hippocampal neurogenesis (left). Numbers in histograms are mapping values, i.e., the sum of phosphopeptides involved in neurogenesis (right). **h** CSDS upregulated the level of *Hbb-b1*, *Nnt*, *Pou4f3*, and *Apoa1* mRNA, but downregulated the level of *Mfge8*, *Fabp7*, *Farp2*, and *P3h1* mRNA in the DG (*n* = 5–8 mice per group). **i**–**l** The level of *Mfge8* (**i**), *Fabp7* (**j**), *Hbb-b1* (**k**), and *Nnt* (**l**) mRNA in the DG of control and CSDS-exposed mice treated with AAV-mCherry or AAV-NRBF2 (*n* = 5–6 mice per group). Data are presented as means ± SEM and analyzed by two-way ANOVA followed by Bonferroni’s post hoc test (**b**, **d**, **i**–**l**) or two-sided unpaired *t*-test (**h**), or Fisher’s exact test (**e**–**g**). *P < 0.05, **P < 0.01, and ***P < 0.001.

Furthermore, the number of GFP-LC3 puncta in the aNSCs of DG was significantly decreased in the CSDS-exposed mice compared with that in control mice (Supplementary Fig. S11a, b). Accordingly, we investigated whether aNSCs-specific overexpression of NRBF2 could ameliorate the chronic stress-induced depression-like behavior. Nestin-CreER^T2^ mice (7-week-old) were stereotaxically microinjected with AAV-EF1α-DIO-NRBF2 (AAV-NRBF2) or AAV-EF1α-DIO-mCherry (AAV-mCherry) into DG and then subjected to CSDS (Fig. 8a). After 3 weeks of injection, tamoxifen was given by i.p. injection to achieve specific overexpression of NRBF2 in the aNSCs and their progeny (Supplementary Fig. S12a). Nestin-CreER^T2^ mice treated with AAV-NRBF2 exhibited an increased expression of NRBF2 and LC3-II in the DG (Supplementary Fig. S12b–d), indicating that AAV-NRBF2 is effective for the induction of autophagy in aNSCs. Furthermore, the behavioral tests showed that conditional overexpression of NRBF2 in aNSCs significantly increased the social interaction ratio and decreased immobility time in TST and FST of CSDS-exposed mice compared with that of the control group (Fig. 8b), suggesting that conditional overexpression of NRBF2 in the DG aNSCs ameliorates CSDS-induced depression-like behavior. Meanwhile, AAV-NRBF2 restored the number of BrdU^+^Sox2^+^GFAP^+^ RGLs, BrdU^+^DCX^+^ neuroblasts, and BrdU^+^NeuN^+^ neurons in the DG of CSDS-exposed mice (Fig. 8c, d). These results provide direct in vivo evidence that specific restoration of NRBF2 levels in aNSCs ameliorates chronic stress-induced AHN impairment and depression-like behavior.

### Overexpression of NRBF2 in aNSCs rescues disrupted neurogenesis-related protein network of DG induced by chronic stress

To further explore the mechanisms underlying the AHN impairment induced by NRBF2 deficiency, the expression, and phosphorylation of proteins in the DG of NRBF2^*−/−*^ mice were screened. Proteomic and phosphoproteomic analysis revealed that a total of 98/6791 proteins and 563/3374 phosphopeptides were differentially regulated by NRBF2 deletion, including 34 proteins and 139 phosphopeptides enriched in the process of neurogenesis (Fig. 8e; Supplementary Fig. S13a–c). Among the differentially expressed proteins, 12 proteins (NNT, HBB-B1, APOA1, POU4F3, etc.) were upregulated, and 22 proteins (MFGE8, FABP7, FARP2, P3H1, etc.) were down-regulated. Bioinformatics analysis indicated that the dominant enrichments of differentially expressed proteins or phosphopeptides found in the biological process were involved in neurogenesis, cell development, cell morphogenesis, cellular homeostasis, action potential, and synaptic plasticity (Fig. 8f, g; Supplementary Fig. S13d–g).

Further analysis of qPCR revealed that chronic stress upregulated the mRNA levels of *Hbb-b1*, *Nnt*, *Pou4f3*, and *Apoa1* and downregulated the mRNA levels of milk fat globule-epidermal growth factor 8 (*Mfge8)*, *Fabp7*, *Farp2*, and *P3h1* in the DG (Fig. 8h), which was consistent with the proteomics analysis of NRBF2^*−/−*^ mice. MFGE8 is a stem cell-enriched autocrine factor that maintains the neural stem cell pool in adulthood by promoting RGLs quiescence^34^. Using absolute quantification via parallel reaction monitoring proteomics, the protein level of MFGE8 was found to be reduced in the DG of NRBF2^*−/−*^ mice (Supplementary Fig. S13h). The biochemical analysis also detected that the expression of MFGE8 protein was reduced in NRBF2^*−/−*^ mice, NRBF2-deficient aNSCs, and CSDS-exposed mice (Supplementary Fig. S13i–k). On the contrary, specific overexpression of NRBF2 in aNSCs increased the mRNA level of *Mfge8* and *Fabp7* but reduced the mRNA level of *Hbb-b1* and *Nnt* in the DG of CSDS-exposed mice (Fig. 8i–l). These results support the hypothesis that aNSCs-specific NRBF2 is a crucial mediator of neurogenesis-related protein networks in the DG.

## Discussion

In the present study, we discovered a previously unknown role for NRBF2 in the pathophysiology of depression via the regulation of AHN. It was found that chronic stress inhibited NRBF2-dependent autophagy in the DG. NRBF2 deficiency inhibited the activity of VPS34 complex and autophagic flux in aNSCs, which conferred the dysregulation of the neurogenesis-related protein network, resulting in the exhaustion of aNSCs pool and the defects in neuronal development of newborn neurons. On the other hand, conditional overexpression of NRBF2 in aNSCs ameliorated the depression-like behavior of CSDS-exposed mice or NRBF2^*−/−*^ mice (Supplementary Fig. S14). These findings provide substantial evidence that NRBF2 in the aNSCs orchestrates autophagic flux, AHN, and depression-like behavior under chronic stress.

Despite being studied for decades, whether AHN occurs in the adult human brain remains controversial^43,44^. Recently, the most compelling evidence demonstrates the neurogenic activity in the brain of donors up to the age of 92 years^45^, supporting the notion that human hippocampal neurogenesis persists throughout life^46,47^. It has been found that MDD patients display a decrease in the hippocampal volume and the number of adult hippocampal neural progenitors^4,48^, which can be reversed by antidepressant treatment^49^. It has been reported that chronic stress impairs the neurogenesis in the adult hippocampus, and AHN is necessary for the antidepressant-like effects^6,50^. However, the process of AHN impairment remains undefined under chronic stress. In our study, we revealed that chronic stress led to the exhaustion of the aNSCs pool and impaired newborn neuron development in the DG. Furthermore, restoring the expression of NRBF2 in aNSCs rescued chronic stress-induced AHN impairment and depression-like behavior while suppressing AHN was sufficient to abolish the antidepressant-like effect of NRBF2 overexpression. Collectively, our results suggest that AHN is sufficient and necessary for the antidepressant action of NRBF2 overexpression, pointing to autophagy deficiency as the mechanism underlying chronic stress-induced AHN impairment.

Autophagy is generally regarded as an adaptive cellular response to stress through the removal of dysfunctional organelles and protein aggregates^51^, and autophagy dysfunction has been implicated in the pathogenesis of MDD. For example, autophagy is inhibited in CUS- and CORT-induced mouse models of depression, and both autophagy deficiency and depression-like behavior are rescued by antidepressants and autophagy inducers^24,52^. Here, we found that chronic stress inhibited NRBF2-mediated autophagy, which was indicated by the reduced number of autophagic vacuoles and LC3 puncta in the DG. The autophagic receptor p62 is a multifunctional protein, which is crucial for the formation of autophagosomes^53,54^. Our results showed that the mRNA and protein levels of p62 were reduced in the DG of CSDS-exposed mice, implying that the reduced expression of p62 resulted from its downregulated transcriptional level. Furthermore, a previous study demonstrates that p62 knockout mice displayed the depression-like phenotype^55^. Even though autophagy inhibition is usually accompanied by an increased level of p62 protein^38,56^, the discrepancy is likely due to the context-dependent regulation of autophagy in a stress-specific manner. Therefore, these results suggest that the reduced transcriptional level of p62 may downregulate its protein expression and then aggravate autophagic inhibition. However, mechanistic insights into the transcriptional mechanism of p62 need to be further investigated. Schaffner et al. have found that conditional knockout of FOXOs in aNSCs impair autophagic flux and results in altered dendritic morphology and increased spine density in newborn neurons^37^. However, it remains unclear how autophagy deficiency couples to the impairment of AHN under chronic stress. Our results revealed that the deficiency of NRBF2-dependent autophagy in the DG aNSCs impaired the AHN and maturation of newborn neurons, which could facilitate a depression-like phenotype. Although we cannot completely rule out that the deficits in autophagosomes induced by NRBF2 knockout are specifical in DG, aNSCs-specific restoration of NRBF2-dependent autophagy in the DG improved depression-like behavior of NRBF2^*−/−*^ mice or CSDS-exposed mice via stimulation of AHN. These results suggest that NRBF2-dependent autophagy in aNSCs is sufficient to restore the AHN impairment and depression-like behavior.

Consistent with the results of single-cell transcriptomes^28,29^, NRBF2 is expressed in the adult hippocampal neurogenic lineage. ATG14L-containing PtdIns3K complex is believed to specifically regulate autophagosome biogenesis^15^. Increasing evidence has shown that NRBF2 positively regulates autophagy, such as involvement in both autophagy initiation and maturation^18,19,21,57–59^, while one study reported that *Nrbf2* siRNA resulted in a significantly reduced protein level of p62 protein, but not LC3-II, in the retinal pigment epithelial-1 (RPE-1) cells^60^, implying that NRBF2 regulates autophagy in a tissue- or cell type-specific manner. Our experiment observed the reduced activity of the ATG14L-linked VPS34 complex in NRBF2-deficient aNSCs. Furthermore, the deficiency of NRBF2 impaired autophagosome formation and autophagic flux in aNSCs, suggesting a positive role of NRBF2 in modulating autophagosome formation in aNSCs. Pharmacological inhibition of VPS34 activity suppressed NRBF2-dependent autophagy and AHN in the DG, which facilitated the susceptibility of mice to stress, while specific overexpression of NRBF2 in DG aNSCs ameliorated CSDS-induced AHN impairment and depression-like behavior via inducing autophagy. Fip200 is involved in the autophagy initiation, while Atg5, Atg16L1, and Atg7 are involved in autophagosome elongation^61,62^. Wang et al. showed that Fip200 deletion, but not Atg5, Atg16L1, or Atg7, impaired the maintenance and differentiation of postnatal neural stem cells^63^, suggesting that not all autophagy-impairment leads to reduced neurogenesis. Pharmacological inhibition or genetic mutation of PI3K-III complex components has been shown to decrease the proliferation and differentiation of NSCs^27,64,65^, which is consistent with our observation. These findings emphasize the positive role of autophagy initiation on neurogenesis. Collectively, these results indicate that the ATG14L-containing PtdIns3K complex contributes to NRBF2-dependent autophagy in aNSCs and then restores the defective AHN in chronic stress-induced depression.

Maintaining a homeostatic protein network throughout life is essential for the cellular function of aNSCs^66^. Disruptions in the proteome, and the autophagy-lysosome pathway, in particular, have been associated with defective adult neurogenesis in mammals^25,67^. A recent study identifies that FOXO3 directly regulates the autophagy network to functionally maintain proteostasis in aNSCs^68^. Proteomic analyses from rodent hippocampus suggest that neurogenesis-related protein networks may be a crucial target of classical antidepressant treatment^69,70^. Our proteomic and phosphoproteomics data revealed a series of NRBF2-specific molecular signatures, including NNT, HBB-B1, MFGE8, and FABP7, all of which have been demonstrated in the regulation of neurogenesis^34,71–74^. NNT, HBB-B1, and FABP7 have additionally been implicated in mood disorders^75–77^, while NNT and MFGE8 are associated with the autophagic pathway^74,78^. In our study, conditional overexpression of NRBF2 in DG aNSCs rescued the disrupted expression of the above genes in CSDS-exposed mice, suggesting that NRBF2 is critical for maintaining the neurogenesis-related protein network in the DG. Given that NRBF2 originally functioned as a possible gene activator protein^79^, future study is needed to investigate whether the transcriptional mechanisms of NRBF2 were involved in this process. Beyond these housekeeping functions, autophagy has specific roles in neuronal excitability and synaptic plasticity^80,81^. For example, one study identified a novel role for autophagy in modulating synaptic transmission by enhancing protein kinase A (PKA) activity at the synapse and found that autophagy regulates synaptic function through PKA-dependent phosphorylation of postsynaptic scaffold proteins independently of their degradation^81^, indicating that neurons also utilize components of the autophagy machinery to regulate signaling involved in synaptic function without the need for degradative protein turnover. In our study, we found that the deficiency of NRBF2-dependent autophagy disrupted the neurogenesis-related proteins according to the proteomic data and disturbed the structural development and synaptic plasticity based on the phosphoproteomic data. Thus, we speculated that the deficiency of NRBF2-dependent autophagy might impair the neurogenesis and synaptic function through the degradation of protein turnover and autophagy machinery-mediated signaling, which need to be further clarified.

Quiescence has also been suggested to be essential for establishing the aNSC pool. However, the abnormal activation of quiescent RGLs leads to their depletion in adult SGZ^82^. Quiescent NSCs in aged brains display defective autophagy–lysosome pathways and reduced ability to maintain homeostasis^67^. Proteomics and biochemical analyses identify that NRBF2 is essential for preserving the normal expression of MFGE8, which is critical for preventing the exhaustion of aNSCs pool^34^, indicating that MFGE8 is a previously unidentified signaling pathway in NRBF2-regulated homeostasis of aNSCs pool. These results further indicate that autophagy deficiency induced by chronic stress disrupts aNSCs homeostasis and then leads to the exhaustion of aNSCs pool and AHN-mediated depression-like behavior in mice. In summary, our results presented here illustrate a molecular and cellular basis for a better understanding of how chronic stress disrupts autophagy, especially in aNSCs, and eventually exerts behavioral effects in an AHN-dependent manner. The deficiency of NRBF2-dependent autophagy in the DG aNSCs led to AHN-mediated depression-like behavior by inhibition of ATG14L-containing PtdIns3K complex and impairment of autophagic flux. Our results also identify that targeting the cellular mechanisms underlying the proneurogenic effect of NRBF2 could be a potential strategy for the treatment of MDD.

## Materials and methods

### Animals

NRBF2^*−/−*^ mice were generous gifts from Prof. Zhenyu Yue (Icahn School of Medicine at Mount Sinai, NY, USA). Nestin-CreER^T2^ mice were obtained from Jackson Laboratory (Jackson Lab, 003771; RRID: IMSR_JAX:003771, ME, USA). All mice in the study were backcrossed to the C57BL/6J background. NRBF2 heterozygous mutant mice were outbred with C57BL/6 J mice (Charles RIVER, Beijing, China) and interbred to obtain NRBF2^*−/−*^ mice. Male mice between 6 to 10 weeks of age were selected throughout the study. All mice were maintained under standard laboratory conditions, with 12 h alternating light/dark cycle and constant temperature (22 ± 2 °C) with free access to water and food. WT mice or Nestin-CreER^T2^ mice were used for CSDS models. For immunohistology, brain sections from WT mice, NRBF2^*−/−*^ mice, or Nestin-CreER^T2^ mice were obtained for immunostaining. For knockout and conditional rescue analyses, mice used in different groups were housed with littermates: WT mice and NRBF2^*−/−*^ mice. WT mice, NRBF2^*−/−*^ mice, or Nestin-CreER^T2^ mice were used for virus injection. All animal experiments were approved by the Animal Care and Use Committee of Huazhong University of Science and Technology and were manipulated according to ARRIVE 2.0 guideline^83^.

### Chronic social defeat stress

CSDS was performed as described previously^84^. Briefly, 8-week-old male mice (intruders) were introduced into the home cage of a novel CD1 aggressor (resident) for 5–10 min once per day. After the physical defeat, C57BL/6J and CD1 mice were separated by a perforated plexiglass divider to maintain sensory contact for 24 h, which were repeated daily for 10 consecutive days with a different CD1 aggressor each day and tested 24 h later for SIT. Control mice were housed in pairs and never in physical or sensory contact with CD1 aggressor mice.

### Chronic unpredictable stress

CUS was performed as previously described^85^. Eight-week-old C57BL/6J male mice were housed singly in cages and subjected to random, unpredictable mild stressors, including day/night light cycle, 45° tilted cage (12 h), restraint stress (1 h), foot-shock (0.5 mA, 5 times, 30 s intervals), cold environment (1 h at 4 °C), cold swimming, low-intensity stroboscopic illumination (10 Hz during 12 h in the dark), wet bedding (250 mL water added into the cage overnight) and no bedding (overnight), food and water deprivation (overnight). All stressors were randomly interspersed throughout the stress period for 5 weeks. Control mice were housed in groups of five, and they were never subjected to stress.

### CORT-induced mouse model of depression

As previously described^86^, eight-week-old C57BL/6J male mice were individually housed and administrated with corticosterone (MCE, NJ, USA) (35 μg/mL, equivalent 5 mg/kg/d), which dissolved in 0.45% (wt/vol) hydroxypropyl-β-cyclodextrin (Sigma-Aldrich, MO, USA) or vehicle (0.45% hydroxypropyl-β-cyclodextrin) in drinking water for 30 consecutive days.

### Subthreshold social defeat stress

SSDS paradigm was performed as previously described^84^. The 8-week-old male mouse was introduced to a novel CD1 aggressor for three consecutive 5-min defeat stress, with 15-min intervals between exposures. Following the last defeat session, all mice were housed singly in cages with *ad libitum* access to food and water. Behavioral tests were carried out 24 h later and recorded using ANY-maze software (Stoelting Co, IL, USA).

### Social interaction test

SIT was performed as previously described^87^. All experimental C57BL/6J mice were habituated in the room ~1 h before the test. Mice were placed in an open field (42 × 42 × 42 cm^3^), which included an interaction zone and two opposing corner zones. Their movements were monitored and recorded using ANY-maze software for 2.5 min in the absence (“no target” phase) and presence (“target” phase) of a new CD1 mouse, respectively. Social avoidance behavior was calculated as the total time spent by the C57BL/6J mouse in the interaction zone in the presence vs. absence of the target. Animals were considered CSDS-exposed mice when this ratio was <1.

### Sucrose preference test

SPT was assayed according to the previously described method with some modifications^88^. Mice were habituated to two identical 50 mL bottles containing water for 48 h. After each 12 h, the location of the bottles was interchanged to eliminate position preference. After habituation, mice were given access to a two-bottles filled with water or 1% sucrose solution. Sucrose or water consumption was measured by a 24-h test, and the location of two bottles was interchanged to ensure that mice did not display side preference. Sucrose preference was calculated according to the following formula: sucrose preference (%) = volume of sucrose consumed/total volume of sucrose and water consumed) × 100%.

### Tail suspension test

Mice were habituated in the test room ~1 h before each trial. In brief, mice were individually suspended 20 cm above the floor with the tape placed 1 cm from the tip of the tail. Plastic tubes were put in the tails to ensure mice could neither climb nor hang on to their tail. All mice were recorded for 6 min, and the immobility time was calculated over the 6-min observation period.

### Forced swim test

Mice were placed individually into a transparent glass cylinder (25 cm in height, 10 cm in diameter) filled with a depth of 18 cm water (25 ± 1 °C). All mice were forced to swim for 6 min, and the immobility time during the final 4-min interval of the test was recorded using ANY-maze software. The water was exchanged after each session to avoid any influence on the next mouse.

### Locomotor activity

Locomotor behavior was performed in the OFT to evaluate the motor ability of animals after injection with viruses or drugs. In brief, mice were habituated in the test room for ~1 h before each test. Animals were placed in an open field (42 × 42 × 42 cm^3^). The total distance traveled in the open field was monitored and recorded using ANY-maze software for 10 min.

### Transmission electron microscopy

Transmission electron microscopy was based on a previously described procedure^38^. Control and CSDS-exposed mice were anesthetized with sodium pentobarbital (40 mg/kg, i.p.) and perfused with 2.5% glutaraldehyde, then the brains were removed and immersed in the same fixative overnight at 4 °C. After washing with 0.1 M phosphate buffer, the tissues were post-fixed with 1% osmium tetroxide for 5 min at room temperature. The brain sections were then dehydrated in graded ethanol concentrations and embedded in epoxy resin according to standard protocols. Ultrathin sections (400 nm) in the DG region were post-stained with uranyl acetate and lead citrate and finally viewed with an FEI Tecnai G2 12 transmission electron microscope (FEI Company, Hillsboro, Oregon) operated at 80 kV.

### Stereotaxic injections and cannulas implantation

Mice were anesthetized with sodium pentobarbital (40 mg/kg, i.p.) and then fixed on a stereotactic apparatus. For stereotaxic viral injections. The virus of RV-CAG-RFP (titers: 5.00 × 10^8^ VG/mL), pLV-CMV-MSC-3Flag (titers: 4.34 × 10^8^ VG/mL), pLV-Nrbf2-IRES-ZsGreen1 (titers: 5.56 × 10^8^ VG/mL), rLV-GFP-LC3 (titers: 5.00 × 10^8^ VG/mL), rLV-RFP-LC3 (titers: 5.00 × 10^8^ VG/mL) were bilaterally injected into the DG (AP = −2.00 mm, ML = ± 1.50 mm, DV = −2.15 mm; relative to bregma) with a volume of 1 μL, respectively. AAV-EF1α-DIO-Nrbf2-mCherry-WPRE (titers: 3.10 × 10^12^ VG/mL), AAV-EF1α-DIO-mCherry-WPRE (titers: 1.40 × 10^12^ VG/mL), AAV-CBG-DIO-mCherry-miR30shRNA (NRBF2)-WPRE (titers: 1.43 × 10^13^ VG/mL), and AAV-mCherry-miR30shRNA (NC)-WPRE (titers: 1.55 × 10^13^ VG/mL) were bilaterally injected into the DG with a volume of 0.3 μL, respectively. For overexpression of NRBF2 in aNSCs of NRBF2^*−/−*^ mice, a viral cocktail (10:3) of RV-EF1α-Cre (titers: 7.77 × 10^7^ VG/mL) and AAV-EF1α-DIO-Nrbf2-mCherry or AAV-EF1α-DIO-mCherry were bilaterally injected into the DG with a volume of 1.3 μL. Viruses were injected at a rate of 0.05 μL/min through an automatic microinjection system (World Precision Instruments, USA). After completion of the injection, the nanoliter injector stayed for an additional 10 min before withdrawal. The skin was sutured and then sterilized with iodophors. Mice were recovered for 2–4 weeks before subsequent manipulations. The site of virus infection was verified by examining virus-expressed fluorescent protein on coronal brain slices.

For intra-DG microinjection, 22-gauge stainless steel guide cannulas (RWD Life Science, Shenzhen, China) were implanted bilaterally above the DG (AP = −2.00 mm, ML = ± 1.50 mm, DV = −2.10 mm; relative to bregma). After the implant surgery, mice were recovered for 7 days before further experiments. SAR405 (Apex Bio, TX, USA) was dissolved in 0.1% DMSO. A volume of 1 μL SAR405 (1 μM) or vehicle (0.1% DMSO) was microinjected into the DG, respectively.

### RNA isolation and qPCR

Total RNA was isolated from the brain tissues by using Trizol (Invitrogen, CA, USA) according to the manufacturer’s protocol. The first-strand cDNA was prepared from RNA (1 μg) by using the PrimeScript RT Master Mix (Takara, Shiga, Japan). The qPCR systems (20 µL reaction volume) included 2× SYBR^®^ Premix Ex Taq™ II (Takara, Shiga, Japan) 10 µL, forward primer (10 µM) 0.4 µL, reverse primer (10 µM) 0.4 µL, RNase-free H_2_O 8.2 µL, and pre-amplified cDNA 0.8 µL. Reactions were performed in 96-well plates in a Bio-Rad Real-Time PCR System (CFX96, Bio-Rad, USA). qPCR amplification consisted of an initial denaturation for 30 s at 94 °C, followed by 40 cycles at 95 °C for 30 s and 60 °C for 30 s. Analysis of gene expression was performed using the ΔΔ^Ct^ method, and relative fold-change in target gene expression was normalized to housekeeping gene glyceraldehyde-3-phosphate dehydrogenase (*Gapdh*). Gene expression analyses were expressed as RNA levels relative to controls. All primer sequences were listed in Supplementary Table S1.

### Drug administration

To induce recombination, 8–10-week-old mice received tamoxifen (Sigma-Aldrich, MO, USA) daily for 5 days (180 mg/kg, i.p., 30 mg/mL in 10% EtOH/Corn oil) based on a published procedure^27^. Temozolomide (MCE, NJ, USA) was dissolved in DMSO and diluted to 2.5 mg/mL in sterile saline. Mice were i.p. injected with vehicle or temozolomide (25 mg/kg) for three consecutive days per week and lasted 4 weeks^89^. For analysis of cell proliferation in the DG, adult mice were injected with BrdU (200 mg/kg, i.p.) (Sigma-Aldrich, MO, USA) and analyzed the number of BrdU^+^ cells in the DG 2 h after injection. For analysis of cell survival in the DG, adult mice were injected with BrdU (50 mg/kg, i.p.) twice per day for 2 days and analyzed 4 weeks after the last BrdU injection.

### Tissue preparation and immunohistochemistry

Mice were anesthetized with sodium pentobarbital (40 mg/kg, i.p.) and then transcranial perfused with 0.9% saline followed by 4% paraformaldehyde (PFA). Brains were removed, post-fixed overnights in 4% PFA, and then equilibrated in 30% sucrose. Frozen coronal sections (40 μm thick) containing the total DG were obtained using a freezing microtome (CM1900, Leica, Wentzler, Germany) and stored in 6-well plates filled with cryoprotectant solution (Glycerol, ethylene glycol, and 0.1 M phosphate buffer, 1:1:2 by volume, pH 7.4) at −20 °C. The sections were blocked with PBS containing 2% donkey serum, and 0.3% Triton X-100 for 1 h at room temperature, followed by incubation with primary antibody overnight at 4 °C. Sections were then incubated with secondary antibodies for 2 h at room temperature. All sections were counterstained with a nuclear counter stain, DAPI (Sigma-Aldrich, MO, USA). Primary antibodies and secondary antibodies were provided in Supplementary Table S2. For BrdU staining, sections were pretreated with 1 N HCl for 10 min and then 2 N HCl for 10 min at room temperature and an additional 20 min at 37 °C. Before blocking and incubation, sections were then rinsed in 0.1 M boric acid (pH 8.5). The following staining steps were carried out as described above. Confocal images were taken with a laser confocal scanning microscope (FV1000, Olympus, Tokyo, Japan).

### Image acquisition, processing, and analysis

For quantification of cells expressing stage-specific markers, 1 in 12 serial sections starting at the beginning of the hippocampus (relative to bregma, −1.5 mm) to the end of the hippocampus (relative to bregma, −3.5 mm) were examined. Quantifications of BrdU^+^ cells, BrdU^+^Sox2^+^GFAP^+^ cells, BrdU^+^Tbr2^+^ cells, BrdU^+^DCX^+^ cells, and BrdU^+^NeuN^+^ cells in the granular layer were performed using FV1000 confocal microscope with FV10-ASW software in six sections containing the DG from at least three different animals. The exact value of *n* was described in the figure legends.

For analysis of dendritic branching, the retrovirus-infected neurons in 100-mm-thick floating brain sections were imaged on a confocal microscope (FV1200, Olympus, Tokyo, Japan) with a 20× per objective. Z stacks of the dendrites of retrovirus-infected neurons were captured at 1.5-mm intervals and analyzed by ImageJ software (NIH, MD, USA). Data were extracted for Sholl analysis and total dendritic length from each retrovirus-infected neuron. At least 8–15 cells per group from at least 3 different animals were analyzed. The exact value of *n* was described in the figure legends.

For analysis of the dendritic spine, 40-mm floating brain sections containing retrovirus-infected neurons were used for analysis. Dendritic segments were imaged on an Olympus confocal with a 100× per oil objective and zoom of 2.5. The number of dendritic spines was counted with three-dimensional (3D) tracks using Imaris software (Bitplane, Oxford, UK). Imaris classified the spine as thin, mushroom, or stubby based on specific parameters from the previous study^90^. The result of dendritic spine density was normalized as the number of spines per 10 mm length of dendrite. At least ten dendritic segments per group from at least three different animals were analyzed. The exact value of *n* was described in the figure legends.

### Electrophysiological recording

Mice were anesthetized with sodium pentobarbital (40 mg/kg, i.p.) and then perfused with an ice-cold oxygenated cutting solution containing (mM): sucrose 209, ascorbic acid 11.6, sodium pyruvate 3.1, MgSO_4_ 4.9, NaHCO_3_ 26.2, NaH_2_PO_4_ 1.0, glucose 20, pH 7.4, osmolarity 290–310 mOsm. Brain slices were incubated in artificial cerebrospinal fluid (ACSF) containing (mM): NaCl 119, MgSO_4_ 1.3, KCl 4.3, NaHCO_3_ 26.2, NaH_2_PO_4_ 1.0, glucose 10, CaCl_2_ 2.9, pH 7.4, osmolarity 290–310 mOsm for recovery at 28 °C for 1 h. DG-containing slice was then transferred into the recording chamber with continuous perfusion (2 mL/min) of ACSF at room temperature. The resistance of patch pipettes was 3–6 MΩ. For current–clamp recording, action potentials from mCherry^+^ newborn neurons in DG were recorded by the injection of 0 to 150 pA current at a holding potential of −70 mV. The internal solution for action potentials recording contained (mM): K-gluconate 97, KCl 38, EGTA 0.35, HEPES 20, NaCl 6, Phosphocreatine-Na 7, Mg-ATP 4, Na-GTP 0.35, pH 7.2, 280–300 mOsm. All recordings were performed under an upright Olympus microscope (BX51WIF, Olympus, Tokyo, Japan). Signals were obtained through a MultiClamp 700B amplifier (Molecular Devices, CA, USA) and acquired with pCLAMP10 software (Axon Instruments, Molecular Devices, CA, USA). Series resistance was monitored during recording, and data were discarded for those altered by > 20%^91^. Data were analyzed by the Mini Analysis Program (Synaptosoft, GA, USA).

### Isolation, culture, and in vitro analyses of aNSCs

aNSCs used in this study were isolated from the DG of 8-week-old male C57BL/6J mice based on published methods^27^. aNSCs were maintained in proliferation medium (DMEM/F-12 medium containing 20 ng/mL basic fibroblast growth factor (PeproTech, NJ, USA), 20 ng/mL epidermal growth factor (PeproTech, NJ, USA), 1% antibiotic-antimycotic (GIBCO, NJ, USA), 1% B-27 (GIBCO, NJ, USA), and 2 mM l-glutamine (GIBCO, NJ, USA) in a 5% CO_2_ incubator at 37 °C. Half of the medium was replaced every 2 days. LV-sh*Nrbf2* was transfected under proliferation conditions for 2 days before the initiation of proliferation or differentiation assays. Lentiviruses expressing GFP or blue fluorescent protein were used as control. The efficacy of infection was confirmed by using western blotting and immunocytochemistry assays.

Proliferation and differentiation analyses were performed as described previously^27^. To study cell proliferation, aNSCs were dissociated with trypsin (GIBCO, MA, USA) and plated on poly-l-ornithine/laminin-coated slides at a density of 50,000 cells/well in a proliferation medium. BrdU (5 μM) was added into the culture medium for 8 h. aNSCs were then washed with PBS and fixed with 4% PFA for 30 min at room temperature. For the differentiation assay, aNSCs were cultured in a differentiation medium (DMEM/F12 containing forskolin (5 Μm; Sigma-Aldrich, MO, USA), retinoic acid (1 μM; Sigma-Aldrich, MO, USA), 1% antibiotic-antimycotic, 1% B-27, and 2 mM l-glutamine) for 4 days, then fixed with 4% PFA for 30 min, and then subjected to immunocytochemistry analysis.

### Cell viability analysis

Cell viability was assessed using a Cell Counting Kit-8 (CCK-8) assay (Apex Bio, TX, USA) following the manufacturer^92^. Briefly, control or NRBF2-deficient aNSCs were plated in a poly-L-ornithine/laminin-coated 96-well plate at a density of 5000 cells/well and treated with vehicle or bafilomycin A1 (20 nM) for 12 h. Then, CCK-8 was added, and the absorbance at 450 nm was detected using a microplate reader (Tecan, Männedorf, Switzerland).

### Western blotting assay

Fresh cells and brain tissues were sonicated in RIPA lysis buffer (50 mM Tris, 150 mM NaCl, 1% Triton X-100, 1% sodium deoxycholate, 0.1% SDS, pH 7.4) containing protease and phosphatase inhibitors. Samples were then centrifuged at 12,000 × *g* for 20 min at 4 °C, and the supernatant was collected and quantified with the bicinchoninic acid assay (Beyotime, Nantong, China). All the protein samples were denatured for 10 min at 95 °C in the loading buffer. Proteins (30 μg) were separated through SDS-PAGE and then incubated overnight at 4 °C with primary antibodies (Supplementary Table S2). β-actin (Santa Cruz, CA, USA) was used as the loading control. All bands were visualized by an Odyssey Imaging System (LI-COR Biosciences, NE, USA). The original blots for representative images were provided in Supplementary Figs. S15 and S16.

### Immunoprecipitation

Cell lysates were lysed in Nonidet P-40 cell lysis buffer (Beyotime, Nantong, China) containing protease inhibitors cocktail. Then, immunoprecipitation was performed using the indicated antibodies. Generally, an anti-ATG14L antibody (5 µg; MBL, Tokyo, Japan) was added to 500 µg of cell lysate and incubated at 4 °C overnight. After the addition of protein A/G-agarose beads (Santa Cruz, TX, USA), incubation was continued for 3–4 h. The immunocomplexes were deactivated by boiling at 95 °C for 5 min and analyzed by western blotting assay. The clean-blot immunoprecipitation detection kit (Thermo Fisher Scientific, MA, USA) was used to avoid interference from the immunoprecipitation primary antibody. The clean-blot™ immunoprecipitation detection kit eliminated detection interference from both heavy-chain (50 kDa) and light-chain (25 kDa) IgG fragments of antibodies used for the initial immunoprecipitation assay.

### PtdIns3P ELISA assay

Endogenous VPS34 protein was immunoprecipitated from aNSCs treated with LV-GFP or LV-sh*Nrbf2* and subjected to VPS34 activity assay using PtdIns3P ELISA Kit (Echelon, UT, USA) following the manufacturer. Briefly, 40 mL kinase reaction buffer (20 mM Tris-HCl at pH 8.0, 2 mM EDTA, 10 mM MnCl_2_, and 100 μM ATP), 4 μL of 500 mM phosphatidylinositol substrate, 8.5 μL ddH_2_O were added to the immunocomplex and incubated at 37 °C for 1 h. The kinase reaction was terminated by adding 5 μL of 100 mM EDTA. The quenched reaction mixture and PtdIns3P detector buffer were added together to the PtdIns3P-coated microplate for competitive binding to the PtdIns3P detector protein. The amount of PtdIns3P detector protein bound to the plate was determined through colorimetric detection of absorbance at 450 nm. The concentration of PtdIns3P in the reaction mixture was calculated as the reversed amount of PtdIns3P detector protein bound to the plate.

### Proteomic and phosphoproteomic analysis

Mice were executed by cervical dislocation, and brains were quickly removed. DG tissues were isolated on ice, grinded in liquid nitrogen, and then dissociated by sonication in a buffer containing 8 M urea and 1% protease inhibitor cocktail. Samples were collected after centrifugation at 4 °C, 12,000 × *g* for 10 min. Protein concentrations were determined using BCA assays. For mass spectrometry, samples were then diluted by triethyl ammonium bicarbonate (Sigma-Aldrich, CA, USA), and trypsin was added (1:50, enzyme to protein) for digestion overnight. Protein solutions were revivified with dithiothreitol (5 mM) for 30 min at 56 °C and alkylated with iodoacetamide (11 mM) for 15 min at room temperature in darkness. After the trypsin digestion, peptides were desalted by Strata X C18 SPE column, vacuum-dried, reconstituted in 0.5 M TEAB, labeled using a TMT kit (Thermo Fisher Scientific, MA, USA), and then fractionated by high pH reverse-phase HPLC using Agilent 300Extend C18 column (5 mm particles, 4.6 mm ID, 250 mm length). Phosphopeptides were enriched using immobilized metal affinity chromatography (IMAC) microspheres. Mass spectrometry data were collected using a Q Exactive^TM^ HF-X Plus Hybrid Quadrupole-Orbitrap mass spectrometer coupled with EASY-nLC 1200 liquid chromatography pump (Thermo Fisher Scientific, MA, USA).

The obtained mass spectral data were searched against the SwissProt Mouse database. Expression of each protein or phosphopeptide in all six samples was horizontally normalized and converted into log_2_ values. The mean values were calculated as Mm (WT group) or Mt (NRBF2^*−/−*^ group). Two-tailed *t*-tests were used to evaluate the differences between WT and NRBF2^*−/−*^ groups. For each protein or phosphopeptide, *P* < 0.05 and Mt/Mm > 1.3 were considered as significantly upregulated, while *P* < 0.05 and Mt/Mm < 1/1.3 as significantly downregulated. Proteomic and phosphoproteomic data were combined in bioinformatics analysis. For Gene Ontology (GO) annotation, the UniProt-GOA database was converted to identified protein ID to UniProt IDs and then mapped to GO IDs. If some identified proteins were not annotated by the UniProt-GOA database, the InterProScan software was designed to annotate their GO function based on the method for alignment of the protein sequence. Then, proteins were classified by GO annotation based on biological processes. Two-tailed Fisher’s exact test was employed to evaluate the enrichment of the differentially expressed protein against all identified proteins. The GO with a corrected *P* value < 0.05 is considered significant.

### Statistical analysis

All data were presented as the means ± SEM and performed in GraphPad Prism 8.0 (GraphPad Software, CA, USA). Dots in the figure represent individual mice, cells, dendritic segments, or independent experiments. Sample sizes can be found within the figure legend. Data distribution was assumed to be normal, but this was not formally tested. Comparison between the two groups was evaluated by unpaired Student’s *t*-test. Multiple comparisons were carried out using one-way or two-way analysis of variance followed by Tukey’s post hoc test or Bonferroni’s post hoc test. Sholl analysis was carried out using repeated measures ANOVA by using GraphPad software. *P* < 0.05 were considered as statistical significance, and exact statistic information and *P* value were provided in Supplementary Table S3.

### Supplementary information

Supplementary Information

## Data Availability

The proteomic and phosphoproteomic data reported in this paper are available at ProteomeXchange Consortium: PXD034382. Any additional information and materials in this study are available from the corresponding author upon reasonable request.

## References

[1] GBD 2017 Disease and Injury Incidence and Prevalence Collaborators (2018). Global, regional, and national incidence, prevalence, and years lived with disability for 354 diseases and injuries for 195 countries and territories, 1990-2017: a systematic analysis for the Global Burden of Disease Study 2017. Lancet.

[2] Holtzheimer PE, Mayberg HS (2011). Stuck in a rut: rethinking depression and its treatment. Trends Neurosci..

[3] Boldrini M (2013). Hippocampal granule neuron number and dentate gyrus volume in antidepressant-treated and untreated major depression. Neuropsychopharmacology.

[4] Lucassen PJ, Stumpel MW, Wang Q, Aronica E (2010). Decreased numbers of progenitor cells but no response to antidepressant drugs in the hippocampus of elderly depressed patients. Neuropharmacology.

[5] Boldrini M (2019). Resilience is associated with larger dentate gyrus, while suicide decedents with major depressive disorder have fewer granule neurons. Biol. Psychiatry.

[6] Santarelli L (2003). Requirement of hippocampal neurogenesis for the behavioral effects of antidepressants. Science.

[7] Mateus-Pinheiro A (2013). Sustained remission from depressive-like behavior depends on hippocampal neurogenesis. Transl. Psychiatry.

[8] Denoth-Lippuner A, Jessberger S (2021). Formation and integration of new neurons in the adult hippocampus. Nat. Rev. Neurosci..

[9] Goncalves JT, Schafer ST, Gage FH (2016). Adult neurogenesis in the hippocampus: from stem cells to behavior. Cell.

[10] Bond AM, Ming GL, Song H (2015). Adult mammalian neural stem cells and neurogenesis: five decades later. Cell Stem Cell.

[11] Malberg JE, Duman RS (2003). Cell proliferation in adult hippocampus is decreased by inescapable stress: reversal by fluoxetine treatment. Neuropsychopharmacology.

[12] Bento CF (2016). Mammalian autophagy: how does it work?. Annu. Rev. Biochem..

[13] Yamamoto A, Yue Z (2014). Autophagy and its normal and pathogenic states in the brain. Annu. Rev. Neurosci..

[14] Ohashi Y (2021). Class III phosphatidylinositol 3-kinase complex I subunit NRBF2/Atg38 - from cell and structural biology to health and disease. Autophagy.

[15] Zhong Y (2009). Distinct regulation of autophagic activity by Atg14L and Rubicon associated with Beclin 1-phosphatidylinositol-3-kinase complex. Nat. Cell Biol..

[16] Matsunaga K (2009). Two Beclin 1-binding proteins, Atg14L and Rubicon, reciprocally regulate autophagy at different stages. Nat. Cell Biol..

[17] Sun Q (2008). Identification of Barkor as a mammalian autophagy-specific factor for Beclin 1 and class III phosphatidylinositol 3-kinase. Proc. Natl. Acad. Sci. USA.

[18] Young LN, Cho K, Lawrence R, Zoncu R, Hurley JH (2016). Dynamics and architecture of the NRBF2-containing phosphatidylinositol 3-kinase complex I of autophagy. Proc. Natl. Acad. Sci. USA.

[19] Lu J (2014). NRBF2 regulates autophagy and prevents liver injury by modulating Atg14L-linked phosphatidylinositol-3 kinase III activity. Nat. Commun..

[20] Araki Y (2013). Atg38 is required for autophagy-specific phosphatidylinositol 3-kinase complex integrity. J. Cell Biol..

[21] Cai CZ (2021). NRBF2 is a RAB7 effector required for autophagosome maturation and mediates the association of APP-CTFs with active form of RAB7 for degradation. Autophagy.

[22] Kim YJ (2021). An autophagy-related protein Becn2 regulates cocaine reward behaviors in the dopaminergic system. Sci. Adv..

[23] Xiao X, Shang X, Zhai B, Zhang H, Zhang T (2018). Nicotine alleviates chronic stress-induced anxiety and depressive-like behavior and hippocampal neuropathology via regulating autophagy signaling. Neurochem. Int..

[24] Gulbins A (2018). Antidepressants act by inducing autophagy controlled by sphingomyelin-ceramide. Mol. Psychiatry.

[25] Wang C, Liang CC, Bian ZC, Zhu Y, Guan JL (2013). FIP200 is required for maintenance and differentiation of postnatal neural stem cells. Nat. Neurosci..

[26] Xi Y (2016). Knockout of Atg5 delays the maturation and reduces the survival of adult-generated neurons in the hippocampus. Cell Death Dis.

[27] Wang M (2021). WDR81 regulates adult hippocampal neurogenesis through endosomal SARA-TGFbeta signaling. Mol. Psychiatry.

[28] Berg DA (2019). A common embryonic origin of stem cells drives developmental and adult neurogenesis. Cell.

[29] Shin J (2015). Single-cell RNA-seq with waterfall reveals molecular cascades underlying adult neurogenesis. Cell Stem Cell.

[30] Hao ZZ (2022). Single-cell transcriptomics of adult macaque hippocampus reveals neural precursor cell populations. Nat. Neurosci..

[31] Snyder JS, Soumier A, Brewer M, Pickel J, Cameron HA (2011). Adult hippocampal neurogenesis buffers stress responses and depressive behaviour. Nature.

[32] Surget A, Belzung C (2022). Adult hippocampal neurogenesis shapes adaptation and improves stress response: a mechanistic and integrative perspective. Mol. Psychiatry.

[33] Klionsky DJ (2021). Guidelines for the use and interpretation of assays for monitoring autophagy (4th edition). Autophagy.

[34] Zhou Y (2018). Autocrine Mfge8 Signaling Prevents Developmental Exhaustion of the Adult Neural Stem Cell Pool. Cell Stem Cell.

[35] Tang C (2019). Neural stem cells behave as a functional niche for the maturation of newborn neurons through the secretion of PTN. Neuron.

[36] Akers KG (2014). Hippocampal neurogenesis regulates forgetting during adulthood and infancy. Science.

[37] Schaffner I (2018). FoxO function is essential for maintenance of autophagic flux and neuronal morphogenesis in adult neurogenesis. Neuron.

[38] Li K (2019). SAR405, a highly specific VPS34 inhibitor, disrupts auditory fear memory consolidation of mice via facilitation of inhibitory neurotransmission in basolateral amygdala. Biol. Psychiatry.

[39] Ronan B (2014). A highly potent and selective Vps34 inhibitor alters vesicle trafficking and autophagy. Nat. Chem. Biol..

[40] Cheng J, Umschweif G, Leung J, Sagi Y, Greengard P (2019). HCN2 channels in cholinergic interneurons of nucleus accumbens shell regulate depressive behaviors. Neuron.

[41] Lagace DC (2007). Dynamic contribution of nestin-expressing stem cells to adult neurogenesis. J. Neurosci..

[42] Tunc-Ozcan E (2019). Activating newborn neurons suppresses depression and anxiety-like behaviors. Nat. Commun..

[43] Moreno-Jimenez EP (2019). Adult hippocampal neurogenesis is abundant in neurologically healthy subjects and drops sharply in patients with Alzheimer’s disease. Nat. Med..

[44] Sorrells SF (2018). Human hippocampal neurogenesis drops sharply in children to undetectable levels in adults. Nature.

[45] Wang W (2022). Transcriptome dynamics of hippocampal neurogenesis in macaques across the lifespan and aged humans. Cell Res..

[46] Terreros-Roncal J (2021). Impact of neurodegenerative diseases on human adult hippocampal neurogenesis. Science.

[47] Kempermann G (2018). Human adult neurogenesis: evidence and remaining questions. Cell Stem Cell.

[48] Fang J, Demic S, Cheng S (2018). The reduction of adult neurogenesis in depression impairs the retrieval of new as well as remote episodic memory. PLoS ONE.

[49] Boldrini M (2012). Hippocampal angiogenesis and progenitor cell proliferation are increased with antidepressant use in major depression. Biol. Psychiatry.

[50] Anacker C (2014). Adult hippocampal neurogenesis in depression: behavioral implications and regulation by the stress system. Curr. Top. Behav. Neurosci..

[51] Marino G, Madeo F, Kroemer G (2011). Autophagy for tissue homeostasis and neuroprotection. Curr. Opin. Cell Biol..

[52] Kara NZ, Flaisher-Grinberg S, Anderson GW, Agam G, Einat H (2018). Mood-stabilizing effects of rapamycin and its analog temsirolimus: relevance to autophagy. Behav. Pharmacol..

[53] Bjorkoy G (2005). p62/SQSTM1 forms protein aggregates degraded by autophagy and has a protective effect on huntingtin-induced cell death. J. Cell Biol..

[54] Kageyama S (2021). p62/SQSTM1-droplet serves as a platform for autophagosome formation and anti-oxidative stress response. Nat. Commun..

[55] Babu JR (2008). Genetic inactivation of p62 leads to accumulation of hyperphosphorylated tau and neurodegeneration. J. Neurochem..

[56] Mitroi DN (2017). SGPL1 (sphingosine phosphate lyase 1) modulates neuronal autophagy via phosphatidylethanolamine production. Autophagy.

[57] Cao YY (2014). NRBF2 regulates macroautophagy as a component of Vps34 Complex I. Biochem. J..

[58] Ma X (2017). MTORC1-mediated NRBF2 phosphorylation functions as a switch for the class III PtdIns3K and autophagy. Autophagy.

[59] Zeng HH (2021). Autophagy protein NRBF2 attenuates endoplasmic reticulum stress-associated neuroinflammation and oxidative stress via promoting autophagosome maturation by interacting with Rab7 after SAH. J. Neuroinflamm..

[60] Zhong Y (2014). Nrbf2 protein suppresses autophagy by modulating Atg14L protein-containing Beclin 1-Vps34 complex architecture and reducing intracellular phosphatidylinositol-3 phosphate levels. J. Biol. Chem..

[61] Nakatogawa H (2020). Mechanisms governing autophagosome biogenesis. Nat. Rev. Mol. Cell Biol..

[62] Melia TJ, Lystad AH, Simonsen A (2020). Autophagosome biogenesis: from membrane growth to closure. J. Cell Biol..

[63] Wang C (2016). Elevated p62/SQSTM1 determines the fate of autophagy-deficient neural stem cells by increasing superoxide. J. Cell Biol..

[64] Vazquez P (2012). Atg5 and Ambra1 differentially modulate neurogenesis in neural stem cells. Autophagy.

[65] Yazdankhah M, Farioli-Vecchioli S, Tonchev AB, Stoykova A, Cecconi F (2014). The autophagy regulators Ambra1 and Beclin 1 are required for adult neurogenesis in the brain subventricular zone. Cell Death Dis.

[66] Morrow CS (2020). Vimentin coordinates protein turnover at the aggresome during neural stem cell quiescence exit. Cell Stem Cell.

[67] Leeman DS (2018). Lysosome activation clears aggregates and enhances quiescent neural stem cell activation during aging. Science.

[68] Audesse AJ (2019). FOXO3 directly regulates an autophagy network to functionally regulate proteostasis in adult neural stem cells. PLoS Genet..

[69] Khawaja X, Xu J, Liang JJ, Barrett JE (2004). Proteomic analysis of protein changes developing in rat hippocampus after chronic antidepressant treatment: implications for depressive disorders and future therapies. J. Neurosci. Res..

[70] McHugh PC, Rogers GR, Glubb DM, Joyce PR, Kennedy MA (2010). Proteomic analysis of rat hippocampus exposed to the antidepressant paroxetine. J. Psychopharmacol..

[71] Cheng A, Wang S, Cai J, Rao MS, Mattson MP (2003). Nitric oxide acts in a positive feedback loop with BDNF to regulate neural progenitor cell proliferation and differentiation in the mammalian brain. Dev. Biol..

[72] Matsumata M (2012). The effects of Fabp7 and Fabp5 on postnatal hippocampal neurogenesis in the mouse. Stem Cells.

[73] Schnell A (2014). The nuclear receptor REV-ERBalpha regulates Fabp7 and modulates adult hippocampal neurogenesis. PLoS ONE.

[74] Zhong L (2017). Quantitative proteomics reveals EVA1A-related proteins involved in neuronal differentiation. Proteomics.

[75] Francisco A (2020). Mitochondrial NAD(P)(+) transhydrogenase is unevenly distributed in different brain regions, and its loss causes depressive-like dehavior and motor dysfunction in mice. Neuroscience.

[76] Stankiewicz AM (2014). Social stress increases expression of hemoglobin genes in mouse prefrontal cortex. BMC Neurosci..

[77] Owada Y (2006). Altered emotional behavioral responses in mice lacking brain-type fatty acid-binding protein gene. Eur. J. Neurosci..

[78] Ren Y (2021). Milk fat globule-EGF factor 8 alleviates pancreatic fibrosis by inhibiting ER stress-induced chaperone-mediated autophagy in mice. Front. Pharmacol..

[79] Yasumo H (2000). Nuclear receptor binding factor-2 (NRBF-2), a possible gene activator protein interacting with nuclear hormone receptors. Biochim. Biophys. Acta.

[80] Sidibe DK, Vogel MC, Maday S (2022). Organization of the autophagy pathway in neurons. Curr. Opin. Neurobiol..

[81] Overhoff M (2022). Autophagy regulates neuronal excitability by controlling cAMP/protein kinase A signaling at the synapse. EMBO J..

[82] Mira H (2010). Signaling through BMPR-IA regulates quiescence and long-term activity of neural stem cells in the adult hippocampus. Cell Stem Cell.

[83] Percie du Sert N (2020). The ARRIVE guidelines 2.0: updated guidelines for reporting animal research. Br. J. Pharmacol..

[84] He JG (2021). Transcription factor TWIST1 integrates dendritic remodeling and chronic stress to promote depressive-like behaviors. Biol. Psychiatry.

[85] Luo H (2020). Angiotensin-converting enzyme inhibitor rapidly ameliorates depressive-type behaviors via bradykinin-dependent activation of mammalian target of rapamycin complex 1. Biol. Psychiatry.

[86] David DJ (2009). Neurogenesis-dependent and -independent effects of fluoxetine in an animal model of anxiety/depression. Neuron.

[87] Li MX (2018). Gene deficiency and pharmacological inhibition of caspase-1 confers resilience to chronic social defeat stress via regulating the stability of surface AMPARs. Mol. Psychiatry.

[88] Bacq A (2012). Organic cation transporter 2 controls brain norepinephrine and serotonin clearance and antidepressant response. Mol. Psychiatry.

[89] Taha E (2020). eEF2/eEF2K pathway in the mature dentate gyrus determines neurogenesis level and cognition. Curr. Biol..

[90] Cui QQ (2020). Hippocampal CD39/ENTPD1 promotes mouse depression-like behavior through hydrolyzing extracellular ATP. EMBO Rep..

[91] Zhang SQ (2022). Repeated vagus nerve stimulation produces anxiolytic effects via upregulation of AMPAR function in centrolateral amygdala of male rats. Neurobiol. Stress.

[92] Wei C (2022). LncRNA HOXA11-AS promotes glioma malignant phenotypes and reduces its sensitivity to ROS via Tpl2-MEK1/2-ERK1/2 pathway. Cell Death Dis..

