# Supplementary material for: Cell type-specific NRBF2 orchestrates autophagic flux and adult hippocampal neurogenesis in chronic stress-induced depression
Source: Cell Discov. 2023 Aug 29;9:90. doi: 10.1038/s41421-023-00583-7 (PMC10465581; doi:10.1038/s41421-023-00583-7)
Supplement: Supplementary file 1 — Supplementary Information [file 41421_2023_583_MOESM1_ESM.pdf]

Cell type-specific NRBF2 orchestrates autophagic flux and adult hippocampal neurogenesis in chronic stress-induced depression

Shao-Qi Zhang[1,7], Qiao Deng[1,7], Qi Zhu[2,7], Zhuang-Li Hu[1,3,4,5], Li-Hong Long[1,3,4,5], Peng-Fei Wu[1,4,5], Jin-Gang He[1,4,5], Hong-Sheng Chen[1,3], Zhenyu Yue[6], Jia-Hong Lu[2,✉], Fang Wang[1,3,4,5,✉], & Jian-Guo Chen[1,3,4,5,✉]

[1]Department of Pharmacology, School of Basic Medicine, Tongji Medical College, Huazhong University of Science and Technology, Wuhan, Hubei, China

[2]State Key Laboratory of Quality Research in Chinese Medicine, Institute of Chinese Medical Sciences, University of Macau, Macau SAR, China

[3]The Key Laboratory of Neurological Diseases (HUST), Ministry of Education of China, Wuhan, Hubei, China

[4]Laboratory of Neuropsychiatric Diseases, The Institute of Brain Research, Huazhong University of Science and Technology, Wuhan, Hubei, China

[5]The Key Laboratory for Drug Target Researches and Pharmacodynamic Evaluation of Hubei Province, Wuhan, Hubei, China

[6]Department of Neuroscience, Friedman Brain Institute, Icahn School of Medicine at Mount Sinai, New York, NY, USA

[7]These authors contributed equally: Shao-Qi Zhang, Qiao Deng, Qi Zhu.

✉e-mail: chenj@mails.tjmu.edu.cn; wangfanghust@hust.edu.cn; jiahonglu@um.edu.mo

Running title: aNSCs-specific NRBF2 regulates depression-like behavior

Supplementary figures

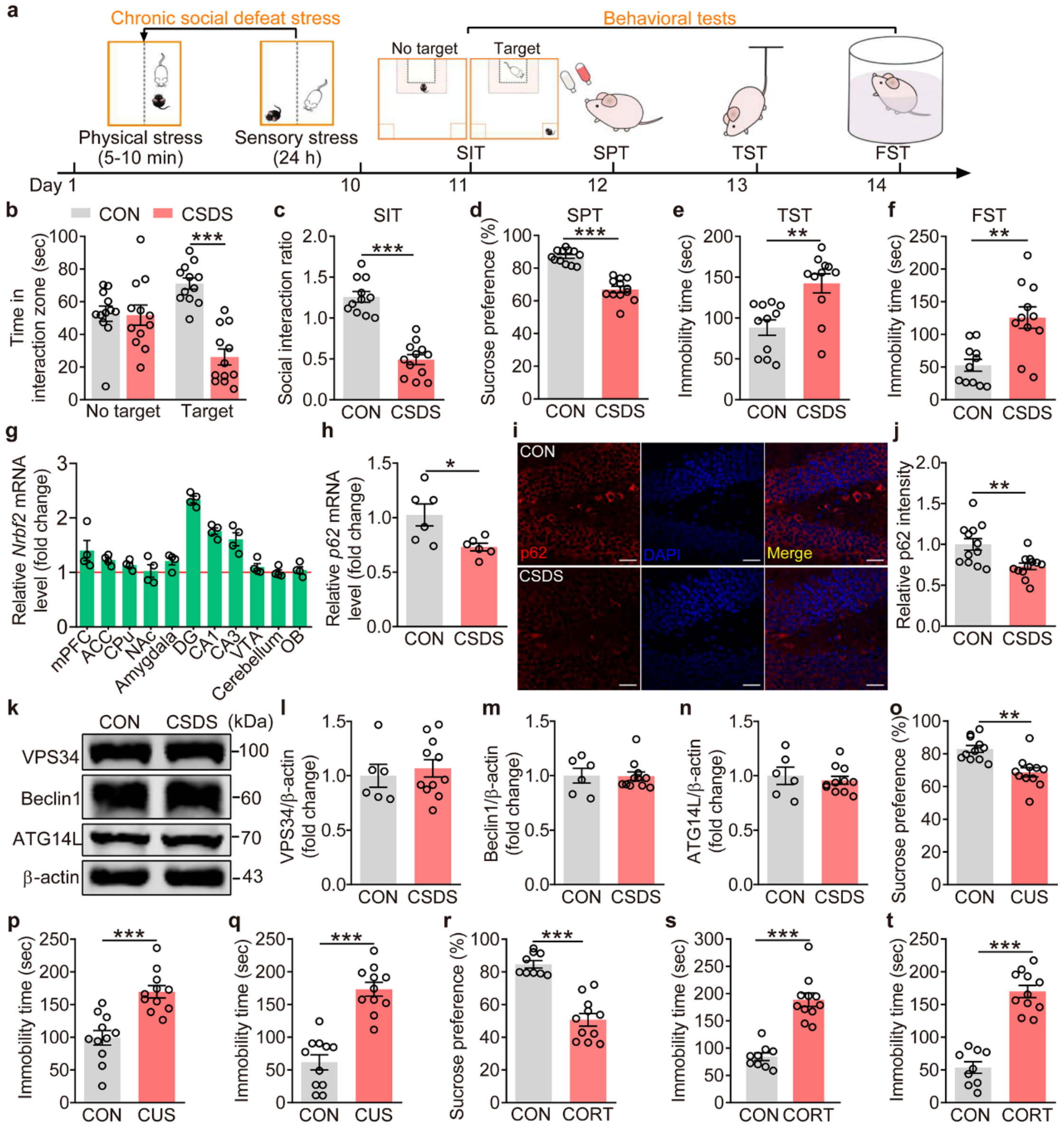

Supplementary Fig. S1. Chronic stress induces depression-like behavior in mice.

a Experimental paradigms for CSDS and behavioral tests.

b-f Depression-like behavior in CSDS-exposed mice, as measured by the social interaction time (**b**) and social interaction ratio (**c**) in SIT, the sucrose preference in SPT (**d**), and the immobility time in TST (**e**) and FST (**f**) (*n* = 11-12 mice per group).

g *Nrbf2* mRNA level in different brain areas of adult naive mice (*n* = 4 mice per group). mPFC, medial prefrontal cortex; ACC, anterior cingulate cortex; CPu, caudate putamen; NAc, nucleus accumbens; DG, dentate gyrus, CA1, cornu ammonis 1; CA3, cornu ammonis 3; VTA, ventral tegmental area; OB, olfactory bulb.

h Quantitative real-time PCR result showing the *p62* mRNA level in the DG (*n* = 6 mice per group).

i, j Representative images (**i**) and quantification (**j**) of p62 intensity in the DG (*n* = 12 slices per

group). Scale bars, 30 μm.

k-n Representative images (**k**) and quantification of the protein expressions of VPS34 (**l**), Beclin1 (**m**), and ATG14L (**n**) in the DG (*n* = 6-11 mice per group).

o-q Behavioral tests of SPT (**o**), TST (**p**), and FST (**q**) in control and CUS-treated mice (*n* = 11 mice per group).

r-t Behavioral tests of SPT (**r**), TST (**s**) and FST (**t**) in control and CORT-treated mice (*n* = 9-11 mice per group).

Data are presented as mean ± SEM and analyzed by two-sided unpaired t test (**c-f**, **h**, **j**, **l-t**) or two-way ANOVA following by Bonferroni's post hoc test (**b**). $^{*}P < 0.05$, $^{**}P < 0.01$, and $^{***}P < 0.001$.

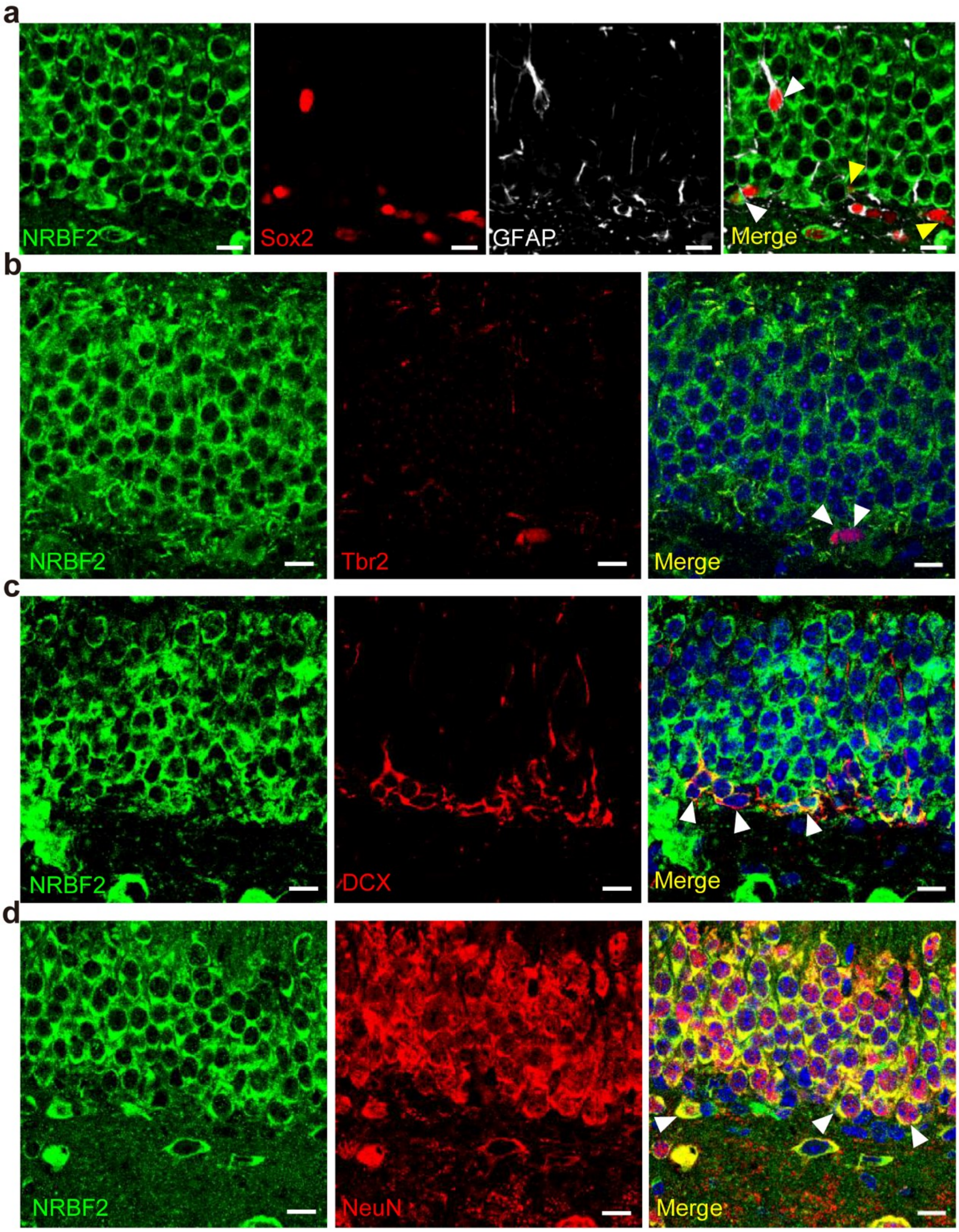

Supplementary Fig. S2 The NRBF2 expression in dentate gyrus of adult mice.

a Immunofluorescent analysis of NRBF2 expression in Sox2^{+}GFAP^{+} type 1 NSCs and Sox2^{+}GFAP^{-} type 2 NSCs. White arrows indicating NRBF2^{+}Sox2^{+}GFAP^{+} type 1 NSCs and yellow arrows indicating NRBF2^{+}Sox2^{+}GFAP^{-} type 2 NSCs. Scale bars, 10 μm.

b Immunofluorescent analysis of NRBF2 expression in Tbr2^{+} IPCs. White arrow indicating NRBF2^{+}Tbr2^{+} IPCs. Scale bars, 10 μm.

c, d Immunofluorescent analysis of NRBF2 expression in DCX^{+} neuroblasts (**c**) and NeuN^{+} neurons (**d**) in the DG. White arrows indicating NRBF2^{+}DCX^{+} neuroblasts or NRBF2^{+}NeuN^{+} neurons. Scale

bars, 10 μm.

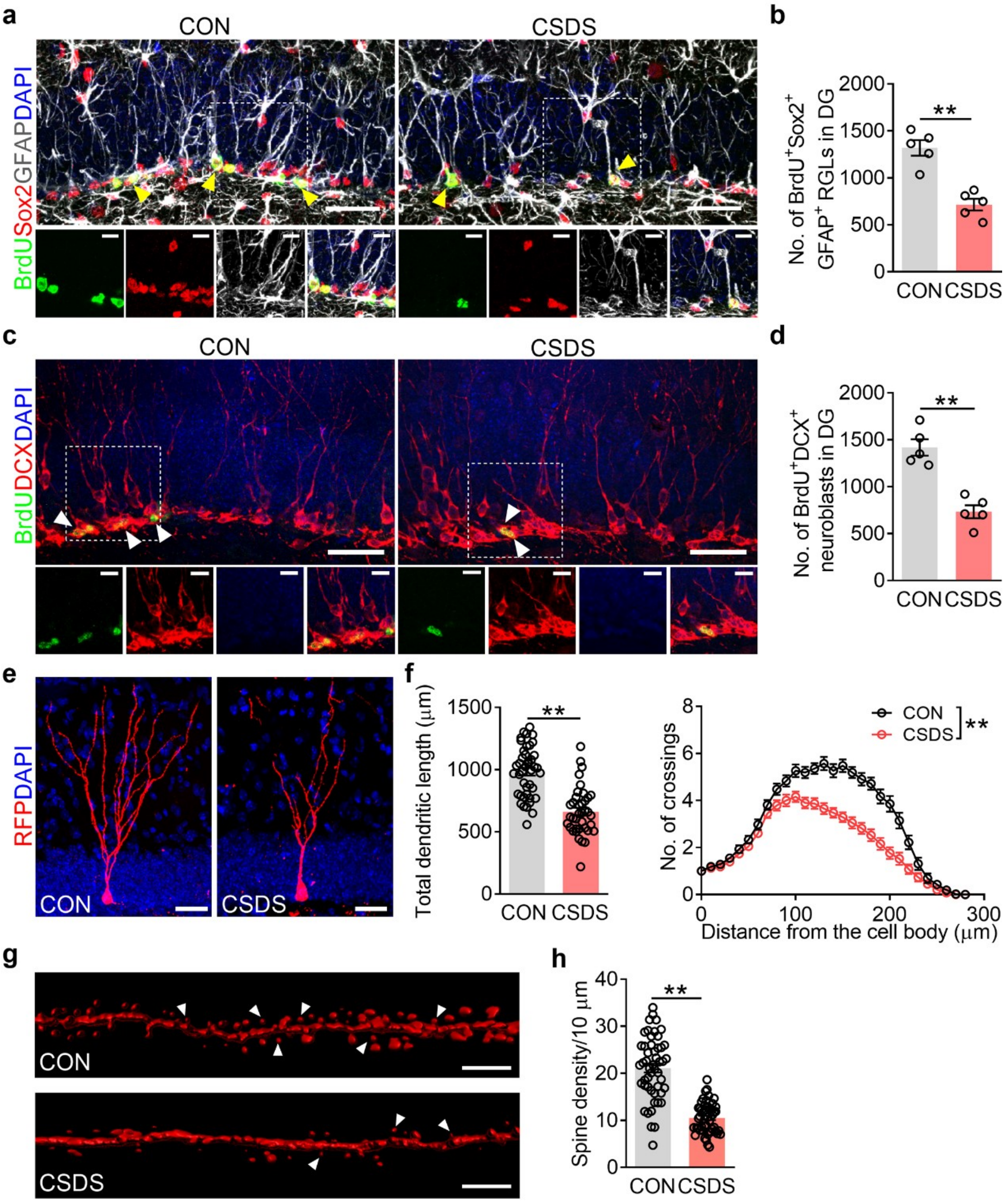

Supplementary Fig. S3 CSDS impairs AHN and the development of newborn neuron in the DG of mice.

a Representative confocal image of BrdU$^+$Sox2$^+$GFAP$^+$ RGLs in the DG of control and CSDS-exposed mice. Bottom panels: higher magnification views of selected regions (white square), BrdU (green), Sox2 (red), GFAP (white), and DAPI (blue). Yellow arrows indicating BrdU$^+$Sox2$^+$GFAP$^+$ RGLs. Scale bars, 30 μm (overview) and 10 μm (zoom).

b Quantifications showing the number of BrdU$^+$Sox2$^+$GFAP$^+$ RGLs in the DG (n = 5 mice per group).

c, d Representative images (**c**) and quantifications (**d**) showing the number of BrdU$^+$DCX$^+$ neuroblasts in the DG (n = 5 mice per group). BrdU (green), DCX (red), and DAPI (blue). White arrows indicating BrdU$^+$DCX$^+$ neuroblasts. Scale bars, 30 μm (overview) and 10 μm (zoom).

e, f Representative confocal images (**e**) and quantifications of the dendritic length (**f**, left) and dendritic complexity (**f**, right) in RFP^+ newborn neurons (*n* = 38-42 cells per group). Scale bars, 30 μm.

g, h Representative images (**g**) and quantifications (**h**) of dendritic spines density of RFP^+ newborn neurons (*n* = 50 segments per group). White arrows indicating spines. Scale bars, 5 μm.

Data are presented as mean ± SEM and analyzed by two-sided unpaired t test (**b**, **d**, **f**, **h**) or repeated measures ANOVA (**f**, right). $^{**}P < 0.01$ and $^{***}P < 0.001$.

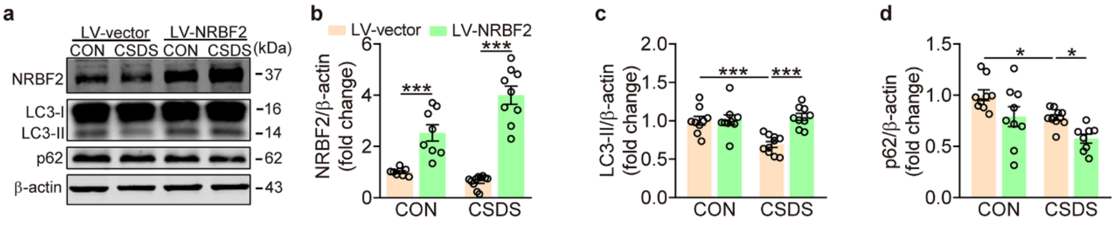

Supplementary Fig. S4 Overexpression of NRBF2 ameliorates CSDS-induced autophagic deficiency in the DG.

a-d Representative images (**a**) and quantifications of NRBF2 (**b**), LC3-II (**c**) and p62 (**d**) protein expression in the DG upon control and CSDS-exposed mice treated with LV-vector or LV-NRBF2 (n = 8-11 mice per group).

Data are presented as mean ± SEM and analyzed by two-way ANOVA following by Bonferroni's post hoc test (**b-d**). *$P < 0.05$ and ***$P < 0.001$.

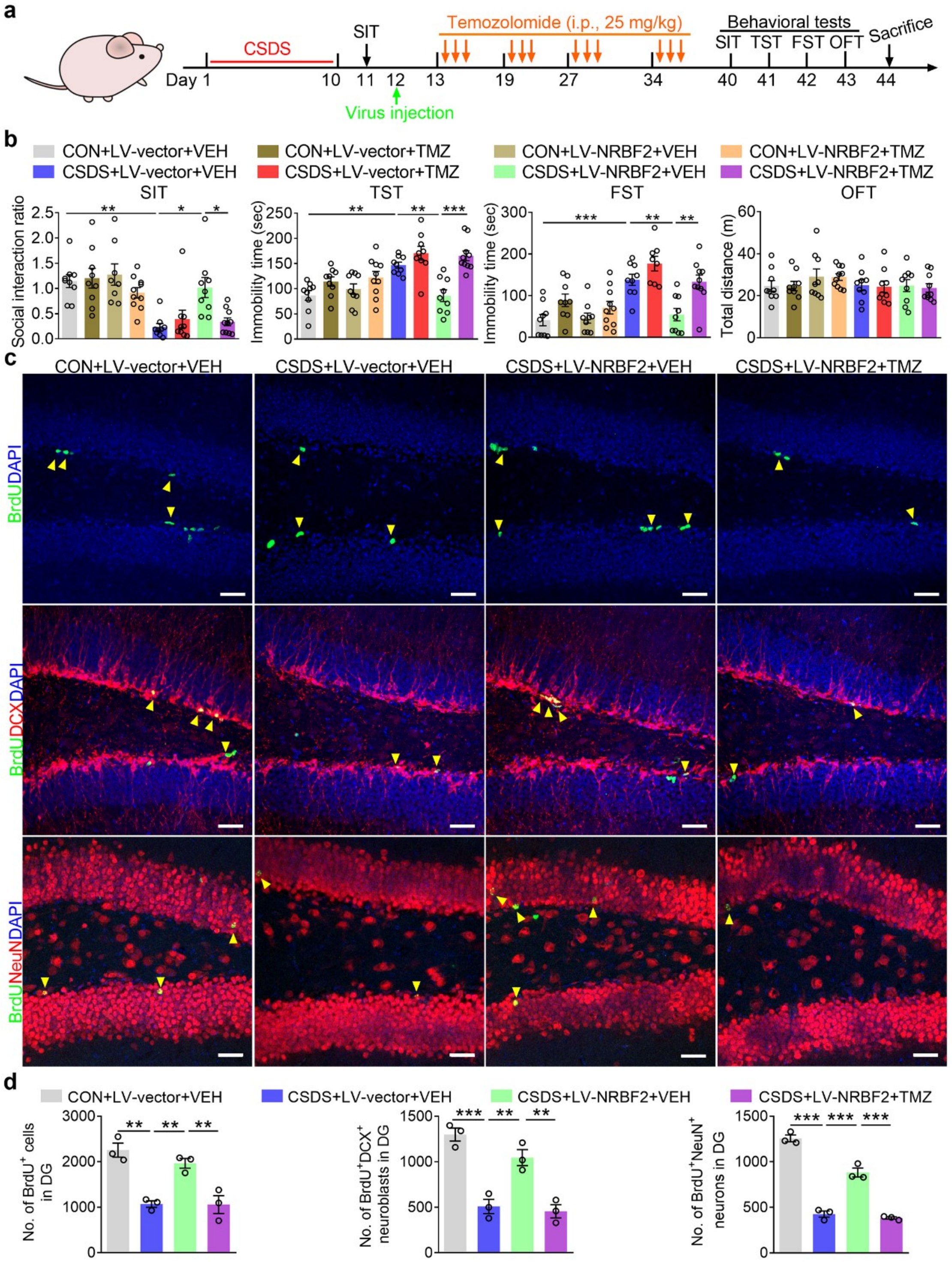

Supplementary Fig. S5 Chronic temozolomide treatment abolishes the antidepressant and proneurogenic effects of NRBF2 overexpression.

a Timeline of experiments.

b Behavioral tests of SIT, TST, FST, and OFT in control and CSDS-exposed mice expressing LV-vector or LV-NRBF2, and following by treatment with temozolomide (TMZ) for 4 weeks (n = 8-10 mice per group).

c Representative images of BrdU^{+} cells, BrdU^{+}DCX^{+} neuroblasts, and BrdU^{+}NeuN^{+} neurons in the

DG of CON + LV-vector + VEH, CSDS + LV-vector + VEH, CSDS + LV-NRBF2 + VEH, and CSDS + LV-NRBF2 + TMZ group. Yellow arrows indicating $BrdU^+$ and $marker^+$ cells. Scale bars, 30 μm.

d Quantification of $BrdU^+$ cells, $BrdU^+DCX^+$ neuroblasts, and $BrdU^+NeuN^+$ neurons in the DG (*n* = 3 mice per group).

Data are presented as mean ± SEM and analyzed by one-way ANOVA following by Tukey's post hoc test (**b**, **d**). *$P < 0.05$, **$P < 0.01$, and ***$P < 0.001$.

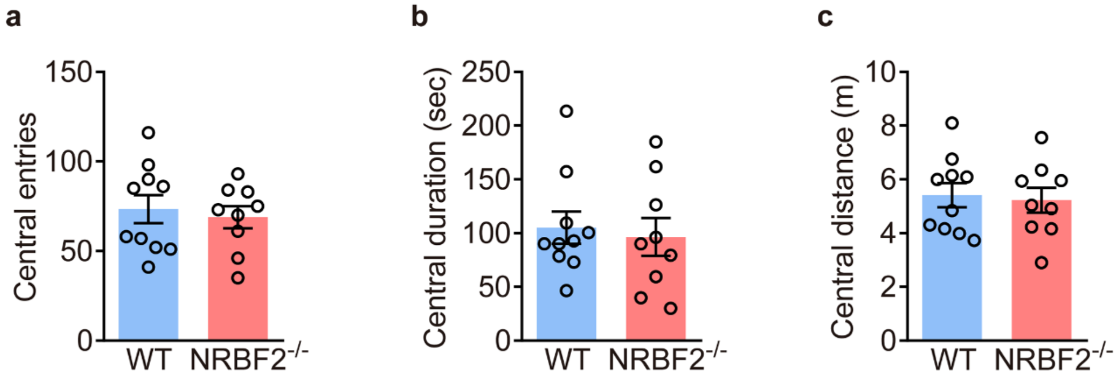

Supplementary Fig. S6 NRBF2 deletion does not lead to anxiety-like behavior of mice.
a-c Behavioral tests of OFT, such as central entries, central duration and central distance, in WT and NRBF2$^{-/-}$ mice (n = 9-10 mice per group).
Data are presented as mean ± SEM and analyzed by two-sided unpaired t test (**a-c**).

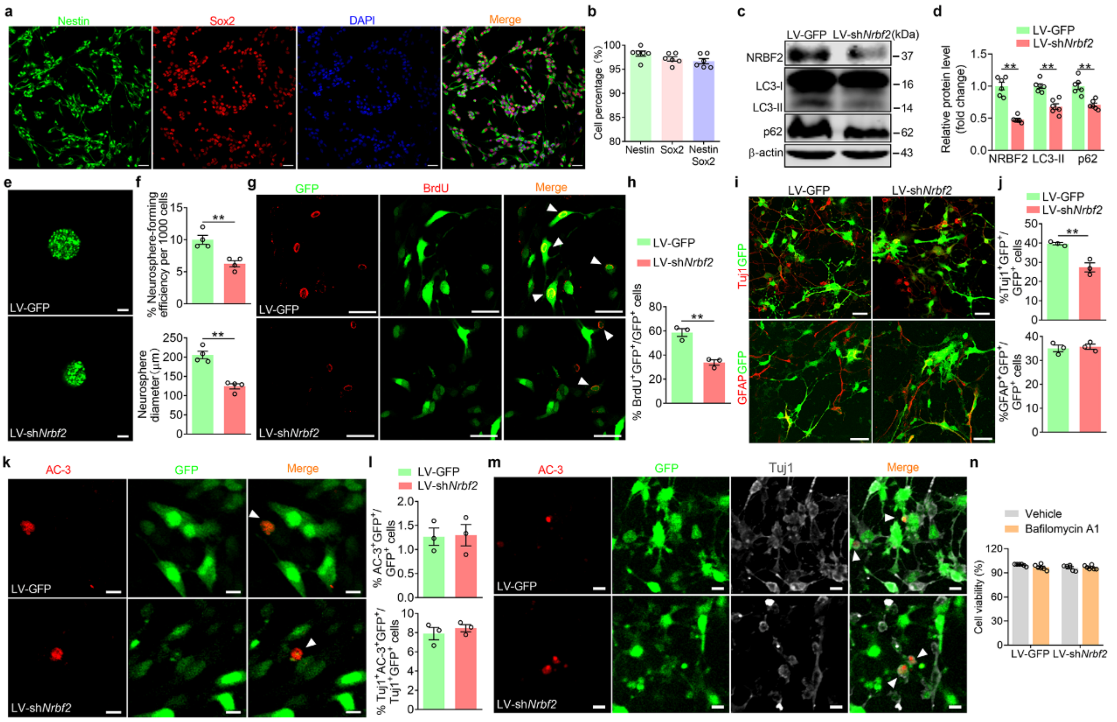

Supplementary Fig. S7 Deficiency of NRBF2 impairs proliferation and neural differentiation, but not apoptosis in adult neural stem cells.

a, b Representative images (**a**) and quantifications (**b**) of Nestin$^+$Sox2$^+$ aNSCs, Nestin (green), Sox2 (red), DAPI (blue). Scale bars, 50 μm. The majority of aNSCs (96.69%) expressed both neural stem cell markers Nestin and Sox2 (n = 6 independent experiments per group).

c, d Representative images (**c**) and quantifications (**d**) of NRBF2, LC3-II and p62 protein expression in aNSCs treated with LV-sh*Nrbf2* or LV-GFP (n = 6 independent experiments per group).

e Representative images of neurospheres. Scale bars, 50 μm.

f Quantification of the neurosphere-forming efficiency and the diameter of neurospheres (n = 4 independent experiments per group).

g, h Representative images (**g**) and quantifications (**h**) of proliferating aNSCs, followed by BrdU pulse labeling and immunofluorescent analysis (n = 3 independent experiments per group). White arrows indicating BrdU$^+$GFP$^+$ cells. Scale bars, 30 μm.

i Representative images of differentiated aNSCs, followed by immunostaining with the neuronal marker, Tuj1, and the astrocytic marker, GFAP. Scale bars, 30 μm.

j Quantification of the percentage of differentiated Tuj1$^+$GFP$^+$ neurons and GFAP$^+$GFP$^+$ astrocytes (n = 3 independent experiments per group).

k Representative images of proliferating aNSCs, followed by immunostaining with activated caspase-3 (AC-3). White arrows indicating AC-3$^+$GFP$^+$ cells. Scale bars, 10 μm.

l Quantification of the percentage of AC-3$^+$GFP$^+$ proliferating aNSCs or Tuj1$^+$AC-3$^+$GFP$^+$ differentiated aNSCs (n = 3 independent experiments per group).

m Representative images of differentiated aNSCs, followed by immunostaining with Tuj1 and AC-3. White arrows indicating Tuj1$^+$AC-3$^+$GFP$^+$ cells. Scale bars, 10 μm.

n Quantification of the cell viability in control and NRBF2-deficient aNSCs treated with vehicle (0.1% DMSO) or bafilomycin A1 (n = 6 independent experiments per group).

Data are presented as mean ± SEM and analyzed by two-sided unpaired t test (**d**, **f**, **h**, **j, l**) or two-way ANOVA following by Bonferroni's post hoc test (**n**). **$P < 0.01$.

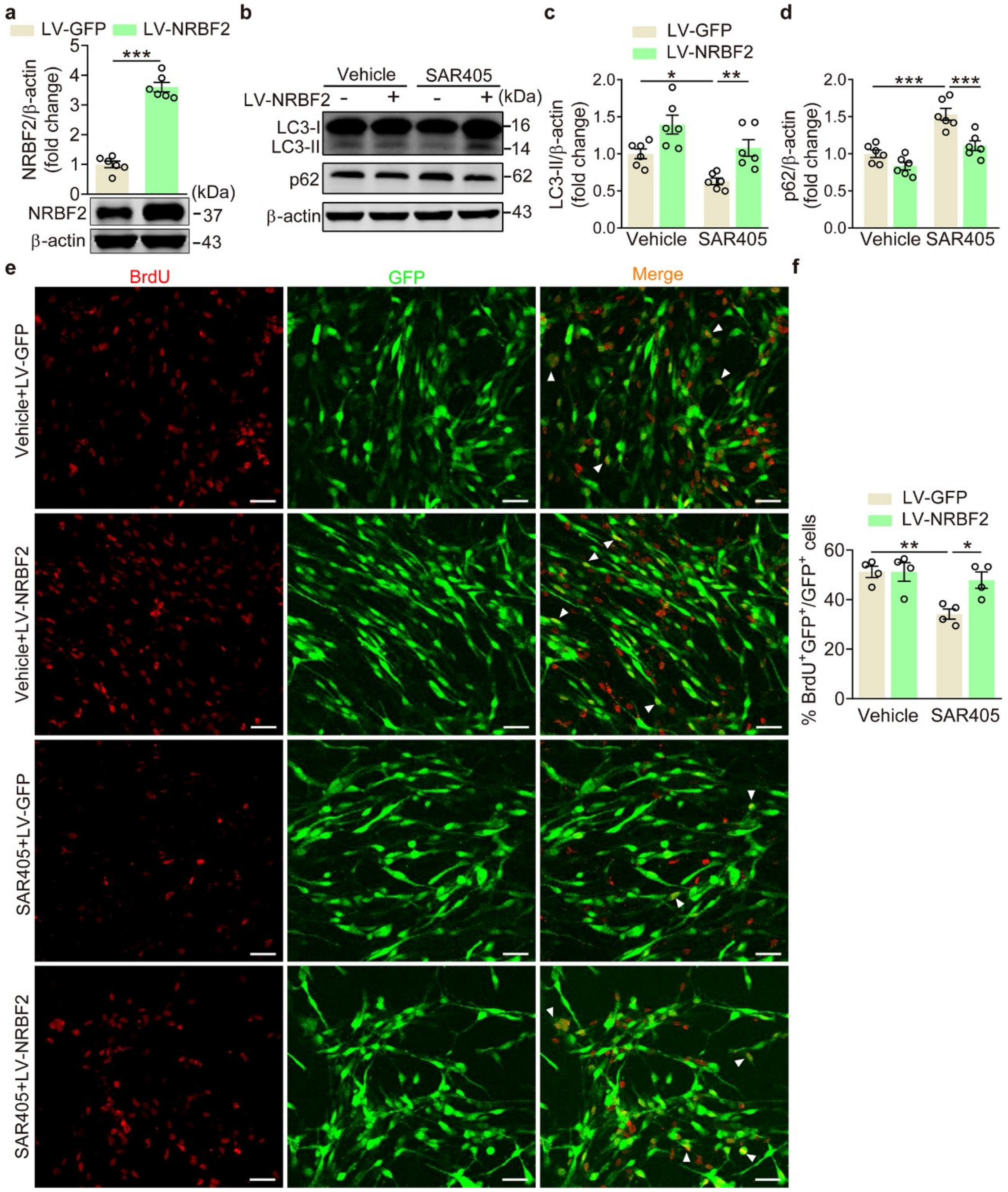

Supplementary Fig. S8 NRBF2 overexpression ameliorates SAR405-induced inhibition of autophagy and proliferated deficiency of aNSCs *in vitro*.

a Representative western blotting images and quantification of NRBF2 protein expression in aNSCs transfected with LV-GFP or LV-NRBF2 ($n = 6$ independent experiments per group).

b-d Representative western blotting images (**b**) and quantification of LC3-II (**c**) and p62 (**d**) protein expression in vehicle (0.1% DMSO) and SAR405-treated aNSCs transfected with LV-GFP or LV-NRBF2 ($n = 6$ independent experiments per group).

e, **f** Representative images (**e**) and quantifications (**f**) of proliferating aNSCs transfected with LV-GFP or LV-NRBF2, followed by SAR405 treatment and BrdU pulse labeling ($n = 4$ independent experiments per group). White arrows indicating $BrdU^+GFP^+$ cells. Scale bars, 30 μm.

Data are presented as mean ± SEM and analyzed by two-sided unpaired t test (**a**) or two-way

ANOVA following by Bonferroni's post hoc test (**c**, **d, f**). $*P < 0.05$, $**P < 0.01$, and $***P < 0.001$.

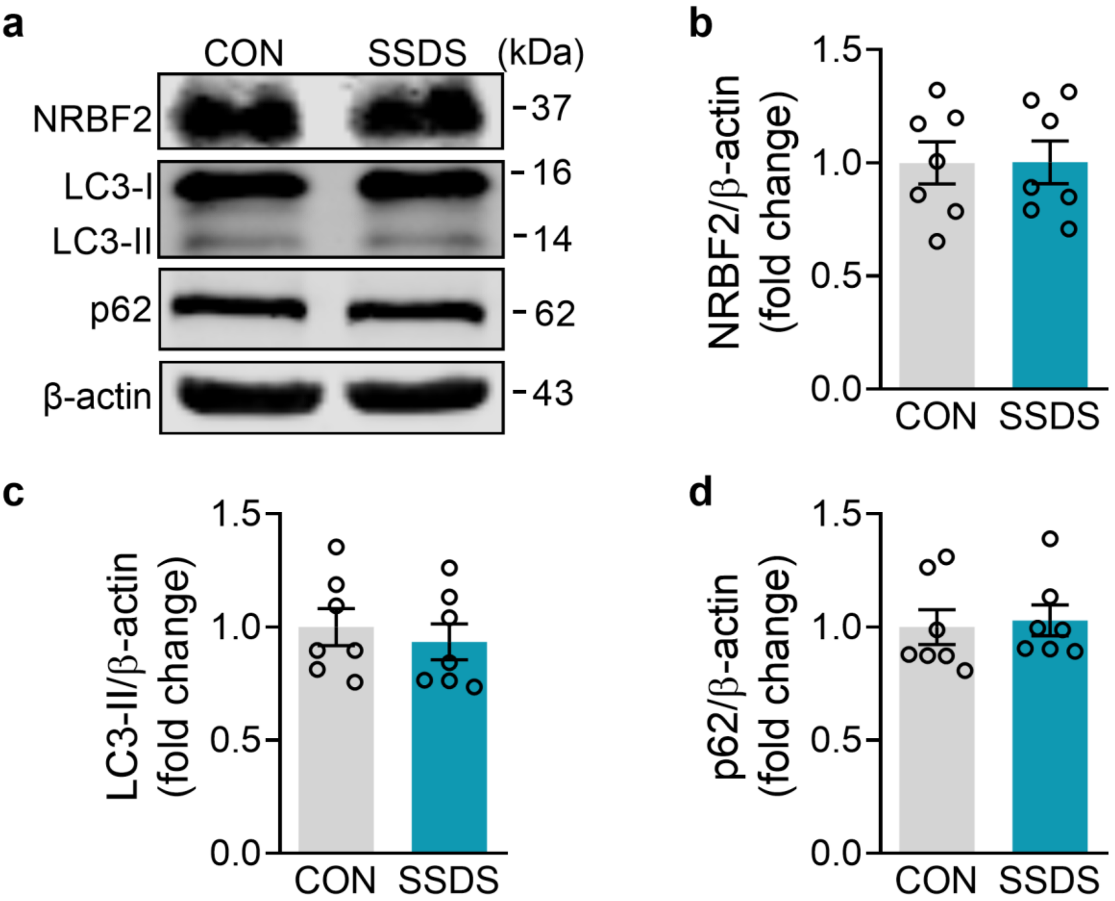

Supplementary Fig. S9 Subthreshold social defeat stress does not affect the expression of NRBF2 in the DG.

a-d Representative western blotting images (**a**) and quantification of NRBF2 (**b**), LC3-II (**c**) and p62 (**d**) protein expression in control and subthreshold social defeat stress (SSDS)-treated mice (*n* = 7 mice per group).

Data are presented as mean ± SEM and analyzed by two-sided unpaired t test (**b-d**).

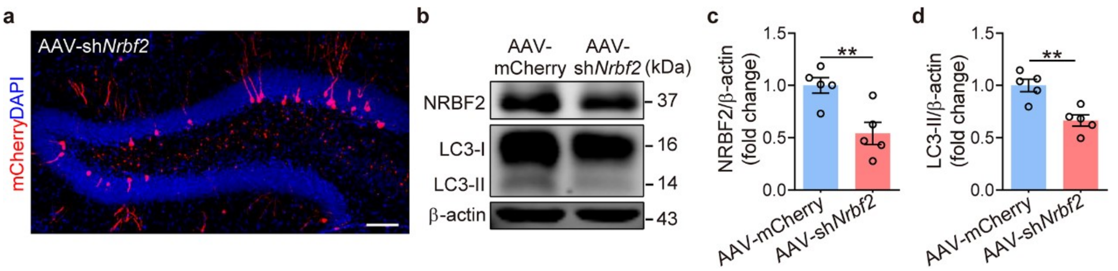

Supplementary Fig. S10 Conditional knockdown of NRBF2 in the DG of Nestin-CreER^{T2} mice.

a Representative image of mCherry^{+} cells in the DG of Nestin-CreER^{T2} mice that treated with AAV-sh*Nrbf2*. Scale bars, 100 μm.

b-d Western blotting analysis (**b**) and quantifications of NRBF2 (**c**) and LC3-II (**d**) protein expression in the DG (n = 5 mice per group).

Data are presented as mean ± SEM and analyzed by two-sided unpaired t test (**c**, **d**). **P < 0.01.

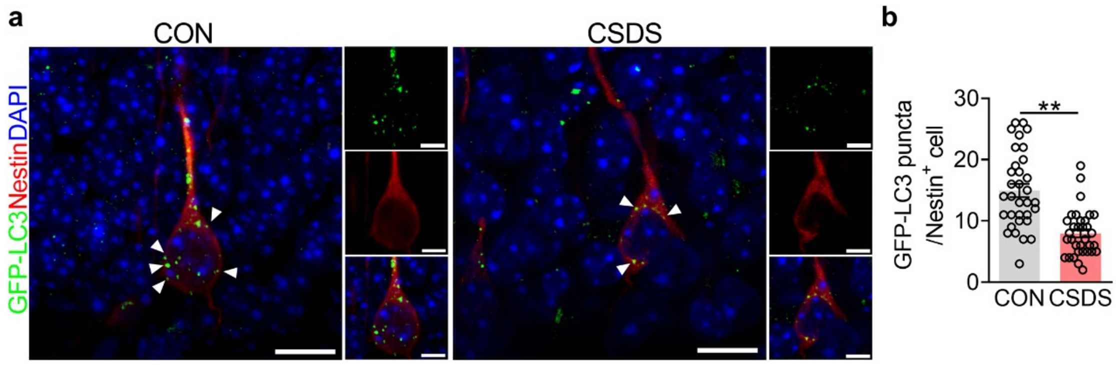

Supplementary Fig. S11 Chronic stress leads to the autophagy deficiency of aNSCs in the DG of CSDS-exposed mice.

a, b Representative images of (**a**) and quantifications of GFP-LC3 puncta within the soma of Nestin^{+} cells in the DG ($n = 33$ cells per group). Nestin (red) and DAPI (blue). Scale bars, 10 μm (overview) and 5 μm (zoom).

Data are presented as mean ± SEM and analyzed by two-sided unpaired t test (**b**). $^{**}P < 0.01$.

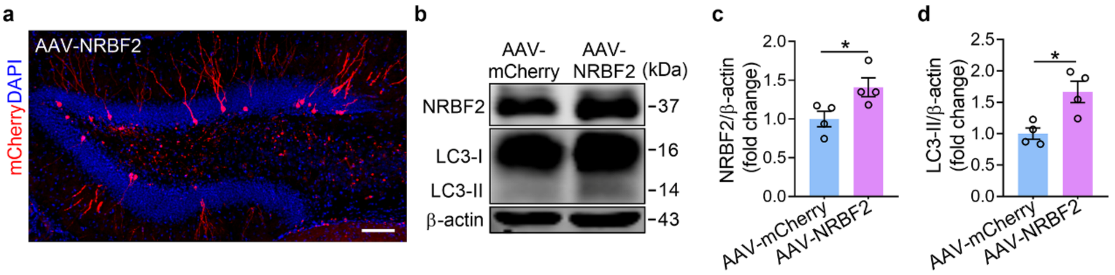

Supplementary Fig. S12 Conditional overexpression of NRBF2 in the DG of Nestin-CreER^{T2} mice.

a Representative image of mCherry^{+} cells in the DG of mice that treated with AAV-NRBF2. Scale bars, 100 μm.

b-d Western blotting analysis (**b**) and quantifications of NRBF2 (**c**) and LC3-II (**d**) protein expression in the DG (n = 4 mice per group).

Data are presented as mean ± SEM and analyzed by two-sided unpaired t test (**c**, **d**). *$P < 0.05$.

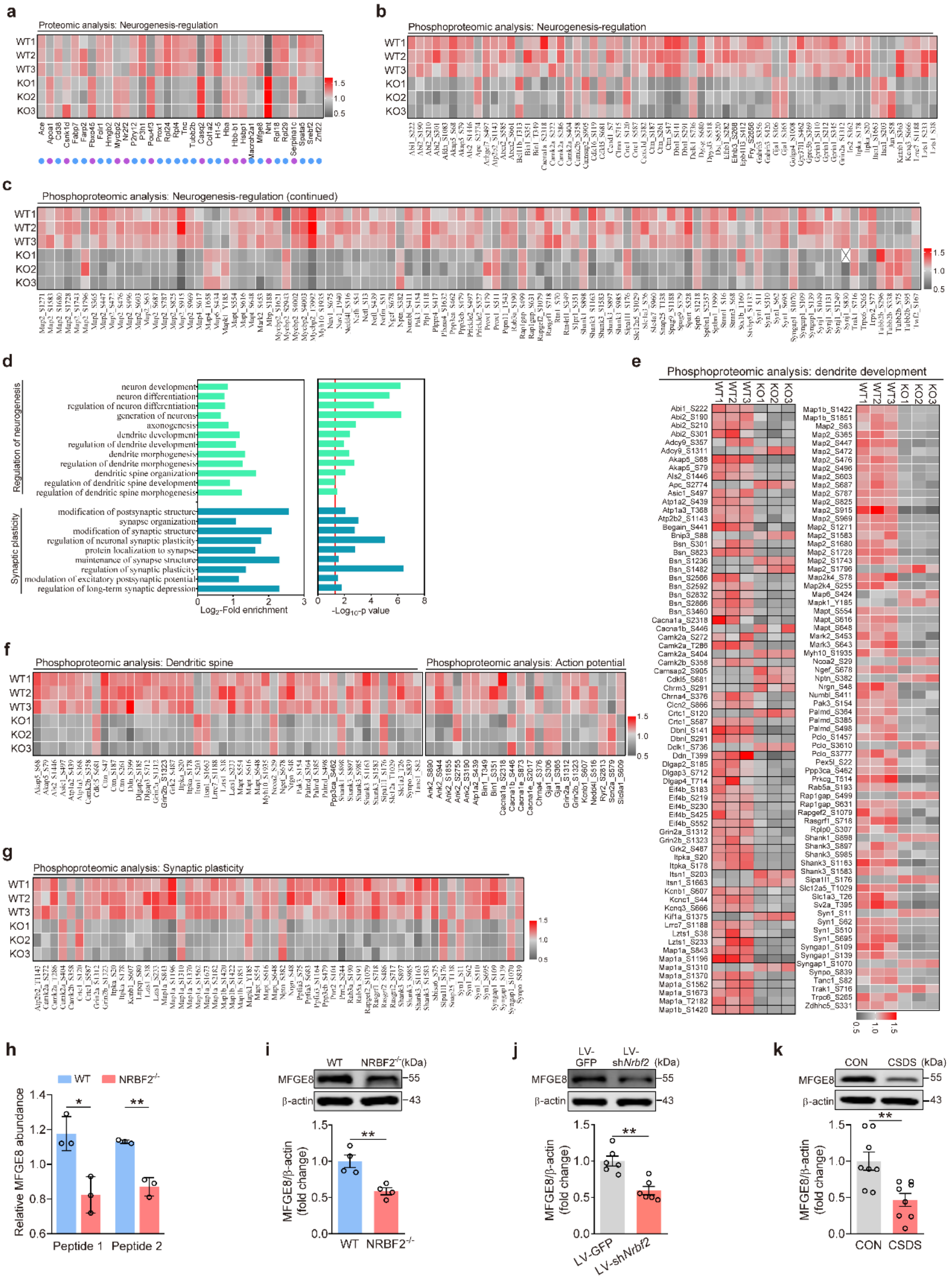

Supplementary Fig. S13 Deletion of NRBF2 in the DG alters the expression or phosphorylation of proteins involved in different biological processes.

a Knockout (KO) of NRBF2 altered the expression of several proteins in the DG involved in the biological processes of neurogenesis in proteomic analysis (n = 3 mice per group).

b, c NRBF2 deletion altered the phosphorylation of some proteins involved in the biological process of neurogenesis in phosphoproteomic analysis (n = 3 mice per group).

d In bioinformatics analysis, the closely related biological processes were combined to better

indicate the alteration of biological processes by NRBF2 deletion, especially, the regulation of neurogenesis or synapse plasticity. **e** NRBF2 deletion altered the phosphorylation of many proteins involved in biological processes of dendritic development (*n* = 3 mice per group).

f, g NRBF2 deletion altered the phosphorylation of many proteins involved in biological processes of dendritic spine (**f**, left), action potential (**f**, right) and synaptic plasticity (**g**) (*n* = 3 mice per group).

h Parallel reaction monitoring proteomics verified the reduce abundance of MFGE8, including peptide 1 and peptide 2, in the DG of $NRBF2^{-/-}$ mice (*n* = 3 mice per group). Peptide 1: FELLGCELHGCSEPLGLK; Peptide 2: QVTGIITQGAR.

i Western blotting analysis and quantification of MFGE8 protein expression in the DG of WT and $NRBF2^{-/-}$ mice (*n* = 4 mice per group).

j Western blotting analysis and quantification of MFGE8 protein expression in control and NRBF2-deficient aNSCs (*n* = 6 independent experiments per group).

k Western blotting analysis and quantification of MFGE8 protein expression in the DG of control and CSDS-exposed mice (*n* = 8 mice per group).

Data are presented as mean ± SEM and analyzed by two-sided unpaired t test (**h**-**k**). $^{*}P < 0.05$ and $^{**}P < 0.01$.

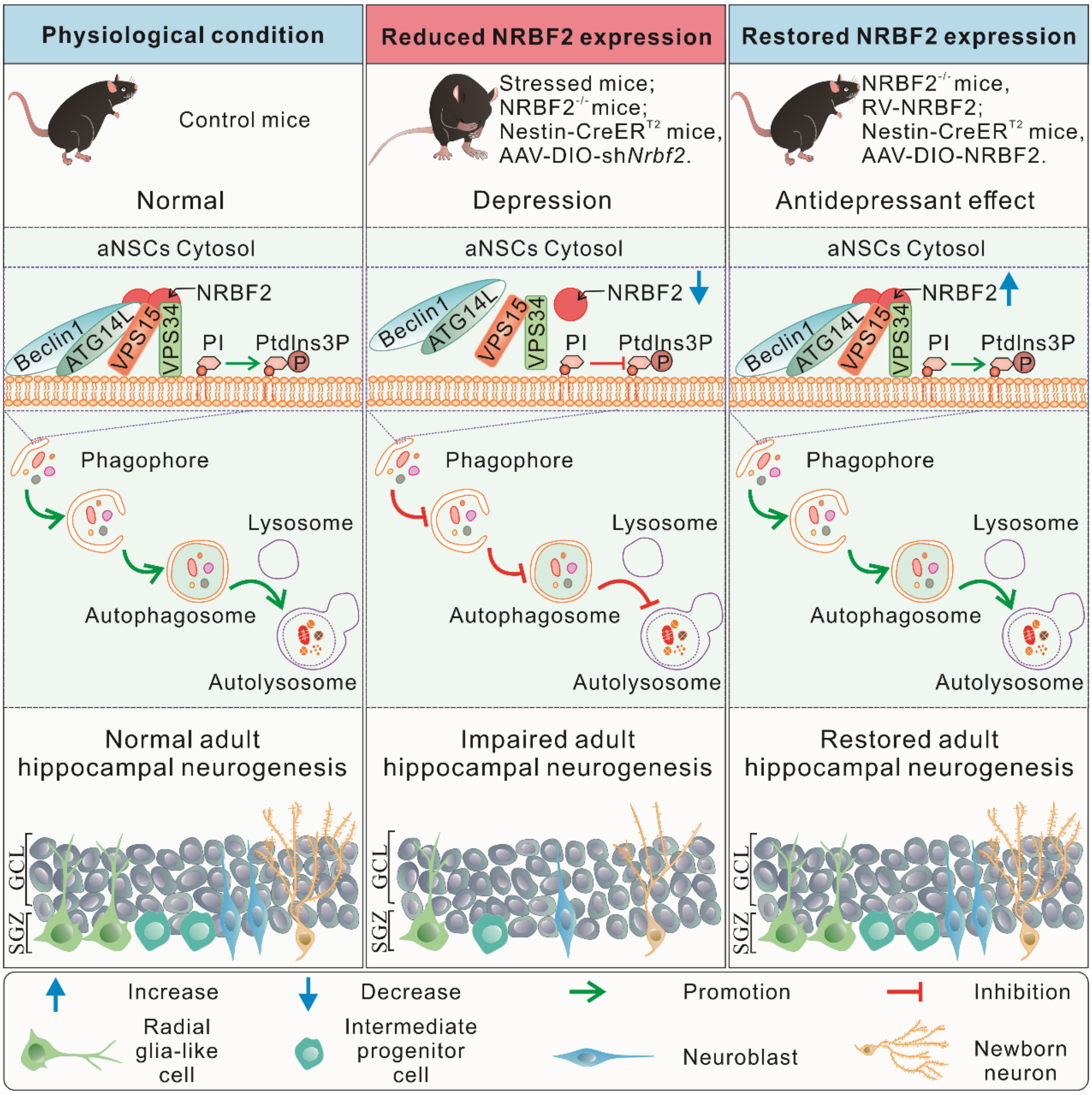

Supplementary Fig. S14 Schematic representation of the aNSCs-specific mechanism of NRBF2 in mediating the impairment of AHN in chronic stress-induced depression.

The expression of NRBF2 is enriched in the aNSCs and their progeny of dentate gyrus, a hippocampal neurogenic niche. Under normal condition, NRBF2-ATG14L interaction mediates the binding with other components in the cytosol and consequent assembly of activated Ptdlns3K complex at the phagophore, synthesizing Ptdlns3P by using phosphatidylinositol (PI), resulting in autophagy induction. Chronic stress induces the decrease of NRBF2 in the dentate gyrus. The deficiency of NRBF2 suppresses the Ptdlns3K complex activity and autophagosome information in aNSCs, disrupts the neurogenesis-related protein network, and finally leads to the exhaustion of the aNSCs pool and developmental defects of newborn neurons, which induces depression-like behavior in mice. Importantly, restoring NRBF2 expression in DG aNSCs increases autophagic flux and rescues AHN deficits of NRBF2$^{-/-}$ mice and CSDS-exposed mice.

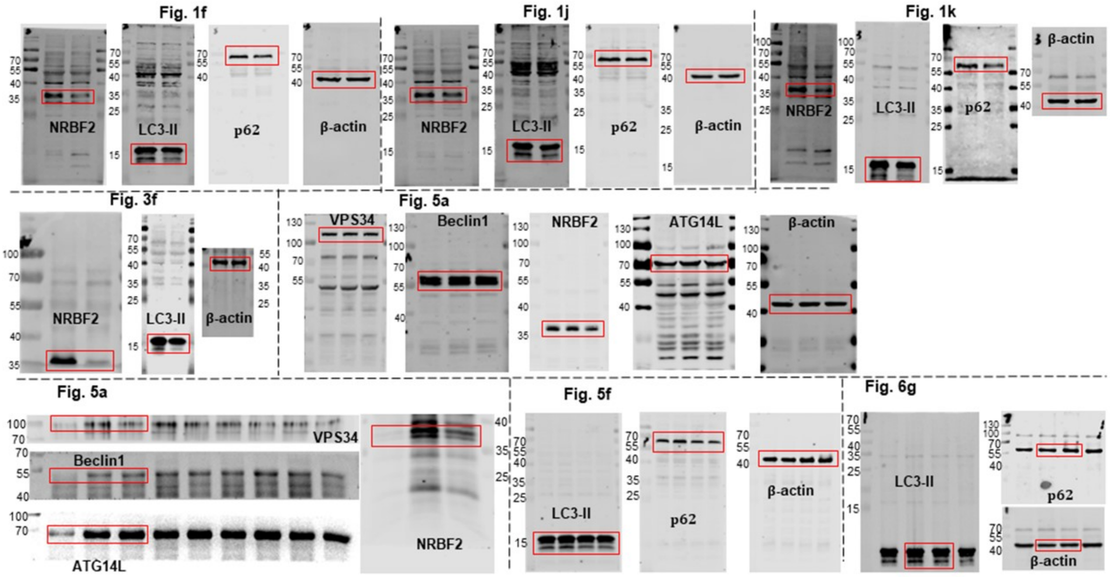

Supplementary Fig. S15 Original blots for Fig. 1f, 1j, 1k, 3f, 5a, 5f, and 6g.

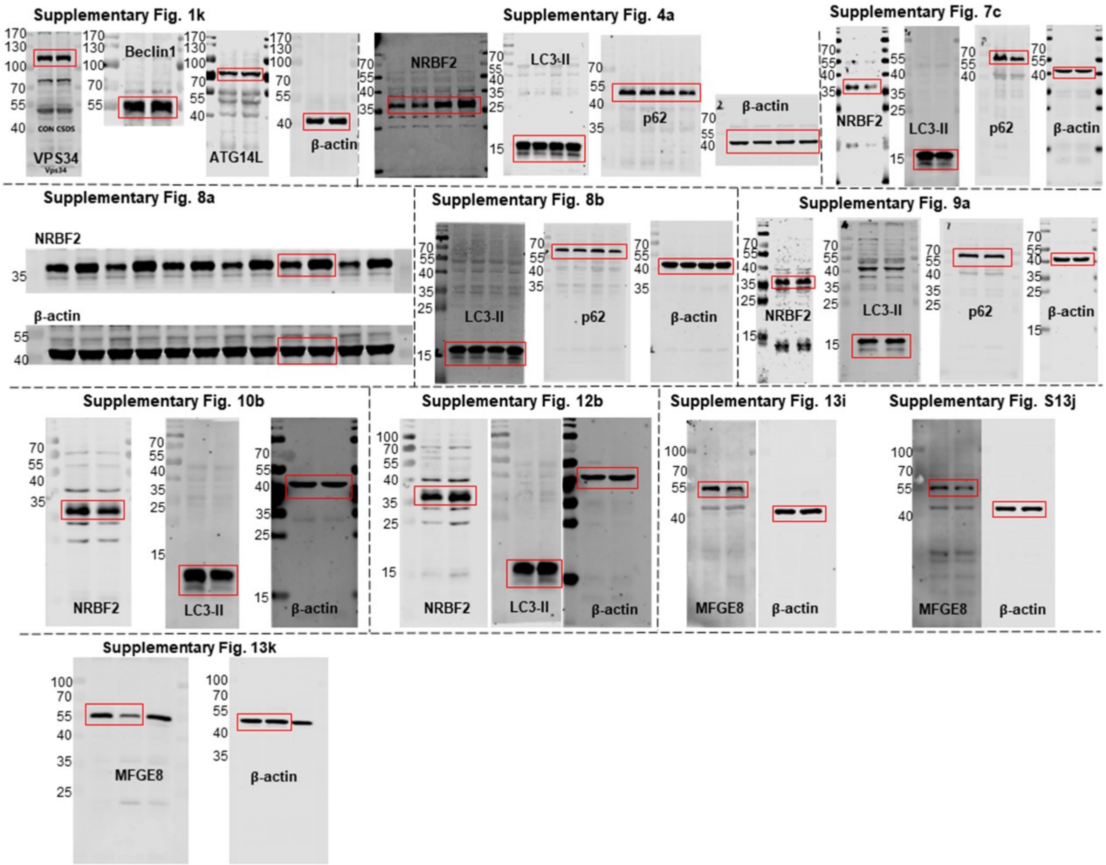

Supplementary Fig. S16. Original blots for Fig. S1k, S4a, S7c, S8a, S8b, S9a, S10b, S12b, S13i, S13j, and S13k.

Supplementary Table

Supplementary Table S1. Primers for qPCR analyses.

Gene	Primer sequences	
	Forward	Reverse
Nrbf2	AAGGACCCCTCAACCTTGCT	CAGTTCCAGTGATAAGTGAGCC
p62	AGGATGGGGACTTGGTTGC	TCACAGATCACATTGGGGTGC
Hbb-b1	GCACCTGACTGATGCTGAGAA	TTCATCGGCGTTCACCTTTCC
Nnt	CTCAGGCTTGTGCTGTGTTG	CTCGGCCATGCAGGTAAGAA
Pou4f3	ATGCGCCGAGTTTGTCTCC	GGGCTTGAACGGATGGTTCT
Apoa1	GGCACGTATGGCAGCAAGAT	CCAAGGAGGAGGATTCAAACTG
Mfge8	AGATGCGGGTATCAGGTGTGA	GGGGCTCAGAACATCCGTG
Fabp7	GGACACAATGCACATTCAAGAAC	CCGAACCACAGACTTACAGTTT
Farp2	GTTATTTGACATCGAGCCGAAGT	GAACTCCAGACCAAAGTAGTCAC
P3h1	ACACAAGCTATTGAATGTGCCA	TTCCCGTTCAGACTTCTGTTTC
Gapdh	AACGACCCCTTCATTGAC	TCCACGACATACTCAGCAC

Supplementary Table S2. List of antibodies

Antibodies	Species	dilution	Supplier
Anti-ATG14L	Rabbit	1:1000 (WB)	MBL, Cat# PD026
Anti-β-actin	Mouse	1:3000 (WB)	Santa Cruz, Cat# sc-517582
Anti-Beclin1	Rabbit	1:1000 (WB)	Novus, Cat# NB110-87318SS
Anti-BrdU	Rat	1:1000 (WB)	Abcam, Cat# ab6126
Anti-cleaved Caspase3	Rabbit	1:500 (IF)	Cell Signaling, Cat# 9661
Anti-DCX	Rabbit	1:400 (IF)	Cell Signaling, Cat# 4604
Anti-GFAP	Mouse	1:400 (IF)	Cell Signaling, Cat# 3670
Anti-GFAP	Rabbit	1:200 (IF)	Proteintech, Cat# 16825-1-AP
Anti-LC3B	Rabbit	1:1000 (WB)	Cell Signaling, Cat# 2775
Anti-LC3	Rabbit	1:200 (IF)	MBL, Cat# PM036
Anti-MFGE8	Mouse	1:1000 (WB)	Santa Cruz, Cat# sc-33546
Anti-Nestin	Chicken	1:500 (JF)	Aves Labs, Cat# AB_2314882
Anti-NeuN	Rabbit	1:500 (IF)	Abcam, Cat# ab177487
Anti-NRBF2	Rabbit	1:1000 (WB)	Cell Signaling, Cat# 8066
Anti-NRBF2	Mouse	1:200 (IF)	Santa Cruz, Cat# sc-365213
Anti-RFP	Rabbit	1:200 (IF)	Abcam, Cat# ab62341
Anti-Sox2	Goat	1:200 (IF)	R&D Systems. Cat# AF2018
Anti-SQSTM1/p62	Mouse	1:1000 (WB)	Abcam, Cat# ab56416
Anti-SQSTM1/p62	Mouse	1:200 (IF)	Abcam, Cat# ab56416
Anti-Tbr2	Rabbit	1:200 (IF)	Abcam, Cat# ab23345
Anti-Tuj1	Mouse	1:500 (IF)	Proteintech, Cat# 66375-1-Ig
Anti-VPS34	Rabbit	1:1000 (WB)	Proteintech, Cat# 12452-1-AP
Alexa fluor-405-anti-Rat secondary antibody	Donkey	1:1000 (IF)	Invitrogen, Cat# A48261
Alexa fluor-488-anti-Rat secondary antibody	Donkey	1:1000 (IF)	Invitrogen, Cat#A21208
Alexa fluor-488-anti-Rabbit secondary antibody	Donkey	1:1000 (IF)	Invitrogen, Cat#A21206
Alexa fluor-647-anti-Rabbit secondary antibody	Donkey	1:1000 (IF)	Invitrogen, Cat#A31573
Alexa fluor-594-anti-Chicken secondary antibody	Donkey	1:1000 (IF)	Jackson ImmunoResearch, Cat# 703-585-155
Alexa fluor-594-anti-Rabbit secondary antibody	Donkey	1:1000 (IF)	Invitrogen, Cat# A21207
Alexa fluor-594-anti-Goat secondary antibody	Donkey	1:1000 (IF)	Invitrogen, Cat# A-11058
Alexa fluor-594-anti-Mouse secondary antibody	Donkey	1:1000 (IF)	Invitrogen, Cat# A21203
Alexa fluor-647-anti-Mouse secondary antibody	Donkey	1:1000 (IF)	Invitrogen, Cat#A31571

IRDye® LI-COR 800CW anti-Mouse IgG (H + L)	Goat	1:20000 (WB)	LI-COR Biosciences, Cat# 926-32352
IRDye® 800CW anti-Rabbit IgG (H + L)	Goat	1:20000 (WB)	LI-COR Biosciences, Cat# 925-32211

Supplementary Table S3. The statistical information about this study.

**Figures and numbers of animals or cells**	**Statistical analysis**	**Post hoc tests**	**mean ± SEM**
1b: Number of AVs in DG in CSDS model<br>CON (*n* = 22)<br>CSDS (*n* = 18)	Unpaired t test<br>t = 4.612, $P < 0.001$		CON 2.727 ± 0.256<br>CSDS 1.333 ± 0.114
1d: LC3 puncta in DG in CSDS model<br>CON (*n* = 35)<br>CSDS (*n* = 38)	Unpaired t test<br>t = 7.744, $P < 0.001$		CON 12.486 ± 0.850<br>CSDS 5.316 ± 0.420
1e: *Nrbf2* mRNA level in DG in CSDS model<br>CON (*n* = 11)<br>CSDS (*n* = 13)	Unpaired t test<br>t = 4.761, $P < 0.001$		CON 1.014 ± 0.054<br>CSDS 0.708 ± 0.038
1g: NRBF2 protein expression in DG in CSDS model<br>CON (*n* = 14)<br>CSDS (*n* = 14)	Unpaired t test<br>t = 4.991, $P < 0.001$		CON 1.000 ± 0.065<br>CSDS 0.594 ± 0.049
1h: LC3-II protein expression in DG in CSDS model<br>CON (*n* = 14)<br>CSDS (*n* = 14)	Unpaired t test<br>t = 5.180, $P < 0.001$		CON 1.000 ± 0.046<br>CSDS 0.599 ± 0.062
1i: p62 protein expression in DG in CSDS model<br>CON (*n* = 14)<br>CSDS (*n* = 14)	Unpaired t test<br>t = 3.288, $P = 0.003$		CON 1.000 ± 0.061<br>CSDS 0.743 ± 0.049
1j: protein expression in DG in CUS model<br>CON (*n* = 13)<br>CUS (*n* = 13)	NRBF2: Unpaired t test, t = 6.710, $P < 0.001$<br><br>LC3-II: Unpaired t test, t = 3.560, $P = 0.002$<br><br>p62: Unpaired t test, t = 3.343, $P = 0.003$		CON 1.000 ± 0.045<br>CUS 0.654 ± 0.031<br><br>CON 1.000 ± 0.090<br>CUS 0.643 ± 0.045<br><br>CON 1.000 ± 0.042<br>CUS 0.776 ± 0.052

1k: protein expression in DG in CORT model<br>CON (n = 9)<br>CORT (n = 11)	NRBF2: Unpaired t test, t = 3.554, P = 0.003<br><br>LC3-II: Unpaired t test, t = 3.116, P = 0.006<br><br>p62: Unpaired t test, t = 2.298, P = 0.034		CON 1.000 ± 0.090<br>CORT 0.646 ± 0.052<br><br>CON 1.000 ± 0.113<br>CORT 0.625 ± 0.058<br><br>CON 1.000 ± 0.135<br>CUS 0.687 ± 0.056
2b. $BrdU^+Sox2^+GFAP^+$ RGLs after virus injection<br>CON + LV-vector (n = 3)<br>CON + LV-NRBF2 (n = 3)<br>CSDS + LV-vector (n = 3)<br>CSDS + LV-NRBF2 (n = 3)<br><br>$BrdU^+DCX^+$ neuroblasts after virus injection<br>CON + LV-vector (n = 4)<br>CON + LV-NRBF2 (n = 4)<br>CSDS + LV-vector (n = 5)<br>CSDS + LV-NRBF2 (n = 5)<br><br>$BrdU^+NeuN^+$ neurons after virus injection<br>CON + LV-vector (n = 3)	Two-way ANOVA:<br>Stress: F (1, 8) = 6.819, P = 0.031<br>Virus: F (1, 8) = 48.820, P < 0.001<br>Stress × Virus: F (1, 8) = 4.483, P = 0.067<br><br>Two-way ANOVA:<br>Stress: F (1, 14) = 19.220, P = 0.001<br>Virus: F (1, 14) = 37.860, P < 0.001<br>Stress × Virus: F (1, 14) = 12.150, P = 0.004<br><br>Two-way ANOVA:<br>Stress: F (1, 8) = 16.000, P = 0.004<br>Virus: F (1, 8) =	Bonferroni's post hoc test<br>CON + LV-vector vs CSDS + LV-vector, P < 0.001; CON + LV-NRBF2 vs CSDS + LV-NRBF2, P = 0.018; CON + LV-vector vs CON + LV-NRBF2, P > 0.999; CSDS + LV-vector vs CSDS + LV-NRBF2, P = 0.020<br><br>Bonferroni's post hoc test<br>CON + LV-vector vs CSDS + LV-vector, P < 0.001; CON + LV-NRBF2 vs CSDS + LV-NRBF2, P = 0.160; CON + LV-vector vs CON + LV-NRBF2, P = 0.803; CSDS + LV-vector vs CSDS + LV-NRBF2, P < 0.001<br><br>Bonferroni's post hoc test<br>CON + LV-vector vs CSDS + LV-	CON + LV-vector 1495.0 ± 72.111<br>CON + LV-NRBF2 1530.0 ± 73.993<br>CSDS + LV-vector 850.0 ± 49.244<br>CSDS + LV-NRBF2 1185.0 ±83.516<br><br>CON + LV-vector 1102.5 ± 73.072<br>CON + LV-NRBF2 1152.5 ± 41.105<br>CSDS + LV-vector 566.0 ± 25.612<br>CSDS + LV-NRBF2 1004.0 ± 69.036<br><br>CON + LV-vector 1135.0 ± 39.051<br>CON + LV-NRBF2 1125.0 ± 31.225

CON + LV-NRBF2 ($n = 3$)<br>CSDS + LV-vector ($n = 3$)<br>CSDS + LV-NRBF2 ($n = 3$)	172.800, $P < 0.001$<br>Stress × Virus: F (1, 8) = 18.100, $P = 0.003$	vector, $P < 0.001$; CON + LV-NRBF2 vs CSDS + LV-NRBF2, $P = 0.001$; CON + LV-vector vs CON + LV-NRBF2, $P > 0.999$; CSDS + LV-vector vs CSDS + LV-NRBF2, $P = 0.001$	CSDS + LV-vector 450.0 ± 52.678<br>CSDS + LV-NRBF2 775.0 ± 30.414
2d: RFP-LC3 puncta in the DG after NRBF2 overexpression<br>CON + LV-vector ($n = 36$)<br>CON + LV-NRBF2 ($n = 35$)<br>CSDS + LV-vector ($n = 35$)<br>CSDS + LV-NRBF2 ($n = 32$)	Two-way ANOVA<br>Stress: F (1, 134) = 11.030, $P = 0.001$<br>Virus: F (1, 134) = 9.814, $P = 0.002$<br>Stress × virus: F (1, 134) = 0.631, $P = 0.428$	Bonferroni's post hoc test<br>CON + LV-vector vs CSDS + LV-vector, $P = 0.011$; CON + LV-NRBF2 vs CSDS + LV-NRBF2, $P = 0.211$; CON + LV-vector vs CON + LV-NRBF2, $P = 0.144$; CSDS + LV-vector vs CSDS + LV-NRBF2, $P = 0.010$	CON + LV-vector 14.944 ± 1.051<br>CON + LV-NRBF2 17.371 ± 0.918<br>CSDS + LV-vector 11.171 ± 0.709<br>CSDS + LV-NRBF2 15.125 ± 1.129
2g. Total dendritic length after virus injection<br>CON + LV-vector ($n = 29$)<br>CON + LV-NRBF2 ($n = 29$)<br>CSDS + LV-vector ($n = 33$)<br>CSDS + LV-NRBF2 ($n = 30$)	Two-way ANOVA:<br>Stress: F (1, 117) = 22.970, $P < 0.001$<br>Virus: F (1, 117) = 53.260, $P < 0.001$<br>Stress × virus: F (1, 117) = 16.070, $P < 0.001$	Bonferroni's post hoc test<br>CON + LV-vector vs CSDS + LV-vector, $P < 0.001$; CON + LV-NRBF2 vs CSDS + LV-NRBF2, $P = 0.047$; CON + LV-vector vs CON + LV-NRBF2, $P > 0.999$; CSDS + LV-vector vs CSDS + LV-NRBF2, $P < 0.001$	CON + LV-vector 1078.330 ± 35.059<br>CON + LV-NRBF2 1107.072 ± 39.224<br>CSDS + LV-vector 663.663 ± 31.497<br>CSDS + LV-NRBF2 986.461 ± 40.846
2h. Number of crossings after virus injection<br>CON + LV-vector ($n = 29$)<br>CON + LV-NRBF2	Repeated measures ANOVA:<br>Stress: F (1, 60) = 72.700, $P < 0.001$<br>LV-NRBF2: F (1, 61) =		

(*n* = 29)<br>CSDS + LV-vector<br>(*n* = 33)<br>CSDS + LV-NRBF2<br>(*n* = 30)	40.610, *P* < 0.001		
2i. Spine density after virus injection<br>CON + LV-vector<br>(*n* = 33)<br>CON + LV-NRBF2<br>(*n* = 35)<br>CSDS + LV-vector<br>(*n* = 37)<br>CSDS + LV-NRBF2<br>(*n* = 37)	Two-way ANOVA:<br>Stress: F (1, 138) = 2.410, *P* = 0.123<br>Virus: F (1, 138) = 83.130, *P* < 0.001<br>Stress × virus: F (1, 138) = 14.510, *P* < 0.001	Bonferroni's post hoc test<br>CON + LV-vector vs CSDS + LV-vector, *P* < 0.001;<br>CON + LV-NRBF2 vs CSDS + LV-NRBF2, *P* = 0.001;<br>CON + LV-vector vs CON + LV-NRBF2, *P* = 0.241;<br>CSDS + LV-vector vs CSDS + LV-NRBF2, *P* < 0.001	CON + LV-vector<br>22.299 ± 1.455<br>CON + LV-NRBF2<br>19.869 ± 1.187<br>CSDS + LV-vector<br>8.382 ± 0.686<br>CSDS + LV-NRBF2<br>14.154 ± 0.920
2j. SIT after virus injection<br>CON + LV-vector<br>(*n* = 9)<br>CON + LV-NRBF2<br>(*n* = 8)<br>CSDS + LV-vector<br>(*n* = 10)<br>CSDS + LV-NRBF2<br>(*n* = 14)	Two-way ANOVA:<br>Stress: F (1, 37) = 7.532, *P* = 0.009<br>Virus: F (1, 37) = 28.210, *P* < 0.001<br>Stress × virus: F (1, 37) = 4.582, *P* = 0.039	Bonferroni's post hoc test<br>CON + LV-vector vs CSDS + LV-vector, *P* < 0.001;<br>CON + LV-NRBF2 vs CSDS + LV-NRBF2, *P* = 0.057;<br>CON + LV-vector vs CON + LV-NRBF2, *P* > 0.999;<br>CSDS + LV-vector vs CSDS + LV-NRBF2, *P* = 0.001	CON + LV-vector<br>1.165 ± 0.048<br>CON + LV-NRBF2<br>1.226 ± 0.077<br>CSDS + LV-vector<br>0.419 ± 0.061<br>CSDS + LV-NRBF2<br>0.908 ± 0.124
2k. TST after virus injection<br>CON + LV-vector<br>(*n* = 14)<br>CON + LV-NRBF2<br>(*n* = 14)<br>CSDS + LV-vector<br>(*n* = 11)<br>CSDS + LV-NRBF2<br>(*n* = 13)	Two-way ANOVA:<br>Stress: F (1, 48) = 6.600, *P* = 0.013<br>Virus: F (1, 48) = 2.964, *P* = 0.092<br>Stress × virus: F (1, 39) = 11.550, *P* = 0.001	Bonferroni's post hoc test<br>CON + LV-vector vs CSDS + LV-vector, *P* = 0.002<br>CON + LV-NRBF2 vs CSDS + LV-NRBF2, *P* = 0.462<br>CON + LV-vector vs CON + LV-	CON + LV-vector<br>86.737 ± 10. 724<br>CON + LV-NRBF2<br>95.172 ± 8.137<br>CSDS + LV-vector<br>138.788 ± 12.381<br>CSDS + LV-NRBF2<br>78.121 ± 9.478

		NRBF2, $P > 0.999$<br>CSDS + LV-vector vs CSDS + LV-NRBF2, $P < 0.001$	
2l. FST after virus injection<br>CON + LV-vector ($n = 15$)<br>CON + LV-NRBF2 ($n = 15$)<br>CSDS + LV-vector ($n = 12$)<br>CSDS + LV-NRBF2 ($n = 13$)	Two-way ANOVA:<br>Stress: $F(1, 51) = 6.435$, $P = 0.014$<br>Virus: $F(1, 51) = 11.04$, $P = 0.002$<br>Stress × virus: $F(1, 51) = 6.224$, $P = 0.016$	Bonferroni's post hoc test<br>CON + LV-vector vs CSDS + LV-vector, $P < 0.001$; CON + LV-NRBF2 vs CSDS + LV-NRBF2, $P > 0.999$; CON + LV-vector vs CON + LV-NRBF2, $P > 0.999$; CSDS + LV-vector vs CSDS + LV-NRBF2, $P = 0.003$	CON + LV-vector 54.337 ± 8.414<br>CON + LV-NRBF2 53.885 ± 10.399<br>CSDS + LV-vector 117.291 ± 13.438<br>CSDS + LV-NRBF2 62.842 ± 11. 310
3b: $NRBF2^{-/-}$ mice-SPT<br>WT ($n = 10$)<br>$NRBF2^{-/-}$ ($n = 9$)	Unpaired t test<br>$t = 4.268$, $P = 0.001$		WT 89.434 ± 1.972<br>$NRBF2^{-/-}$ 54.874 ± 8.274
3c: $NRBF2^{-/-}$ mice-TST<br>WT ($n = 10$)<br>$NRBF2^{-/-}$ ($n = 8$)	Unpaired t test<br>$t = 2.830$, $P = 0.012$		WT 105.676 ± 11.290<br>$NRBF2^{-/-}$ 162.248 ± 17.395
3d: $NRBF2^{-/-}$ mice-FST<br>WT ($n = 10$)<br>$NRBF2^{-/-}$ ($n = 9$)	Unpaired t test<br>$t = 3.789$, $P = 0.002$		WT 89.083 ± 14.431<br>$NRBF2^{-/-}$ 161.964 ± 12.388
3e: $NRBF2^{-/-}$ mice-OFT: total distance<br>WT ($n = 10$)<br>$NRBF2^{-/-}$ ($n = 9$)	Unpaired t test<br>$t = 0.561$, $P = 0.582$		WT 19.774 ± 1.030<br>$NRBF2^{-/-}$ 18.939 ± 1.074
3g: protein expression in DG of $NRBF2^{-/-}$ mice<br><br>WT ($n = 4$)<br>$NRBF2^{-/-}$ ($n = 4$)	NRBF2: unpaired t test<br>$t = 14.140$, $P < 0.001$<br><br>LC3-II: unpaired t test<br>$t = 6.202$, $P = 0.001$		WT 1.000 ± 0.067<br>$NRBF2^{-/-}$ 0.053 ± 0.007<br><br>WT 1.000 ± 0.063<br>$NRBF2^{-/-}$ 0.512 ± 0.047
3i: LC3 puncta within $Nestin^{+}$ cells in DG of $NRBF2^{-/-}$ mice<br>WT ($n = 25$)<br>$NRBF2^{-/-}$ ($n = 29$)	Unpaired t test<br>$t = 5.295$, $P < 0.001$		WT 12.840 ± 0.984<br>$NRBF2^{-/-}$ 7.069 ± 0.552

3l: $BrdU^+Sox2^+GFAP^+$ RGLs of $NRBF2^{-/-}$ mice<br>WT (*n* = 5)<br>$NRBF2^{-/-}$ (*n* = 5)<br><br>$BrdU^+Tbr2^+$ IPCs of $NRBF2^{-/-}$ mice<br>WT (*n* = 5)<br>$NRBF2^{-/-}$ (*n* = 5)<br><br>$BrdU^+DCX^+$ neuroblasts of $NRBF2^{-/-}$ mice<br>WT (*n* = 5)<br>$NRBF2^{-/-}$ (*n* = 5)	Unpaired t test<br>t = 6.305, *P* = 0.001<br><br><br><br>Unpaired t test<br>t = 5.972, *P* = 0.001<br><br><br><br>Unpaired t test<br>t = 6.511, *P* = 0.001		WT 1398.0 ± 82.89<br>$NRBF2^{-/-}$ 783.0 ± 51.40<br><br><br><br>WT 1326.0 ± 98.32<br>$NRBF2^{-/-}$ 672.0 ± 48.23<br><br><br><br>WT 1190.0 ± 86.96<br>$NRBF2^{-/-}$ 513.0 ± 57.00
3o: $BrdU^+NeuN^+$ neurons of $NRBF2^{-/-}$ mice<br>WT (*n* = 5)<br>$NRBF2^{-/-}$ (*n* = 5)	Unpaired t test<br>t = 13.220, *P* < 0.001		WT 1023.0 ± 45.349<br>$NRBF2^{-/-}$ 336.0 ± 25.367
4b: cOE-SPT<br>WT + RV-mCherry (*n* = 10)<br>WT + RV-NRBF2 (*n* = 10)<br>$NRBF2^{-/-}$ + RV-mCherry (*n* = 10)<br>$NRBF2^{-/-}$ + RV-NRBF2 (*n* = 10)<br><br>cOE-TST:<br>WT + RV-mCherry (*n* = 10)<br>WT + RV-NRBF2 (*n* = 10)<br>$NRBF2^{-/-}$ + RV-mCherry (*n* = 10)<br>$NRBF2^{-/-}$ + RV-NRBF2	Two-way ANOVA<br>$NRBF2^{-/-}$: F (1, 36) = 8.806, *P* = 0.005<br>RV-NRBF2: F (1, 36) = 15.520, *P* < 0.001<br>$NRBF2^{-/-}$ × RV-NRBF2: F (1, 36) = 13.210, *P* = 0.001<br><br>Two-way ANOVA<br>$NRBF2^{-/-}$: F (1, 36) = 15.090, *P* = 0.001<br>RV-NRBF2: F (1, 36) = 1.886, *P* = 0.178<br>$NRBF2^{-/-}$ × RV-NRBF2: F (1, 36) = 20.700, *P* < 0.001	Bonferroni's post hoc test<br>WT + RV-mCherry vs $NRBF2^{-/-}$ + RV-mCherry, *P* < 0.001;<br>WT + RV-NRBF2 vs $NRBF2^{-/-}$ + RV-NRBF2, *P* > 0.999;<br>WT + RV-mCherry vs WT + RV-NRBF2, *P* > 0.999;<br>$NRBF2^{-/-}$ + RV-mCherry vs $NRBF2^{-/-}$ + RV-NRBF2, *P* < 0.001<br><br>Bonferroni's post hoc test<br>WT + RV-mCherry vs $NRBF2^{-/-}$ + RV-mCherry, *P* = 0.001;<br>WT + RV-NRBF2 vs $NRBF2^{-/-}$ + RV-NRBF2, *P* = 0.062;	WT + RV-mCherry<br>91.497 ± 0.757<br>WT + RV-NRBF2<br>88.593 ± 2.070<br>$NRBF2^{-/-}$ + RV-mCherry<br>58.549 ± 7.865<br>$NRBF2^{-/-}$ + RV-NRBF2<br>87.268 ± 2.994<br><br>WT + RV-mCherry<br>91.461 ± 17.229<br>WT + RV-NRBF2<br>99.368 ± 10.070<br>$NRBF2^{-/-}$ + RV-mCherry<br>161.772 ± 10.889<br>$NRBF2^{-/-}$ + RV-NRBF2

(n = 10)<br><br>cOE-FST:<br>WT + RV-mCherry<br>(n = 10)<br>WT + RV-NRBF2<br>(n = 10)<br>$NRBF2^{-/-}$ + RV-mCherry<br>(n = 10)<br>$NRBF2^{-/-}$ + RV-NRBF2<br>(n = 10)	 <br>Two-way ANOVA<br>$NRBF2^{-/-}$: F (1, 36) = 9.499, P = 0.004<br>RV-NRBF2: F (1, 36) = 50.340, P < 0.001<br>$NRBF2^{-/-}$ × RV-NRBF2: F (1, 36) = 2.404, P = 0.130	WT + RV-mCherry vs WT + RV-NRBF2, P > 0.999; $NRBF2^{-/-}$ + RV-mCherry vs $NRBF2^{-/-}$ + RV-NRBF2, P < 0.001<br><br>Bonferroni's post hoc test<br>WT + RV-mCherry vs $NRBF2^{-/-}$ + RV-mCherry, P < 0.001; WT + RV-NRBF2 vs $NRBF2^{-/-}$ + RV-NRBF2, P = 0.001; WT + RV-mCherry vs WT + RV-NRBF2, P = 0.572; $NRBF2^{-/-}$ + RV-mCherry vs $NRBF2^{-/-}$ + RV-NRBF2, P = 0.005	61.662 ± 6.834<br><br>WT + RV-mCherry<br>57.802 ± 13.554<br>WT + RV-NRBF2<br>35.587 ± 9.896<br>$NRBF2^{-/-}$ + RV-mCherry<br>183.198 ± 15.399<br>$NRBF2^{-/-}$ + RV-NRBF2<br>116.006 ± 17.967
4d: $BrdU^+Sox2^+GFAP^+$ RGLs of $NRBF2^{-/-}$ mice<br>WT + RV-mCherry<br>(n = 4)<br>WT + RV-NRBF2<br>(n = 4)<br>$NRBF2^{-/-}$ + RV-mCherry<br>(n = 4)<br>$NRBF2^{-/-}$ + RV-NRBF2<br>(n = 4)<br><br>$BrdU^+DCX^+$ neuroblasts of $NRBF2^{-/-}$ mice<br>WT + RV-mCherry<br>(n = 4)	Two-way ANOVA<br>$NRBF2^{-/-}$: F (1, 12) = 12.690, P = 0.004<br>RV-NRBF2: F (1, 12) = 50.760, P < 0.001<br>$NRBF2^{-/-}$ × RV-NRBF2: F (1, 12) = 23.310, P < 0.001<br><br>Two-way ANOVA<br>$NRBF2^{-/-}$: F (1, 12) = 13.430, P = 0.003<br>RV-NRBF2: F (1, 12) =	Bonferroni's post hoc test<br>WT + RV-mCherry vs $NRBF2^{-/-}$ + RV-mCherry, P < 0.001; WT + RV-NRBF2 vs $NRBF2^{-/-}$ + RV-NRBF2, P = 0.261; WT + RV-mCherry vs WT + RV-NRBF2, P = 0.777; $NRBF2^{-/-}$ + RV-mCherry vs $NRBF2^{-/-}$ + RV-NRBF2, P < 0.001<br><br>Bonferroni's post hoc test<br>WT + RV-mCherry vs $NRBF2^{-/-}$ + RV-	WT + RV-mCherry<br>1522.500 ± 102.926<br>WT + RV-NRBF2<br>1421.250 ± 58.537<br>$NRBF2^{-/-}$ + RV-mCherry<br>566.250 ± 63.159<br>$NRBF2^{-/-}$ + RV-NRBF2<br>1237.500 ± 87.142<br><br>WT + RV-mCherry<br>1327.500 ± 67.222<br>WT + RV-NRBF2<br>1380.000 ± 125.946

$NRBF2^{-/-}$ + RV-mCherry (n = 4)<br>WT + RV-NRBF2 (n = 4)<br>$NRBF2^{-/-}$ + RV-NRBF2 (n = 4)	17.330, $P = 0.001$<br>$NRBF2^{-/-}$ × RV-NRBF2: $F(1, 12) = 9.779$, $P = 0.009$	mCherry, $P = 0.001$<br>WT + RV-NRBF2 vs $NRBF2^{-/-}$ + RV-NRBF2, $P = 0.956$<br>WT + RV-mCherry vs WT + RV-NRBF2, $P > 0.999$<br>$NRBF2^{-/-}$ + RV-mCherry vs $NRBF2^{-/-}$ + RV-NRBF2, $P = 0.001$	$NRBF2^{-/-}$ + RV-mCherry<br>615.000 ± 50.867<br>$NRBF2^{-/-}$ + RV-NRBF2<br>1278.750 ± 123.447
$BrdU^{+}NeuN^{+}$ neurons of $NRBF2^{-/-}$ mice<br>WT + RV-mCherry (n = 4)<br>$NRBF2^{-/-}$ + RV-mCherry (n = 4)<br>WT + RV-NRBF2 (n = 4)<br>$NRBF2^{-/-}$ + RV-NRBF2 (n = 4)	Two-way ANOVA<br>$NRBF2^{-/-}$: $F(1, 12) = 6.216$, $P = 0.028$<br>RV-NRBF2: $F(1, 12) = 41.250$, $P < 0.001$<br>$NRBF2^{-/-}$ × RV-NRBF2: $F(1, 12) = 6.381$, $P = 0.027$	Bonferroni's post hoc test<br>WT + RV-mCherry vs $NRBF2^{-/-}$ + RV-mCherry, $P < 0.001$;<br>WT + RV-NRBF2 vs $NRBF2^{-/-}$ + RV-NRBF2, $P = 0.035$;<br>WT + RV-mCherry vs WT + RV-NRBF2, $P > 0.999$;<br>$NRBF2^{-/-}$ + RV-mCherry vs $NRBF2^{-/-}$ + RV-NRBF2, $P = 0.008$	WT + RV-mCherry<br>1065.000 ± 67.639<br>WT + RV-NRBF2<br>1062.500 ± 61.695<br>$NRBF2^{-/-}$ + RV-mCherry<br>387.500 ± 56.771<br>$NRBF2^{-/-}$ + RV-NRBF2<br>767.500 ± 106.409
4g: Dendritic length of newborn neurons<br>WT + RV-mCherry (n = 49)<br>WT + RV-NRBF2 (n = 44)<br>$NRBF2^{-/-}$ + RV-mCherry (n = 41)<br>$NRBF2^{-/-}$ + RV-NRBF2 (n = 45)	Two-way ANOVA<br>$NRBF2^{-/-}$: $F(1, 175) = 127.700$, $P < 0.001$<br>RV-NRBF2: $F(1, 175) = 103.700$, $P < 0.001$<br>$NRBF2^{-/-}$ × RV-NRBF2: $F(1, 175) = 11.640$, $P = 0.001$	Bonferroni's post hoc test<br>WT + RV-mCherry vs $NRBF2^{-/-}$ + RV-mCherry, $P < 0.001$;<br>$NRBF2^{-/-}$ + RV-mCherry vs $NRBF2^{-/-}$ + RV-NRBF2, $P < 0.001$	WT + RV-mCherry<br>968.827 ± 24.809<br>WT + RV-NRBF2<br>1192.521 ± 24.809<br>$NRBF2^{-/-}$ + RV-mCherry<br>632.262 ± 24.104<br>$NRBF2^{-/-}$ + RV-NRBF2<br>1015.899 ± 29.630
4h: Number of crossings<br>WT + RV-mCherry (n = 49)<br>WT + RV-NRBF2 (n = 44)<br>$NRBF2^{-/-}$ + RV-mCherry	Repeated measures ANOVA<br>$NRBF2^{-/-}$: $F(1, 88) = 100.800$, $P < 0.001$<br>RV-NRBF2: $F(1, 84) = 89.760$, $P < 0.001$		

(n = 41)<br>NRBF2$^{-/-}$ + RV-NRBF2<br>(n = 45)			
4i: Spine density of newborn neurons<br>WT + RV-mCherry<br>(n = 46)<br>WT + RV-NRBF2<br>(n = 45)<br>NRBF2$^{-/-}$ + RV-mCherry<br>(n = 44)<br>NRBF2$^{-/-}$ + RV-NRBF2<br>(n = 48)	Two-way ANOVA<br>NRBF2$^{-/-}$: $F(1, 179) = 11.210$, $P = 0.001$<br>RV-NRBF2: $F(1, 179) = 66.080$, $P < 0.001$<br>NRBF2$^{-/-}$ × RV-NRBF2: $F(1, 179) = 2.282$, $P = 0.133$	Bonferroni's post hoc test<br>WT + RV-mCherry vs NRBF2$^{-/-}$ + RV-mCherry, $P < 0.001$;<br>WT + RV-NRBF2 vs NRBF2$^{-/-}$ + RV-NRBF2, $P < 0.001$;<br>WT + RV-mCherry vs WT + RV-NRBF2, $P = 0.393$;<br>NRBF2$^{-/-}$ + RV-mCherry vs NRBF2$^{-/-}$ + RV-NRBF2, $P = 0.001$	WT + RV-mCherry<br>17.403 ± 0.833<br>WT + RV-NRBF2<br>18.802 ± 0.913<br>NRBF2$^{-/-}$ + RV-mCherry<br>10.063 ± 0.584<br>NRBF2$^{-/-}$ + RV-NRBF2<br>13.762 ± 0.670
5b: NRBF2: ATG14L<br>LV-GFP (n = 7)<br>LV-sh*Nrbf*2 (n = 7)	Unpaired t test<br>$t = 6.302$, $P < 0.001$		LV-GFP 1.000 ± 0.050<br>LV-sh*Nrbf2* 0.599 ± 0.039
5c: VPS34: ATG14L<br>LV-GFP (n = 7)<br>LV-sh*Nrbf2* (n = 7)	Unpaired t test<br>$t = 2.971$, $P = 0.012$		LV-GFP 1.000 ± 0.117<br>LV-sh*Nrbf2* 0.582 ± 0.077
5d: Beclin1: ATG14L<br>LV-GFP (n = 7)<br>LV-sh*Nrbf2* (n = 7)	Unpaired t test<br>$t = 1.133$, $P = 0.280$		LV-GFP 1.000 ± 0.041<br>LV-sh*Nrbf2* 0.939 ± 0.035
5e: Ptdlns3P level in NRBF2-deficient aNSCs<br>LV-GFP (n = 8)<br>LV-sh*Nrbf2* (n = 8)	Unpaired t test<br>$t = 4.800$, $P < 0.001$		LV-GFP 1.182 ± 0.105<br>LV-sh*Nrbf*2 0.504 ± 0.094
5g: LC3-II protein expression in NRBF2-deficient aNSCs<br>LV-GFP + Vehicle<br>(n = 9)<br>LV-GFP + BafA1<br>(n = 9)<br>LV-sh*Nrbf2* + Vehicle<br>(n = 9)<br>LV-sh*Nrbf2* + BafA1<br>(n = 9)	Two-way ANOVA<br>LV-sh*Nrbf2*:<br>$F(1, 32) = 19.610$,<br>$P < 0.001$<br>BafA1: $F(1, 32) = 59.740$, $P < 0.001$<br>LV-sh*Nrbf2* × BafA1:<br>$F(1, 32) = 12.500$,<br>$P = 0.001$	Bonferroni's post hoc test<br>LV-GFP + Vehicle vs LV-sh*Nrbf2* + Vehicle, $P = 0.011$;<br>LV-GFP + BafA1 vs LV-sh*Nrbf2* + BafA1, $P < 0.001$;<br>LV-GFP + Vehicle vs LV-GFP + BafA1, $P < 0.001$;<br>LV-sh*Nrbf2* +	LV-GFP + Vehicle<br>1.000 ± 0.029<br>LV-GFP + BafA1<br>1.554 ± 0.122<br>LV-sh*Nrbf2* + Vehicle<br>0.708 ± 0.035<br>LV-sh*Nrbf2* + BafA1<br>0.770 ± 0.049

		Vehicle vs LV-sh*Nrbf2* + BafA1, *P* > 0.999	
5h: p62 protein expression in NRBF2-deficient aNSCs<br>LV-GFP + Vehicle<br>(*n* = 9)<br>LV-GFP + BafA1<br>(*n* = 9)<br>LV-sh*Nrbf2* + Vehicle<br>(*n* = 9)<br>LV-sh*Nrbf2* + BafA1<br>(*n* = 9)	Two-way ANOVA<br>LV-sh*Nrbf2*:<br>F (1, 32) = 12.670,<br>*P* = 0.001<br>BafA1: F (1, 32) = 59.310, *P* < 0.001<br>LV-sh*Nrbf2* × BafA1:<br>F (1, 32) = 5.008,<br>*P* = 0.032	Bonferroni's post hoc test<br>LV-GFP + Vehicle vs LV-sh*Nrbf2* + Vehicle, *P* = 0.001; LV-GFP + BafA1 vs LV-sh*Nrbf2* + BafA1, *P* < 0.001; LV-GFP + Vehicle vs LV-GFP + BafA1, *P* < 0.001; LV-sh*Nrbf2* + Vehicle vs LV-sh*Nrbf2* + BafA1, *P* = 0.714	LV-GFP + Vehicle<br>1.000 ± 0.037<br>LV-GFP + BafA1<br>1.268 ± 0.063<br>LV-sh*Nrbf2* + Vehicle<br>0.747 ± 0.037<br>LV-sh*Nrbf2* + BafA1<br>0.808 ± 0.042
5j: GFP-RFP-LC3 puncta in NRBF2 KD aNSCs<br>CON + Vehicle<br>(*n* = 25)<br>CON + BafA1<br>(*n* = 21)<br>NRBF2 KD + Vehicle<br>(*n* = 25)<br>NRBF2 KD + BafA1<br>(*n* = 25)	Two-way ANOVA<br>NRBF2 KD:<br>F (1, 92) = 20.330,<br>*P* < 0.001<br>BafA1: F (1, 92) = 78.080, *P* < 0.001<br>NRBF2 KD × BafA1:<br>F (1, 92) = 14.920,<br>*P* < 0.001	Bonferroni's post hoc test<br>CON + Vehicle vs NRBF2 KD + Vehicle, *P* = 0.001; CON + BafA1 vs NRBF2 KD + BafA1, *P* < 0.001; CON + Vehicle vs CON + BafA1,<br>*P* < 0.001;<br>NRBF2 KD + Vehicle vs NRBF2 KD + BafA1,<br>*P* > 0.999	CON + Vehicle<br>32.320 ± 2.153<br>CON + BafA1<br>55.143 ± 4.809<br>NRBF2 KD + Vehicle<br>18.760 ± 1.740<br>NRBF2 KD + BafA1<br>20.520 ± 1.829
5k: RFP-LC3 puncta in NRBF2 KD aNSCs<br>CON + Vehicle<br>(*n* = 25)<br>CON + BafA1<br>(*n* = 21)<br>NRBF2 KD + Vehicle<br>(*n* = 25)<br>NRBF2 KD + BafA1<br>(*n* = 25)	Two-way ANOVA<br>NRBF2 KD:<br>F (1, 92) = 6.010,<br>*P* = 0.016<br>BafA1: F (1, 92) = 104.600, *P* < 0.001<br>NRBF2 KD × BafA1:<br>F (1, 92) = 6.824,<br>*P* = 0.011	Bonferroni's post hoc test<br>CON + Vehicle vs NRBF2 KD + Vehicle, *P* < 0.001; CON + BafA1 vs NRBF2 KD + BafA1, *P* < 0.001; CON + Vehicle vs CON + BafA1,	CON + Vehicle<br>48.760 ± 2.727<br>CON + BafA1<br>65.143 ± 5.647<br>NRBF2 KD + Vehicle<br>24.120 ± 2.001<br>NRBF2 KD + BafA1<br>23.600 ± 2.129

		P = 0.001;<br>NRBF2 KD + Vehicle vs NRBF2 KD + BafA1,<br>P > 0.999	
6b: SAR405-SIT<br>Vehicle + CON (n = 10)<br>SAR405 + CON (n = 10)<br>Vehicle + SSDS (n = 9)<br>SAR405 + SSDS (n = 12)	Two-way ANOVA<br>SSDS:<br>F (1, 37) = 13.830,<br>P = 0.001<br>SAR405: F (1, 37) = 12.880, P = 0.001<br>SSDS × SAR405:<br>F (1, 37) = 7.686,<br>P = 0.009	Bonferroni's post hoc test<br>CON + Vehicle vs SSDS + Vehicle,<br>P > 0.999;<br>CON + SAR405 vs SSDS + SAR405,<br>P < 0.001;<br>Vehicle + CON vs SAR405 + CON,<br>P > 0.999;<br>Vehicle + SSDS vs SAR405 + SSDS,<br>P < 0.001	Vehicle + CON<br>1.282 ± 0.110<br>SAR405 + CON<br>1.180 ± 0.128<br>Vehicle + SSDS<br>1.194 ± 0.094<br>SAR405 + SSDS<br>0.496 ± 0.093
6c: SAR405-SPT<br>Vehicle + CON (n = 10)<br>SAR405 + CON (n = 10)<br>Vehicle + SSDS (n = 10)<br>SAR405 + SSDS (n = 12)	Two-way ANOVA<br>SSDS:<br>F (1, 38) = 10.480,<br>P = 0.003<br>SAR405: F (1, 38) = 6.361, P = 0.016<br>SSDS × SAR405:<br>F (1, 38) = 7.100,<br>P = 0.011	Bonferroni's post hoc test<br>CON + Vehicle vs SSDS + Vehicle,<br>P > 0.999;<br>CON + SAR405 vs SSDS + SAR405,<br>P = 0.001;<br>Vehicle + CON vs SAR405 + CON,<br>P > 0.999;<br>Vehicle + SSDS vs SAR405 + SSDS,<br>P < 0.001	Vehicle + CON<br>85.043 ± 1.446<br>SAR405 + CON<br>83.168 ± 2.141<br>Vehicle + SSDS<br>85.510 ± 1.832<br>SAR405 + SSDS<br>66.210 ± 5.028
6d: SAR405-TST<br>Vehicle + CON (n = 10)<br>SAR405 + CON (n = 10)<br>Vehicle + SSDS (n = 10)<br>SAR405 + SSDS (n = 12)	Two-way ANOVA<br>SSDS:<br>F (1, 38) = 6.871,<br>P = 0.013<br>SAR405: F (1, 38) = 7.541, P = 0.009<br>SSDS × SAR405:<br>F (1, 38) = 3.228,<br>P = 0.080	Bonferroni's post hoc test<br>CON + Vehicle vs SSDS + Vehicle,<br>P > 0.999;<br>CON + SAR405 vs SSDS + SAR405,<br>P = 0.004;<br>Vehicle + CON vs SAR405 + CON,	Vehicle + CON<br>98.007 ± 9.866<br>SAR405 + CON<br>111.748 ± 10.494<br>Vehicle + SSDS<br>113.827 ± 22.075<br>SAR405 + SSDS<br>187.446 ± 18.985

		$P > 0.999$;<br>Vehicle + SSDS vs SAR405 + SSDS, $P = 0.006$	
6e: SAR405-FST<br>Vehicle + CON ($n = 10$)<br>SAR405 + CON ($n = 10$)<br>Vehicle + SSDS ($n = 9$)<br>SAR405 + SSDS ($n = 12$)	Two-way ANOVA<br>SSDS:<br>$F(1, 37) = 11.350$, $P = 0.002$<br>SAR405: $F(1, 37) = 7.692$, $P = 0.009$<br>SSDS × SAR405:<br>$F(1, 37) = 1.728$, $P = 0.197$	Bonferroni's post hoc test<br>CON + Vehicle vs SSDS + Vehicle, $P = 0.545$;<br>CON + SAR405 vs SSDS + SAR405, $P = 0.010$;<br>Vehicle + CON vs SAR405 + CON, $P = 0.315$;<br>Vehicle + SSDS vs SAR405 + SSDS, $P = 0.004$	Vehicle + CON<br>83.174 ± 12.726<br>SAR405 + CON<br>105.786 ± 11.063<br>Vehicle + SSDS<br>99.228 ± 13.168<br>SAR405 + SSDS<br>150.763 ± 7.459
6f: SAR405-OFT<br>Vehicle + CON ($n = 10$)<br>SAR405 + CON ($n = 10$)<br>Vehicle + SSDS ($n = 9$)<br>SAR405 + SSDS ($n = 12$)	Two-way ANOVA<br>SSDS:<br>$F(1, 37) = 0.272$, $P = 0.605$<br>SAR405: $F(1, 37) = 0.683$, $P = 0.414$<br>SSDS × SAR405:<br>$F(1, 37) = 0.224$, $P = 0.639$	Bonferroni's post hoc test<br>CON + Vehicle vs SSDS + Vehicle, $P > 0.999$;<br>CON + SAR405 vs SSDS + SAR405, $P = 0.694$;<br>Vehicle + CON vs SAR405 + CON, $P = 0.979$;<br>Vehicle + SSDS vs SAR405 + SSDS, $P > 0.999$	Vehicle + CON<br>34.494 ± 2.123<br>SAR405 + CON<br>32.295 ± 2.338<br>Vehicle + SSDS<br>35.276 ± 2.714<br>SAR405 + SSDS<br>35.168 ± 1.754
6h: LC3-II expression in the DG of SAR405-treated mice<br>Vehicle + SSDS ($n = 7$)<br>SAR405 + SSDS ($n = 7$)	Unpaired t test<br>$t = 2.440$, $P = 0.031$		Vehicle + SSDS<br>1.000 ± 0.116<br>SAR405 + SSDS<br>0.675 ± 0.066
6i: p62 expression in the DG of SAR405-treated mice<br>Vehicle + SSDS ($n = 7$)<br>SAR405 + SSDS ($n = 7$)	Unpaired t test<br>$t = 2.453$, $P = 0.030$		Vehicle + SSDS<br>1.000 ± 0.077<br>SAR405 + SSDS<br>1.386 ± 0.138
6l: $BrdU^+Sox2^+GFAP^+$	Unpaired t test		Vehicle + SSDS

RGLs in the DG of SAR405-treated mice<br>Vehicle + SSDS (n = 4)<br>SAR405 + SSDS (n = 4)<br><br>BrdU^{+}DCX^{+} neuroblasts in DG of SAR405-treated mice<br>Vehicle + SSDS (n = 6)<br>SAR405 + SSDS (n = 6)	t = 3.720, P = 0.010<br><br><br><br><br>Unpaired t test<br>t = 3.938, P = 0.003		1380.000 ± 50. 867<br>SAR405 + SSDS<br>776.250 ± 154.115<br><br><br>Vehicle + SSDS<br>1168.333 ± 61.936<br>SAR405 + SSDS<br>825.000 ± 61.360
7b: cKD-SPT<br>CON (n = 8)<br>cKD (n = 10)	Unpaired t test<br>t = 3.505, P = 0.003		CON 88.712 ± 2.643<br>cKD 72.238 ± 3.621
7c: cKD-TST<br>CON (n = 8)<br>cKD (n = 10)	Unpaired t test<br>t = 2.595, P = 0.020		CON 98.053 ± 19.978<br>cKD 151.101 ± 9.050
7d: cKD-FST<br>CON (n = 8)<br>cKD (n = 10)	Unpaired t test<br>t = 3.890, P = 0.001		CON 50.743 ± 13.892<br>cKD 134.207 ± 15.610
7e: cKD-OFT: total distance<br>CON (n = 8)<br>cKD (n = 10)	Unpaired t test<br>t = 0.661, P = 0.518		CON 39.939 ± 2.223<br>cKD 37.470 ± 2.819
7h: BrdU^{+}Sox2^{+} GFAP^{+} RGLs of NRBF2 cKD mice<br>CON (n = 4)<br>cKD (n = 4)<br><br>BrdU^{+}DCX^{+} neuroblasts of NRBF2 cKD mice<br>CON (n = 4)<br>cKD (n = 4)<br><br>BrdU^{+}NeuN^{+} neurons of NRBF2 cKD mice<br>CON (n = 4)<br>cKD (n = 4)	Unpaired t test<br>t = 5.966, P = 0.001<br><br><br><br><br>Unpaired t test<br>t = 5.832, P = 0.001<br><br><br><br>Unpaired t test<br>t = 4.681, P = 0.003		CON 1380.00 ± 113.80<br>cKD 615.00 ± 59.09<br><br><br><br><br>CON 1412.50 ± 140.26<br>cKD 590.00 ± 14.72<br><br><br><br>CON 1267.50 ± 108.94<br>cKD 723.75 ± 40.33
7i: Membrane potential<br>CON (n = 26)<br>cKD (n = 22)	Unpaired t test<br>t = 0.105, P = 0.917		CON -63.148 ± 1.358<br>cKD -63.329 ± 0.934

7j: Membrane resistance<br>CON (n = 26)<br>cKD (n = 22)	Unpaired t test<br>t = 1.576, P = 0.122		CON 238.513 ± 20.959<br>cKD 189.985 ± 22.531
7l: AP frequency<br>CON (n = 26)<br>cKD (n = 22)	Two-way ANOVA<br>Current injection: F (5, 276) = 39.680, P < 0.001<br>cKD: F (1, 276) = 41.910, P < 0.001<br>Current injection × cKD: F (5, 276) = 1.725, P < 0.128	Bonferroni's post hoc test<br>0 pA: CON vs cKD, P > 0.999<br>30 pA: CON vs cKD, P = 0.632<br>60 pA: CON vs cKD, P = 0.042<br>90 pA: CON vs cKD, P = 0.002<br>120 pA: CON vs cKD, P = 0.001<br>150 pA: CON vs cKD, P = 0.002	0 pA:<br>CON 0.654 ± 0.277<br>cKD 0.000 ± 0.000<br>30 pA:<br>CON 2.462 ± 0.633<br>cKD 0.682 ± 0.363<br>60 pA:<br>CON 4.885 ± 0.860<br>cKD 1.909 ± 0.714<br>90 pA:<br>CON 7.423 ± 0.933<br>cKD 3.500 ± 0.930<br>120 pA:<br>CON 9.808 ± 0.925<br>cKD 5.591 ± 1.023<br>150 pA:<br>CON 11.115 ± 0.982<br>cKD 7.091 ± 0.835
7m: AP threshold<br>CON (n = 26)<br>cKD (n = 22)	Unpaired t test<br>t = 3.110, P = 0.003		CON -45.126 ± 0.717<br>cKD -42.201 ± 0.572
8b: cOE-SIT<br>CON + AAV-mCherry (n = 11)<br>CON + AAV-NRBF2 (n = 11)<br>CSDS + AAV-mCherry (n = 13)<br>CSDS + AAV-NRBF2 (n = 12)	Two-way ANOVA<br>Stress: F (1, 43) = 19.340, P < 0.001<br>AAV-NRBF2: F (1, 43) = 80.630, P < 0.001<br>Stress × AAV-NRBF2: F (1, 43) = 18.320, P < 0.001	Bonferroni's post hoc test<br>CON + AAV-mCherry vs CSDS + AAV-mCherry, P < 0.001;<br>CON + AAV-NRBF2 vs CSDS + AAV-NRBF2, P = 0.004;<br>CON + AAV-mCherry vs CON + AAV-NRBF2, P > 0.999;<br>CSDS + AAV-mCherry vs CSDS + AAV-NRBF2, P < 0.001	CON + AAV-mCherry 1.143 ± 0.074<br>CON + AAV-NRBF2 1.151 ± 0.060<br>CSDS + AAV-mCherry 0.200 ± 0.056<br>CSDS + AAV-NRBF2 0.817 ± 0.089

cOE-TST<br>CON + AAV-mCherry<br>($n = 12$)<br>CON + AAV-NRBF2<br>($n = 12$)<br>CSDS + AAV-mCherry<br>($n = 13$)<br>CSDS + AAV-NRBF2<br>($n = 13$)	Two-way ANOVA<br>Stress: $F(1, 46) = 13.130$, $P = 0.001$<br>AAV-NRBF2: $F(1, 46) = 11.680$, $P = 0.001$<br>Stress × AAV-NRBF2: $F(1, 46) = 2.530$,<br>$P = 0.119$	Bonferroni's post hoc test<br>CON + AAV-mCherry vs CSDS + AAV-mCherry,<br>$P = 0.002$;<br>CON + AAV-NRBF2 vs CSDS + AAV-NRBF2,<br>$P = 0.406$;<br>CON + AAV-mCherry vs CON + AAV-NRBF2,<br>$P = 0.331$;<br>CSDS + AAV-mCherry vs CSDS + AAV-NRBF2,<br>$P = 0.001$	CON + AAV-mCherry<br>103.526 ± 11.920<br>CON + AAV-NRBF2<br>80.170 ± 14.517<br>CSDS + RV-mCherry<br>161.060 ± 8.434<br>CSDS + AAV-NRBF2<br>101.159 ± 10.771
cOE-FST<br>CON + AAV-mCherry<br>($n = 12$)<br>CON + AAV-NRBF2<br>($n = 12$)<br>CSDS + AAV-mCherry<br>($n = 12$)<br>CSDS + AAV-NRBF2<br>($n = 12$)	Two-way ANOVA<br>Stress: $F(1, 44) = 13.330$, $P = 0.001$<br>AAV-NRBF2: $F(1, 44) = 13.080$, $P = 0.001$<br>Stress ×AAV-NRBF2: $F(1, 46) = 4.659$,<br>$P = 0.036$	Bonferroni's post hoc test<br>CON + AAV-mCherry vs CSDS + AAV-mCherry,<br>$P < 0.001$;<br>CON + AAV-NRBF2 vs CSDS + AAV-NRBF2,<br>$P = 0.616$;<br>CON + AAV-mCherry vs CON + AAV-NRBF2,<br>$P = 0.594$;<br>CSDS + AAV-mCherry vs CSDS + AAV-NRBF2,<br>$P < 0.001$	CON + AAV-mCherry<br>64.657 ± 13.441<br>CON + AAV-NRBF2<br>45.852 ± 11.272<br>CSDS + AAV-mCherry<br>135.372 ± 14.175<br>CSDS + AAV-NRBF2<br>62.230 ± 11.212
8d: $BrdU^+Sox2^+GFAP^+$ RGLs of CSDS-exposed Nestin-$CreER^{T2}$ mice<br>CON + AAV-mCherry<br>($n = 4$)<br>CON + AAV-NRBF2	Two-way ANOVA<br>Stress:<br>$F(1, 12) = 2.633$,<br>$P = 0.131$<br>AAV-NRBF2: $F(1, 12) = 22.940$, $P < 0.001$	Bonferroni's post hoc test<br>CON + AAV-mCherry vs CSDS + AAV-mCherry,<br>$P = 0.001$;	CON + AAV-mCherry<br>1327.5 ± 61.695<br>CON + AAV-NRBF2<br>1290.0 ± 147.097

(n = 4)<br>CSDS + AAV-mCherry<br>(n = 4)<br>CSDS + AAV-NRBF2<br>(n = 4)	Stress × AAV-NRBF2: F (1, 12) = 4.075,<br>P = 0.067	CON + AAV-NRBF2 vs CSDS + AAV-NRBF2,<br>P = 0.148;<br>CON + AAV-mCherry vs CON + AAV-NRBF2,<br>P > 0.999;<br>CSDS + AAV-mCherry vs CSDS + AAV-NRBF2,<br>P = 0.049	CSDS + AAV-mCherry<br>682.5 ± 49.181<br>CSDS + AAV-NRBF2<br>1027.5 ± 89.687
BrdU$^+$DCX$^+$ neuroblasts of CSDS-exposed Nestin-CreER^{T2} mice<br><br>CON + AAV-mCherry<br>(n = 4)<br>CON + AAV-NRBF2<br>(n = 4)<br>CSDS + AAV-mCherry<br>(n = 4)<br>CSDS + AAV-NRBF2<br>(n = 4)	Two-way ANOVA<br>Stress: F (1, 12) = 4.796, P = 0.049<br>AAV-NRBF2: F (1, 12) = 32.910, P < 0.001<br>Stress × AAV-NRBF2: F (1, 12) = 3.657,<br>P = 0.080	Bonferroni's post hoc test<br>CON + AAV-mCherry vs CSDS + AAV-mCherry,<br>P < 0.001;<br>CON + AAV-NRBF2 vs CSDS + AAV-NRBF2,<br>P = 0.038<br>CON + AAV-mCherry vs CON + AAV-NRBF2,<br>P > 0.999;<br>CSDS + AAV-mCherry vs CSDS + AAV-NRBF2,<br>P = 0.027	CON + AAV-mCherry<br>1481.25 ± 133.094<br>CON + AAV-NRBF2<br>1515.00 ± 126.541<br>CSDS + AAV-mCherry<br>551.25 ± 58.216<br>CSDS + AAV-NRBF2<br>1050.00 ± 148.366
BrdU$^+$NeuN$^+$ neurons of CSDS-exposed Nestin-CreER^{T2} mice<br><br>CON + AAV-mCherry<br>(n = 5)<br>CON + AAV-NRBF2<br>(n = 5)<br>CSDS + AAV-mCherry<br>(n = 5)<br>CSDS + AAV-NRBF2	Two-way ANOVA<br>Stress:<br>F (1, 15) = 6.116,<br>P = 0.026<br>AAV-NRBF2: F (1, 15) = 11.860, P = 0.004<br>Stress × AAV-NRBF2: F (1, 15) = 5.464,<br>P = 0.034	Bonferroni's post hoc test<br>CON + AAV-mCherry vs CSDS + AAV-mCherry,<br>P = 0.002;<br>CON + AAV-NRBF2 vs CSDS + AAV-NRBF2,<br>P = 0.918;<br>CON + AAV-	CON + AAV-mCherry<br>942.00 ± 68.476<br>CON + AAV-NRBF2<br>951.00 ± 51.439<br>CSDS + AAV-mCherry<br>558.00 ± 56.405<br>CSDS + AAV-NRBF2<br>877.50 ± 91.754

(*n* = 4)		mCherry vs CON + AAV-NRBF2,<br>*P* > 0.999;<br>CSDS + AAV-mCherry vs CSDS + AAV-NRBF2,<br>*P* = 0.010	
8h. Relative mRNA level in the DG of CSDS-exposed mice<br>*Hbb-b1*<br>CON (*n* = 6)<br>CSDS (*n* = 8)<br><br>*Nnt*<br>CON (*n* = 5)<br>CSDS (*n* = 8)<br><br>*Pou4f3*<br>CON (*n* = 6)<br>CSDS (*n* = 6)<br><br>*Apoa1*<br>CON (*n* = 6)<br>CSDS (*n* = 6)<br><br>*Mfge8*<br>CON (*n* = 6)<br>CSDS (*n* = 7)<br><br>*Fabp7*<br>CON (*n* = 6)<br>CSDS (*n* = 7)<br><br>*Farp2*<br>CON (*n* = 6)<br>CSDS (*n* = 7)<br><br>*P3h1*<br>CON (*n* = 6)<br>CSDS (*n* = 8)	Unpaired t test<br>t = 3.810, *P* = 0.003<br><br>Unpaired t test<br>t = 3.237, *P* = 0.008<br><br>Unpaired t test<br>t = 2.335, *P* = 0.042<br><br>Unpaired t test<br>t = 2.904, *P* = 0.016<br><br>Unpaired t test<br>t = 3.369, *P* = 0.006<br><br>Unpaired t test<br>t = 3.865, *P* = 0.003<br><br>Unpaired t test<br>t = 3.621, *P* = 0.004<br><br>Unpaired t test<br>t = 2.282, *P* = 0.042		CON 1.013 ± 0.070<br>CSDS 3.086 ± 0.464<br><br>CON 0.905 ± 0.124<br>CSDS 1.273 ± 0.048<br><br>CON 1.083 ± 0.169<br>CSDS 2.148 ± 0.424<br><br>CON 1.008 ± 0.054<br>CSDS 1.832 ± 0.278<br><br>CON 1.006 ± 0.047<br>CSDS 0.813 ± 0.034<br><br>CON 1.012 ± 0.073<br>CSDS 0.536 ± 0.095<br><br>CON 1.005 ± 0.043<br>CSDS 0.767 ± 0.048<br><br>CON 1.014 ± 0.080<br>CSDS 0.807 ± 0.051
8i: *Mfge8* mRNA level in DG of CSDS-exposed	Two-way ANOVA<br>Stress: F (1, 20) =	Bonferroni's post hoc test	CON + AAV-mCherry 1.007 ± 0.053

mice<br>CON + AAV-mCherry (n = 6)<br>CON + AAV-NRBF2 (n = 6)<br>CSDS + AAV-mCherry (n = 6)<br>CSDS + AAV-NRBF2 (n = 6)	52.090, $P < 0.001$<br>AAV-NRBF2: $F_{(1, 20)} = 2.427$, $P = 0.135$<br>Stress × AAV-NRBF2: $F_{(1, 20)} = 19.980$, $P < 0.001$	CON + AAV-mCherry vs CSDS + AAV-mCherry, $P = 0.001$;<br>CON + AAV-NRBF2 vs CSDS + AAV-NRBF2, $P = 0.105$;<br>CON + AAV-mCherry vs CON + AAV-NRBF2, $P = 0.133$;<br>CSDS + AAV-mCherry vs CSDS + AAV-NRBF2, $P < 0.001$	CON + AAV-NRBF2 1.157 ± 0.047<br>CSDS + AAV-mCherry 0.676 ± 0.032<br>CSDS + AAV-NRBF2 1.317 ± 0.077
8j: *Fabp7* mRNA level in DG of CSDS-exposed mice<br>CON + AAV-mCherry (n = 5)<br>CON + AAV-NRBF2 (n = 5)<br>CSDS + AAV-mCherry (n = 6)<br>CSDS + AAV-NRBF2 (n = 6)	Two-way ANOVA<br>Stress: $F_{(1, 18)} = 33.070$, $P < 0.001$<br>Virus: $F_{(1, 18)} = 1.441$, $P = 0.246$<br>Stress × virus: $F_{(1, 18)} = 8.044$, $P = 0.011$	Bonferroni's post hoc test<br>CON + AAV-mCherry vs CSDS + AAV-mCherry, $P = 0.021$;<br>CON + AAV-NRBF2 vs CSDS + AAV-NRBF2, $P = 0.525$;<br>CON + AAV-mCherry vs CON + AAV-NRBF2, $P = 0.128$;<br>CSDS + AAV-mCherry vs CSDS + AAV-NRBF2, $P < 0.001$	CON + AAV-mCherry 1.042 ± 0.036<br>CON + AAV-NRBF2 1.217 ± 0.056<br>CSDS + AAV-mCherry 0.798 ± 0.046<br>CSDS + AAV-NRBF2 1.316 ± 0.082
8k: *Hbb-b1* mRNA level in DG of CSDS-exposed mice<br>CON + AAV-mCherry (n = 6)<br>CON + AAV-NRBF2 (n = 6)<br>CSDS + AAV-mCherry (n = 6)	Two-way ANOVA<br>Stress: $F_{(1, 20)} = 84.790$, $P < 0.001$<br>AAV-NRBF2: $F_{(1, 20)} = 12.140$, $P = 0.002$<br>Stress × AAV-NRBF2: $F_{(1, 20)} = 28.930$, $P < 0.001$	Bonferroni's post hoc test<br>CON + AAV-mCherry vs CSDS + AAV-mCherry, $P < 0.001$;<br>CON + AAV-NRBF2 vs CSDS + AAV-NRBF2,	CON + AAV-mCherry 1.005 ± 0.047<br>CON + AAV-NRBF2 0.814 ± 0.031<br>CSDS + AAV-mCherry 1.448 ± 0.075<br>CSDS + AAV-NRBF2 0.720 ± 0.034

CSDS + AAV-NRBF2 (*n* = 6)		*P* = 0.391;<br>CON + AAV-mCherry vs CON + AAV-NRBF2,<br>*P* = 0.027;<br>CSDS + AAV-mCherry vs CSDS + AAV-NRBF2,<br>*P* < 0.001	
8l: *Nnt* mRNA level in DG of CSDS-exposed mice<br>CON + AAV-mCherry (*n* = 6)<br>CSDS + AAV-mCherry (*n* = 5)<br>CON + AAV-NRBF2 (*n* = 6)<br>CSDS + AAV-NRBF2 (*n* = 6)	Two-way ANOVA<br>Stress: F (1, 19) = 25.980, *P* < 0.001<br>AAV-NRBF2: F (1, 19) = 17.300, *P* = 0.001<br>Stress × AAV-NRBF2: F (1, 19) = 5.051,<br>*P* = 0.037	Bonferroni's post hoc test<br>CON + AAV-mCherry vs CSDS + AAV-mCherry,<br>*P* = 0.001;<br>CON + AAV-NRBF2 vs CSDS + AAV-NRBF2,<br>*P* = 0.364;<br>CON + AAV-mCherry vs CON + AAV-NRBF2,<br>*P* = 0.106;<br>CSDS + AAV-mCherry vs CSDS + AAV-NRBF2,<br>*P* < 0.001	CON + AAV-mCherry<br>1.014 ± 0.072<br>CON + AAV-NRBF2<br>0.888 ± 0.024<br>CSDS + AAV-mCherry<br>1.297 ± 0.023<br>CSDS + AAV-NRBF2<br>0.973 ± 0.031
S1b: CSDS-social interaction time<br>CON (*n* = 12)<br>CSDS (*n* = 12)	Two-way ANOVA<br>Stress: F (1, 44) = 21.950, *P* < 0.001	Bonferroni's post hoc test<br>Target:<br>CON vs CSDS, *P* < 0.001	No target:<br>CON 52.658 ± 4.705<br>CSDS 51.783 ± 6.150<br>Target:<br>CON 71.075 ± 3.474<br>CSDS 26.158 ± 4.849
S1c: CSDS-SIT<br>CON (*n* = 11)<br>CSDS (*n* = 12)	Unpaired t test<br>t = 8.607, *P* < 0.001		CON 1.258 ± 0.065<br>CSDS 0.492 ± 0.061
S1d: CSDS-SPT<br>CON (*n* = 12)<br>CSDS (*n* = 12)	Unpaired t test<br>t = 8.726, *P* < 0.001		CON 87.131 ± 1.214<br>CSDS 66.962 ± 1.967
S1e: CSDS-TST<br>CON (*n* = 11)<br>CSDS (*n* = 11)	Unpaired t test<br>t = 3.597, *P* = 0.002		CON 88.378 ± 9.392<br>CSDS 142.492 ± 11.755

S1f: CSDS-FST<br>CON (n = 11)<br>CSDS (n = 11)	Unpaired t test<br>t = 3.900, P = 0.001		CON 52.640 ± 9.075<br>CSDS 125.800 ± 16.410
S1g: N*rbf2* mRNA level in the brain<br>(n = 4)			mPFC 1.401 ± 0.185<br>ACC 1.226 ± 0.046<br>CPu 1.136 ± 0.044<br>NAc 1.026 ± 0.115<br>Amygdala 1.210 ± 0.072<br>DG 2.338 ± 0.061<br>CA1 1.757 ± 0.060<br>CA3 1.609 ± 0.118<br>VTA 1. 098 ± 0.063<br>Cerebellum 1.003 ± 0.045<br>OB 1.045 ± 0.059
1h: *p62* mRNA level in DG in CSDS model<br>CON (n = 6)<br>CSDS (n = 6)	Unpaired t test<br>t = 2.765, P = 0.020		CON 1.025 ± 0.100<br>CSDS 0.731 ± 0.035
1j: p62 intensity in DG in CSDS model<br>CON (n = 12)<br>CSDS (n = 12)	Unpaired t test<br>t = 3.274, P = 0.004		CON 1.000 ± 0.071<br>CSDS 0.732 ± 0.040
S1l: VPS34 expression<br>CON (n = 6)<br>CSDS (n = 11)	Unpaired t test<br>t = 0.521, P = 0.610		CON 1.000 ± 0.105<br>CSDS 1.069 ± 0.079
S1m: Beclin1 expression<br>CON (n = 6)<br>CSDS (n = 11)	Unpaired t test<br>t = 0.063, P = 0.951		CON 1.000 ± 0.067<br>CSDS 0.995 ± 0.039
S1n: ATG14L expression<br>CON (n = 6)<br>CSDS (n = 11)	Unpaired t test<br>t = 0.543, P = 0.595		CON 1.000 ± 0.080<br>CSDS 0.959 ± 0.037
S1o: CUS-SPT<br>CON (n = 11)<br>CUS (n = 11)	Unpaired t test<br>t = 4.009, P = 0.001		CON 82.984 ± 2.129<br>CUS 68.701 ± 2.857
S1p: CUS-TST<br>CON (n = 11)<br>CUS (n = 11)	Unpaired t test<br>t = 4.812, P < 0.001		CON 99.167 ± 11.126<br>CUS 169.569 ± 9.498
S1q: CUS-FST<br>CON (n = 11)<br>CUS (n = 11)	Unpaired t test<br>t = 7.143, P < 0.001		CON 61.667 ± 11.525<br>CUS 173.271 ± 10.549

S1r: CORT-SPT<br>CON (n = 9)<br>CORT (n = 11)	Unpaired t test<br>t = 7.142, P < 0.001		CON 84.613 ± 2.227<br>CORT 50.687 ± 3.877
S1s: CORT-TST<br>CON (n = 9)<br>CORT (n = 11)	Unpaired t test<br>t = 7.022, P < 0.001		CON 84.233 ± 6.947<br>CORT 188.642 ± 12.145
S1t: CORT-FST<br>CON (n = 9)<br>CORT (n = 11)	Unpaired t test<br>t = 8.921, P < 0.001		CON 53.811 ± 8.842<br>CORT 169.851 ± 9.266
S3b: $BrdU^{+}Sox2^{+}GFAP^{+}$ RGLs<br>CON (n = 5)<br>CSDS (n = 5)	Unpaired t test<br>t = 5.837, P = 0.001		CON 1320.0 ± 82.432<br>CSDS 717.0 ± 62.282
S3d: $BrdU^{+}DCX^{+}$ neuroblasts<br>CON (n = 5)<br>CSDS (n = 5)	Unpaired t test<br>t = 6.113, P = 0.001		CON 1418.0 ± 87.943<br>CSDS 734.0 ± 69.181
S3f: Dendritic length of newborn neurons<br>CON (n = 42)<br>CSDS (n = 38)	Unpaired t test<br>t = 7.228, P < 0.001		CON 982.5 ± 31.151<br>CSDS 662.2 ± 31.378
S3f: Number of crossings<br>CON (n = 42)<br>CSDS (n = 38)	Repeated measures ANOVA<br>Stress: F (1, 78) = 43.980, P < 0.001		
S4h: Spine density of newborn neurons<br>CON (n = 50)<br>CSDS (n = 50)	Unpaired t test<br>t = 10.090, P < 0.001		CON 21.121 ± 0.947<br>CSDS 10.505 ± 0.459
S4b. NRBF2 protein expression after virus injection<br>CON + LV-vector<br>(n = 8)<br>CON + LV-NRBF2<br>(n = 8)<br>CSDS + LV-vector<br>(n = 11)<br>CSDS + LV-NRBF2<br>(n = 9)	Two-way ANOVA<br>Stress: F (1, 32) = 109.800, P < 0.001<br>Virus: F (1, 32) = 5.581, P = 0.024<br>Stress × Virus: F (1, 32) = 15.510, P < 0.001	Bonferroni's post hoc test<br>CON + AAV-mCherry vs CON + AAV-NRBF2, P < 0.001;<br>CSDS + AAV-VECTOR vs CSDS + AAV-NRBF2, P < 0.001	CON + LV-vector<br>1.000 ± 0.050<br>CON + LV-NRBF2<br>2.528 ± 0.321<br>CSDS + LV-vector<br>0.632 ± 0.072<br>CSDS + LV-NRBF2<br>4.001 ± 0.354
S4c. LC3-II protein expression after virus injection	Two-way ANOVA<br>Stress: F (1, 33) = 12.710, P = 0.001	Bonferroni's post hoc test<br>CON + LV-vector	CON + LV-vector<br>1.000 ± 0.056<br>CON + LV-NRBF2

CON + LV-vector ($n = 9$)<br>CON + LV-NRBF2 ($n = 9$)<br>CSDS + LV-vector ($n = 10$)<br>CSDS + LV-NRBF2 ($n = 9$)	Virus: F (1, 33) = 6.777, $P = 0.014$<br>Stress × Virus: F (1, 33) =11.300, $P = 0.002$	vs CSDS + LV-vector, $P < 0.001$; CON + LV-NRBF2 vs CSDS + LV-NRBF2, $P > 0.999$; CON + LV-vector vs CON + LV-NRBF2, $P = 0.999$; CSDS + LV-vector vs CSDS + LV-NRBF2, $P < 0.001$	1.010 ± 0.065<br>CSDS + LV-vector 0.691 ± 0.039<br>CSDS + LV-NRBF2 1.050 ± 0.046
S4d. p62 protein expression after virus injection<br>CON + LV-vector ($n = 9$)<br>CON + LV-NRBF2 ($n = 9$)<br>CSDS + LV-vector ($n = 10$)<br>CSDS + LV-NRBF2 ($n = 8$)	Two-way ANOVA<br>Stress: F (1, 32) = 11.730, $P = 0.002$<br>Virus: F (1, 32) = 12.610, $P = 0.001$<br>Stress × Virus: F (1, 32) =0.001, $P = 0.980$	Bonferroni's post hoc test<br>CON + LV-vector vs CSDS + LV-vector, $P = 0.028$; CON + LV-NRBF2 vs CSDS + LV-NRBF2, $P = 0.042$; CON + LV-vector vs CON + LV-NRBF2, $P = 0.040$; CSDS + LV-vector vs CSDS + LV-NRBF2, $P = 0.045$	CON + LV-vector 1.000 ± 0.052<br>CON + LV-NRBF2 0.790 ± 0.098<br>CSDS + LV-vector 0.782 ± 0.027<br>CSDS + LV-NRBF2 0.576 ± 0.044
S5b: TMZ-SIT<br>CON + LV-vector + VEH ($n = 9$)<br>CON + LV-vector + TMZ ($n = 9$)<br>CON + LV-NRBF2 + VEH ($n = 8$)<br>CON + LV-NRBF2 + TMZ ($n = 10$)<br>CSDS + LV-vector + VEH ($n = 9$)<br>CSDS + LV-vector + TMZ ($n = 9$)<br>CSDS + LV-NRBF2 + VEH ($n = 9$)<br>CSDS + LV-NRBF2 + TMZ ($n = 10$)	One-way ANOVA<br>F (7, 65) = 7.949, $P < 0.001$	Tukey's post hoc test<br>CON + LV-vector+ VEH vs CSDS + LV-vector + VEH, $P = 0.002$;<br>CSDS + LV-vector + VEH vs CSDS + LV-NRBF2 + VEH, $P = 0.011$<br>CSDS + LV-NRBF2 + TMZ vs CSDS + LV-NRBF2 + VEH, $P = 0.035$	CON + LV-vector + VEH 1.145 ± 0.128<br>CON + LV-vector + TMZ 1.203 ± 0.196<br>CON + LV-NRBF2 + VEH 1.277 ± 0.211<br>CON + LV-NRBF2 + TMZ 0.914 ± 0.102<br>CSDS + LV-vector + VEH 0.234 ± 0.071<br>CSDS + LV-vector + TMZ 0.397 ± 0.174<br>CSDS + LV-NRBF2 + VEH 1.017 ± 0.199<br>CSDS + LV-NRBF2 + TMZ 0.341 ± 0.076
S5b: TMZ-TST	One-way ANOVA	Tukey's post hoc	CON + LV-vector +

CON + LV-vector + VEH ($n = 9$)<br>CON + LV-vector + TMZ ($n = 9$)<br>CON + LV-NRBF2 + VEH ($n = 9$)<br>CON + LV-NRBF2 + TMZ ($n = 10$)<br>CSDS + LV-vector + VEH ($n = 9$)<br>CSDS + LV-vector + TMZ ($n = 9$)<br>CSDS + LV-NRBF2 + VEH ($n = 9$)<br>CSDS + LV-NRBF2 + TMZ ($n = 10$)	$F(7, 66) = 10.110$, $P < 0.001$	test<br>CON + LV-vector+ VEH vs CSDS + LV-vector + VEH, $P = 0.004$<br>CSDS + LV-vector + VEH vs CSDS + LV-NRBF2 + VEH, $P = 0.004$<br>CSDS + LV-NRBF2 + TMZ vs CSDS + LV-NRBF2 + VEH, $P < 0.001$	VEH 86.624 ± 9.765<br>CON + LV-vector + TMZ 114.208 ± 9.246<br>CON + LV-NRBF2 + VEH 99.155 ± 10.552<br>CON + LV-NRBF2 + TMZ 122.171 ± 12.034<br>CSDS + LV-vector + VEH 146.749 ± 5.884<br>CSDS + LV-vector + TMZ 170.467 ± 13.602<br>CSDS + LV-NRBF2 + VEH 86.151 ± 11.704<br>CSDS + LV-NRBF2 + TMZ 165.383 ± 9.699
S5b: TMZ-FST<br>CON + LV-vector + VEH ($n = 9$)<br>CON + LV-vector + TMZ ($n = 9$)<br>CON + LV-NRBF2 + VEH ($n = 9$)<br>CON + LV-NRBF2 + TMZ ($n = 10$)<br>CSDS + LV-vector + VEH ($n = 9$)<br>CSDS + LV-vector + TMZ ($n = 8$)<br>CSDS + LV-NRBF2 + VEH ($n = 9$)<br>CSDS + LV-NRBF2 + TMZ ($n = 10$)	One-way ANOVA<br>$F(7, 65) = 10.840$, $P < 0.001$	Tukey's post hoc test<br>CON + LV-vector + VEH vs CSDS + LV-vector + VEH, $P = 0.001$<br>CSDS + LV-vector + VEH vs CSDS + LV-NRBF2 + VEH, $P = 0.005$<br>CSDS + LV-NRBF2 + TMZ vs CSDS + LV-NRBF2 + VEH, $P = 0.007$	CON + LV-vector + VEH 41.333 ± 13.430<br>CON + LV-vector + TMZ 89.972 ± 15.272<br>CON + LV-NRBF2 + VEH 46.064 ± 12.011<br>CON + LV-NRBF2 + TMZ 71.342 ± 15.044<br>CSDS + LV-vector + VEH 138.239 ± 13.509<br>CSDS + LV-vector + TMZ 177.154 ± 17.354<br>CSDS + LV-NRBF2 + VEH 54.624 ± 15.071<br>CSDS + LV-NRBF2 + TMZ 133.287 ± 16.479
S5b: TMZ-OFT<br>CON + LV-vector + VEH ($n = 9$)<br>CON + LV-vector + TMZ ($n = 9$)<br>CON + LV-NRBF2 + VEH ($n = 9$)<br>CON + LV-NRBF2 + TMZ ($n = 10$)<br>CSDS + LV-vector +	One-way ANOVA<br>$F(7, 66) = 0.698$, $P = 0.674$	Tukey's post hoc test<br>CON + LV-vector + VEH vs CSDS + LV-vector + VEH, $P > 0.999$<br>CSDS + LV-vector + VEH vs CSDS + LV-NRBF2 + VEH, $P > 0.999$	CON + LV-vector + VEH 24.707 ± 2.566<br>CON + LV-vector + TMZ 24.642 ± 2.224<br>CON + LV-NRBF2 + VEH 29.094 ± 3.654<br>CON + LV-NRBF2 + TMZ 28.915 ± 1.577<br>CSDS + LV-vector + VEH 25.036 ± 2.496

VEH ($n = 9$)<br>CSDS + LV-vector + TMZ ($n = 9$)<br>CSDS + LV-NRBF2 + VEH ($n = 9$)<br>CSDS + LV-NRBF2 + TMZ ($n = 10$)		CSDS + LV-NRBF2 + TMZ vs CSDS + LV-NRBF2 + VEH, $P > 0.999$	CSDS + LV-vector + TMZ 24.319 ± 2.717<br>CSDS + LV-NRBF2 + VEH 24.859 ± 2.857<br>CSDS + LV-NRBF2 + TMZ 23.720 ± 2.029
S5d: TMZ-BrdU$^+$ cells<br>CON + LV-vector + VEH ($n = 3$)<br>CSDS + LV-vector + VEH ($n = 3$)<br>CSDS + LV-NRBF2 + VEH ($n = 3$)<br>CSDS + LV-NRBF2 + TMZ ($n = 3$)	One-way ANOVA<br>$F_{(3, 8)} = 19.420$,<br>$P = 0.001$	Tukey's post hoc test<br>CON + LV-vector + VEH vs CSDS + LV-vector + VEH, $P = 0.001$<br>CSDS + LV-NRBF2 + VEH vs CSDS + LV-NRBF2 + TMZ, $P = 0.008$<br>CSDS + LV-NRBF2 + TMZ vs CSDS + LV-NRBF2 + VEH, $P = 0.007$	CON + LV-vector + VEH 2255.0 ± 152.56<br>CSDS + LV-vector + VEH 1070.0 ± 72.11<br>CSDS + LV-NRBF2 + VEH 1965.0 ± 105.36<br>CSDS + LV-NRBF2 + TMZ 1060.0 ± 195.64
S5d: TMZ-BrdU$^+$DCX$^+$ neuroblasts<br>CON + LV-vector + VEH ($n = 3$)<br>CSDS + LV-vector + VEH ($n = 3$)<br>CSDS + LV-NRBF2 + VEH ($n = 3$)<br>CSDS + LV-NRBF2 + TMZ ($n = 3$)	One-way ANOVA<br>$F_{(3, 8)} = 28.090$,<br>$P < 0.001$	Tukey's post hoc test<br>CON + LV-vector + VEH vs CSDS + LV-vector + VEH, $P < 0.001$<br>CSDS + LV-vector + VEH vs CSDS + LV-NRBF2 + VEH, $P = 0.005$<br>CSDS + LV-NRBF2 + TMZ vs CSDS + LV-NRBF2 + VEH, $P = 0.003$	CON + LV-vector + VEH 1300.0 ± 72.110<br>CSDS + LV-vector + VEH 510.0 ± 78.100<br>CSDS + LV-NRBF2 + VEH 1047.0 ± 87.620<br>CSDS + LV-NRBF2 + TMZ 456.7 ± 72.190
S5d: TMZ- BrdU$^+$NeuN$^+$ neuron<br>CON + LV-vector + VEH ($n = 3$)<br>CSDS + LV-vector + VEH ($n = 3$)<br>CSDS + LV-NRBF2 + VEH ($n = 3$)	One-way ANOVA<br>$F_{(3, 8)} = 136.500$,<br>$P < 0.001$	Tukey's post hoc test<br>CON + LV-vector + VEH vs CSDS + LV-vector + VEH, $P < 0.001$<br>CSDS + LV-vector + VEH vs CSDS +	CON + LV-vector + VEH 1256.7 ± 37.118<br>CSDS + LV-vector + VEH 426.7 ± 33.830<br>CSDS + LV-NRBF2 + VEH 883.3 ± 48.419<br>CSDS + LV-NRBF2 + TMZ 383.3 ± 12.019

CSDS + LV-NRBF2 + TMZ ($n = 3$)		LV-NRBF2 + VEH, $P < 0.001$<br>CSDS + LV-NRBF2 + TMZ vs CSDS + LV-NRBF2 + VEH, $P < 0.001$	
S6a: NRBF2$^{-/-}$ mice-OFT: central entries<br>WT ($n = 10$)<br>NRBF2$^{-/-}$ ($n = 9$)	Unpaired t test<br>t = 0.444, $P = 0.663$		WT 73.400 ± 7.820<br>NRBF2$^{-/-}$ 68.889 ± 6.235
S6b: NRBF2$^{-/-}$ mice-OFT: central duration<br>WT ($n = 10$)<br>NRBF2$^{-/-}$ ($n = 9$)	Unpaired t test<br>t = 0.380, $P = 0.709$		WT 105.180 ± 14.991<br>NRBF2$^{-/-}$ 96.444 ± 17.591
S6c: NRBF2$^{-/-}$ mice-OFT: central distance<br>WT ($n = 10$)<br>NRBF2$^{-/-}$ ($n = 9$)	Unpaired t test<br>t = 0.286, $P = 0.779$		WT 5.412 ± 0.451<br>NRBF2$^{-/-}$ 5.227 ± 0.462
S7b: Identification of aNSCs in vitro<br>aNSCs ($n = 6$)	Unpaired t test<br>t = 8.157, $P < 0.001$		Nestin 98.250 ± 0.540<br>Sox2 97.170 ± 0.453<br>NestinSox2 96.690 ± 0.556
S7d: NRBF2 protein expression in NRBF2-deficient aNSCs<br>LV-GFP ($n = 6$)<br>LV-sh*Nrbf2* ($n = 6$)	Unpaired t test<br>t = 8.157, $P < 0.001$		LV-GFP<br>1.000 ± 0.058<br>LV-shNrbf2<br>0.479 ± 0.020
S7d: LC3-II protein expression in NRBF2-deficient aNSCs<br>LV-GFP ($n = 6$)<br>LV-sh*Nrbf2* ($n = 6$)	Unpaired t test<br>t = 5.532, $P < 0.001$		LV-GFP<br>1.000 ± 0.036<br>LV-sh*Nrbf2*<br>0.681 ± 0.045
S7d: p62 protein expression in NRBF2-deficient aNSCs<br>LV-GFP ($n = 6$)<br>LV-sh*Nrbf2* ($n = 6$)	Unpaired t test<br>t = 4.917, $P = 0.001$		LV-GFP<br>1.000 ± 0.052<br>LV-sh*Nrbf2*<br>0.708 ± 0.029
S7f: neurospheres in NRBF2-deficient aNSCs Number<br>LV-GFP (n = 4)<br>LV-sh*Nrbf2* (n = 4)	Unpaired t test<br>t = 4.392, $P = 0.005$		LV-GFP<br>10.000 ± 0.707<br>LV-sh*Nrbf2*<br>6.250 ± 0.479

Diameter<br>LV-GFP (*n* = 4)<br>LV-sh*Nrbf2* (*n* = 4)	Unpaired t test<br>t = 6.847, *P* = 0.001		LV-GFP<br>205.684 ± 9.796<br>LV-sh*Nrbf2*<br>124.240 ± 6.747
S7h: the percent of $BrdU^+GFP^+$ cell in NRBF2-deficient aNSCs<br>LV-GFP (*n* = 3)<br>LV-sh*Nrbf2* (*n* = 3)	Unpaired t test<br>t = 6.327, *P* = 0.003		LV-GFP<br>58.660 ± 3.237<br>LV-sh*Nrbf2*<br>33.805 ± 2.225
S7j: the percent of $Tuj1^+GFP^+$ cell in NRBF2-deficient aNSCs<br>LV-GFP (*n* = 3)<br>LV-sh*Nrbf2* (*n* = 3)<br><br>the percent of $GFAP^+GFP^+$ cell in NRBF2-deficient aNSCs<br>LV-GFP (*n* = 3)<br>LV-sh*Nrbf2* (*n* = 3)	Unpaired t test<br>t = 5.064, *P* = 0.007<br><br>Unpaired t test<br>t = 0.390, *P* = 0.717		LV-GFP<br>39.653 ± 0.629<br>LV-sh*Nrbf2*<br>27.338 ± 2.349<br><br>LV-GFP<br>35.047 ± 1.393<br>LV-sh*Nrbf2*<br>35.737 ± 1.094
S7l: the percent of $AC3^+GFP^+$ cell in NRBF2-deficient aNSCs<br>LV-GFP (*n* = 3)<br>LV-sh*Nrbf2* (*n* = 3)<br><br>the percent of $Tuj1^+AC3^+GFP^+$ cells in NRBF2-deficient aNSCs<br>LV-GFP (*n* = 3)<br>LV-sh*Nrbf2* (*n* = 3)	Unpaired t test<br>t = 0.108, *P* = 0.920<br><br>Unpaired t test<br>t = 0.765, *P* = 0.487		LV-GFP<br>1.268 ± 0.180<br>LV-sh*Nrbf2*<br>1.299 ± 0.223<br><br>LV-GFP<br>7.902 ± 0.641<br>LV-sh*Nrbf2*<br>8.468 ± 0.368
S7n: Cell viability in NRBF2- deficient aNSCs<br>LV-GFP + Vehicle<br>(*n* = 6)<br>LV-GFP + BafA1<br>(*n* = 6)<br>LV-sh*Nrbf2* + Vehicle<br>(*n* = 6)<br>LV-sh*Nrbf2* + BafA1<br>(*n* = 6)	Two-way ANOVA<br>LV-sh*Nrbf2*:<br>F (1, 20) = 1.547,<br>*P* = 0.228<br>BafA1: F (1, 20) = 2.855, *P* = 0.107<br>LV-sh*Nrbf2* × BafA1:<br>F (1, 20) = 2.352,<br>*P* = 0.141	Bonferroni's post hoc test<br>LV-GFP + Vehicle vs LV-sh*Nrbf2* + Vehicle, *P* = 0.068; LV-GFP + BafA1 vs LV-sh*Nrbf2* + BafA1, *P* > 0.999; LV-GFP + Vehicle vs LV-GFP + BafA1, *P* = 0.127; LV-sh*Nrbf2* +	LV-GFP + Vehicle<br>100.000 ± 0.567<br>LV-GFP + BafA1<br>96.953 ± 1.336<br>LV-sh*Nrbf2* + Vehicle<br>96.465 ± 1.299<br>LV-sh*Nrbf2* + BafA1<br>96.782 ± 1.009

		Vehicle vs LV-sh*Nrbf2* + BafA1, $P > 0.999$	
S8a: NRBF2 expression in aNSCs treated with LV-GFP or LV-NRBF2<br>LV-GFP ($n = 6$)<br>LV-NRBF2 ($n = 6$)	Unpaired t test<br>$t = 13.360$, $P < 0.001$		LV-GFP 1.000 ± 0.109<br>LV-NRBF2 3.600 ± 0.161
S8c: LC3-II expression in SAR405-treated aNSCs<br>Vehicle + LV-GFP ($n = 6$)<br>Vehicle + LV-NRBF2 ($n = 6$)<br>SAR405 + LV-GFP ($n = 6$)<br>SAR405 + LV-NRBF2 ($n = 6$)	Two-way ANOVA<br>SAR405: $F (1, 20) = 19.740$, $P < 0.001$<br>Virus: $F (1, 20) = 11.080$, $P = 0.003$<br>SAR405 × Virus: $F (1, 20) = 3.428$, $P = 0.079$	Bonferroni's post hoc test<br>Vehicle + LV-GFP vs SAR405 + LV-GFP, $P = 0.003$<br>Vehicle + LV-NRBF2 vs SAR405 + LV-NRBF2, $P = 0.617$<br>Vehicle + LV-GFP vs Vehicle + LV-NRBF2, $P = 0.164$<br>SAR405 + LV-GFP vs SAR405 + LV-NRBF2, $P = 0.001$	Vehicle + LV-GFP<br>1.000 ± 0.067<br>Vehicle + LV-NRBF2<br>1.395 ± 0.128<br>SAR405 + LV-GFP<br>0.630 ± 0.044<br>SAR405 + LV-NRBF2<br>1.080 ± 0.113
S8d: p62 expression in SAR405-treated aNSCs<br>Vehicle + LV-GFP ($n = 6$)<br>Vehicle + LV-NRBF2 ($n = 6$)<br>SAR405 + LV-GFP ($n = 6$)<br>SAR405 + LV-NRBF2 ($n = 6$)	Two-way ANOVA<br>SAR405: $F (1, 20) = 22.060$, $P < 0.001$<br>Virus: $F (1, 20) = 41.430$, $P < 0.001$<br>SAR405 × Virus: $F (1, 20) = 4.173$, $P = 0.055$	Bonferroni's post hoc test<br>Vehicle + LV-GFP vs SAR405 + LV-GFP, $P < 0.001$<br>Vehicle + LV-NRBF2 vs SAR405 + LV-NRBF2, $P = 0.011$<br>Vehicle + LV-GFP vs Vehicle + LV-NRBF2, $P = 0.151$<br>SAR405 + LV-GFP vs SAR405 + LV-NRBF2, $P < 0.001$	Vehicle + LV-GFP<br>1.000 ± 0.050<br>Vehicle + LV-NRBF2<br>0.834 ± 0.052<br>SAR405 + LV-GFP<br>1.531 ± 0.079<br>SAR405 + LV-NRBF2<br>1.109 ± 0.065
S8f: the precent of $BrdU^+GFP^+$ cells in SAR405-treated aNSCs<br>Vehicle + LV-GFP ($n = 4$)	Two-way ANOVA<br>SAR405: $F (1, 12) = 5.473$, $P = 0.037$<br>Virus: $F (1, 12) = 12.100$, $P = 0.005$	Bonferroni's post hoc test<br>Vehicle + LV-GFP vs SAR405 + LV-GFP, $P = 0.003$	Vehicle + LV-GFP<br>51.231 ± 2.290<br>Vehicle + LV-NRBF2<br>51.181 ± 3.728<br>SAR405 + LV-GFP

Vehicle + LV-NRBF2 (*n* = 4)<br>SAR405 + LV-GFP (*n* = 4)<br>SAR405 + LV-NRBF2 (*n* = 4)	SAR405 × Virus: F (1, 12) = 5.553, *P* = 0.036	Vehicle + LV-NRBF2 vs SAR405 + LV-NRBF2, *P* = 0.886<br>Vehicle + LV-GFP vs Vehicle + LV-NRBF2, *P* > 0.999<br>SAR405 + LV-GFP vs SAR405 + LV-NRBF2, *P* = 0.012	34.166 ± 2.059<br>SAR405 + LV-NRBF2 47.899 ± 3.290
S9b: NRBF2 expression in SSDS-treated mice<br>CON (*n* = 7)<br>SSDS (*n* = 7)	Unpaired t test<br>t = 0.020, *P* = 0.985		CON 1.000 ± 0.093<br>SSDS 1.003 ± 0.094
S9c: LC3-II expression in SSDS-treated mice<br>CON (*n* = 7)<br>SSDS (*n* = 7)	Unpaired t test<br>t = 0.572, *P* = 0.578		CON 1.000 ± 0.082<br>SSDS 0.935 ± 0.079
S9d: p62 expression in SSDS-treated mice<br>CON (*n* = 7)<br>SSDS (*n* = 7)	Unpaired t test<br>t = 0.291, *P* = 0.776		CON 1.000 ± 0.077<br>SSDS 1.030 ± 0.068
S10c: NRBF2 expression in NRBF2 cKD mice<br>AAV-mCherry (*n* = 5)<br>AAV-sh*Nrbf2* (*n* = 5)	Unpaired t test<br>t = 3.521, *P* = 0.008		AAV-mCherry<br>1.000 ± 0.075<br>AAV-sh*Nrbf2*<br>0.541 ± 0.107
S10d: LC3-II expression in NRBF2 cKD mice<br>AAV-mCherry (*n* = 5)<br>AAV-sh*Nrbf2* (*n* = 5)	Unpaired t test<br>t = 4.197, *P* = 0.003		AAV-mCherry<br>1.000 ± 0.059<br>AAV-sh*Nrbf2*<br>0.665 ± 0.053
S11b: LC3 puncta within $Nestin^+$ cells in DG of CSDS-exposed mice<br>CON (*n* = 33)<br>CSDS (*n* = 33)	Unpaired t test<br>t = 5.530, *P* < 0.001		CON 14.970 ± 1.079<br>CSDS 7.970 ± 0.662
S12c: NRBF2 expression<br>AAV-mCherry (*n* = 4)<br>AAV-NRBF2 (*n* = 4)	Unpaired t test<br>t = 2.593, *P* = 0.041		AAV-mCherry<br>1.000 ± 0.099<br>AAV-sh*Nrbf2*<br>1.409 ± 0.123
S12d: LC3-II expression<br>AAV-mCherry (*n* = 4)<br>AAV-NRBF2 (*n* = 4)	Unpaired t test<br>t = 3.449, *P* = 0.014		AAV-mCherry<br>1.000 ± 0.091<br>AAV-NRBF2<br>1.665 ± 0.170

S13h: MFGE8 peptides in NRBF2$^{-/-}$ mice<br>WT (*n* = 3)<br>NRBF2$^{-/-}$ (*n* = 3)	Peptide 1: Unpaired t test,<br>t = 4.257, *P* = 0.013<br>Peptide 2: Unpaired t test,<br>t = 8.362, *P* = 0.001		Peptides 1:<br>WT 1.177 ± 0.058<br>NRBF2$^{-/-}$ 0.825 ± 0.060<br>Peptides 2:<br>WT 1.130 ± 0.007<br>NRBF2$^{-/-}$ 0.870 ± 0.028
S13i: MFGE8 expression in DG of NRBF2$^{-/-}$ mice<br>WT (*n* = 4)<br>NRBF2$^{-/-}$ (*n* = 4)	Unpaired t test<br>t = 4.134, *P* = 0.006		WT 1.000 ± 0.087<br>NRBF2$^{-/-}$ 0.587 ± 0.048
S13j: MFGE8 expression in NRBF2-deficient aNSCs<br>LV-GFP (*n* = 6)<br>LV-sh*Nrbf2* (*n* = 6)	Unpaired t test<br>t = 4.552, *P* = 0.001		LV-GFP 1.000 ± 0.068<br>LV-sh*Nrbf2* 0.593 ± 0.058
S13k: MFGE8 expression in DG after CSDS<br>CON (*n* = 8)<br>CSDS (*n* = 8)	Unpaired t test<br>t = 3.468, *P* = 0.004		CON 1.000 ± 0.126<br>CSDS 0.465 ± 0.089

226
